# Drivers of unprofessional behaviour between staff in acute care hospitals: a realist review

**DOI:** 10.1186/s12913-023-10291-3

**Published:** 2023-11-30

**Authors:** Justin Avery Aunger, Jill Maben, Ruth Abrams, Judy M. Wright, Russell Mannion, Mark Pearson, Aled Jones, Johanna I. Westbrook

**Affiliations:** 1 School of Health Sciences, Faculty of Health and Medical Sciences, University of Surrey, Guildford, UK; 2 Institute of Applied Health Research, University of Birmingham, Birmingham, UK; 3 NIHR Midlands Patient Safety Research Collaboration, University of Birmingham, Birmingham, UK; 4 School of Medicine, Faculty of Medicine and Health, University of Leeds, Leeds, UK; 5 Health Services Management Centre, University of Birmingham, Birmingham, UK; 6 Wolfson Palliative Care Research Centre, Hull York Medical School, University of Hull, Hull, UK; 7 School of Nursing and Midwifery, Faculty of Health, University of Plymouth, Plymouth, UK; 8 Australian Institute of Health Innovation, Macquarie University, Sydney, NSW Australia

**Keywords:** Bullying, Incivility, Unprofessional behaviour, Organisational culture, Workforce, Acute health care, Professionalism, Patient safety, Psychological wellbeing, Psychological safety

## Abstract

**Background:**

Unprofessional behaviours (UB) between healthcare staff are rife in global healthcare systems, negatively impacting staff wellbeing, patient safety and care quality. Drivers of UBs include organisational, situational, team, and leadership issues which interact in complex ways. An improved understanding of these factors and their interactions would enable future interventions to better target these drivers of UB.

**Methods:**

A realist review following RAMESES guidelines was undertaken with stakeholder input. Initial theories were formulated drawing on reports known to the study team and scoping searches. A systematic search of databases including Embase, CINAHL, MEDLINE and HMIC was performed to identify literature for theory refinement. Data were extracted from these reports, synthesised, and initial theories tested, to produce refined programme theories.

**Results:**

We included 81 reports (papers) from 2,977 deduplicated records of grey and academic reports, and 28 via Google, stakeholders, and team members, yielding a total of 109 reports. Five categories of contributor were formulated: (1) workplace disempowerment; (2) harmful workplace processes and cultures; (3) inhibited social cohesion; (4) reduced ability to speak up; and (5) lack of manager awareness and urgency. These resulted in direct increases to UB, reduced ability of staff to cope, and reduced ability to report, challenge or address UB. Twenty-three theories were developed to explain how these contributors work and interact, and how their outcomes differ across diverse staff groups. Staff most at risk of UB include women, new staff, staff with disabilities, and staff from minoritised groups. UB negatively impacted patient safety by impairing concentration, communication, ability to learn, confidence, and interpersonal trust.

**Conclusion:**

Existing research has focused primarily on individual characteristics, but these are inconsistent, difficult to address, and can be used to deflect organisational responsibility. We present a comprehensive programme theory furthering understanding of contributors to UB, how they work and why, how they interact, whom they affect, and how patient safety is impacted. More research is needed to understand how and why minoritised staff are disproportionately affected by UB.

**Study registration:**

This study was registered on the international database of prospectively registered systematic reviews in health and social care (PROSPERO): https://www.crd.york.ac.uk/prospero/display_record.php?ID=CRD42021255490.

**Supplementary Information:**

The online version contains supplementary material available at 10.1186/s12913-023-10291-3.

## Introduction

Unprofessional behaviours (UB) between healthcare staff are endemic in healthcare workplaces globally and have a deleterious impact on staff wellbeing and patient safety [1, 2]. Including a range of behaviours such as incivility, microaggressions, harassment, and bullying, UB can be defined as “a*ny interpersonal behaviour by staff that causes distress or harm to other staff in the healthcare workplace*” [3]. Rates of UB globally differ quite significantly [4–8]. For example, in a single Portuguese hospital, prevalence of bullying was reported to be 8% [7], whereas, in Italy, according to an online survey of healthcare workers, prevalence has been found to be 12.3% for men but 16.4% for women [4]. Prevalence can vary depending on: how UBs are defined and measured; from hospital to hospital; within organisations; and between demographic groups [1, 9]. Unfortunately, data consistently demonstrate that women, staff with a disability, and those who are from ethnic minority backgrounds, experience UB to a much greater extent [10]. For example, in 2022, the UK’s NHS Workforce Race Equality Standard data revealed that 27.6% of black, minority and ethnic (BME) staff respondents experienced UB over the past 12 months, compared with only 22.5% of white respondents [10].

Existing research into UBs between healthcare staff has been explored using a range of study designs, and this research includes investigations into the ‘causes’ of UB [11–13]. Causes of UB have been referred to as determinants, triggers, predictors, precipitating factors, and more [14, 15]. In this review, we adopt the terms ‘contributors’ and 'drivers' to reflect the fact that some factors may lead to UB in some cases but not in others (i.e., that it is not simply deterministic or predictable). Prior reviews have sought to understand the causes, or contributors, to UB in healthcare [14, 16–22] and to develop theories to explain how UB develops [23]; for example, one realist synthesis from 2013 investigated causes of bullying between healthcare staff [24]. These contributors to UB vary from publication to publication. One review categorised the causes of UB in healthcare into individual characteristics of initiators of UB, characteristics of the targets of UB, professional groups and their characteristics, and situational and cultural determinants [14]. However, the aforementioned realist review from 2013 considered categories of individual characteristics of perpetrators and targets (e.g. lack of emotional control, levels of aggression), social dynamics (e.g. learned behaviours), and the organisational context (e.g. organisational change and work design) [24]. Drawing together these myriad factors into a coherent understanding of how UB develops and why is a complex but necessary challenge.

The impact of UBs must also be considered. It has long been acknowledged that the presence of UB in the healthcare workplace has a significant negative impact on patient safety [2, 25, 26]. Simulation studies of clinical scenarios have demonstrated that rudeness can explain 12% of variance in diagnostic and procedural performance, and inhibits information-sharing and help-seeking behaviours according to a predictive statistical model [26, 27]. Likewise, a simulated ‘discouraging’ environment can lead to trainees speaking up to report medical mistakes less frequently (30%) when compared to an encouraging environment (82%) [28]. In practice, data show that surgeons who are reported to engage more frequently in UB by their co-workers have a higher complication rate (14.3% higher) [29]. While aspects of how and why UB negatively impacts patient safety have been highlighted in the literature, a greater understanding of the mechanisms underlying this causal pathway would enable better-targeted interventions to reduce UB in workplace settings.

The presence of UB also negatively impacts the wellbeing of staff who experience or are witness to it [1]. Experiencing UB has been associated with a greater incidence of burnout [30], intention to leave and absences from work [31], and in severe cases, suicidal ideation [22]. An analysis of survey data from 512 medical staff in seven Australian hospitals found staff who were exposed to rudeness more frequently became more vulnerable to it, and that being a woman, working at a public hospital, or having a lower professional status, could worsen this vulnerability [32]. Individuals bear the brunt of the impact of UB, yet, cumulatively, these impacts also affect the organisations in which they work. While organisations have a legal responsibility to provide a safe working environment, failure to do so negatively impacts organisations due to the aforementioned patient safety and care quality implications, staff absences or resigning, and reputational damage [33]. These costs are significant; for example, an evaluation of the Civility, Respect, and Engagement in the Workplace (CREW) intervention in a hospital in the USA identified a 38% decrease in staff absences post-intervention to reduce UB in the workplace [31]. Total staff absences were estimated to cost $25 million annually in one of the five hospitals included in this study, demonstrating the significant cost savings such interventions could yield [31]. A further estimate in the US from 2013 suggests a 400 bed hospital incurs $1 million in cost per year due to UB [34].

By drawing on a realist review methodology, we aimed to build an understanding of how and why various contributors lead to UB, how these contributors interact, and how UB leads to negative impacts on patients and staff. We intended for these findings to enable the design of better interventions to target contributors to UB in healthcare workplaces [35].

## Methods

### Rationale for, and use of, realist methods

Realist reviews typically develop understanding of why an intervention may work in one context but not another. This involves building an understanding of how various contextual factors affect the activation of mechanisms (i.e. changes in participant reasoning) to produce various outcomes [36]. Realist research uses retroductive reasoning (“*identification of hidden causal forces that lie behind identified patterns or changes in those patterns*” [37]) to unpack often poorly articulated information about how and why interventions may work. This is combined with inductive and deductive reasoning, as well as ‘hunches’ to ask “why do things appear as they do?” [38]. This is done by first developing an initial programme theory representing how and why an intervention may work, before drawing on a wider body of literature to test and refine findings against this initial theory [39, 40].

This study is part of a larger realist review which also considers interventions that attempt to reduce, mitigate, and prevent UB [3, 35]. Our analysis of the contributors to UB suggests that contributors can be circumstances that ‘naturally’ exist in an organisation (e.g., hierarchies, workload, etc.). These contributors can be considered the contexts which alter the activation of mechanisms to either produce UB or exacerbate its negative effects [41]. We have thus applied realist logic to build programme theories depicted through context-mechanism-outcome configurations (CMOCs) to represent an understanding of how different contexts (i.e., contributors) may change mechanisms (staff reasoning) to affect the incidence or impact of UB (outcomes).

This review followed the Realist and Meta-Review Evidence Synthesis: Evolving Standards (RAMESES) publication standards [42]. The protocol for this review has been previously peer reviewed and published [43]. A RAMESES checklist is available in Additional File 1.

### Aim

The aim of this review was to “*Develop and refine context, mechanism and outcome configurations (CMOCs): to understand the contributors to and contexts of unprofessional behaviours; the mechanisms which trigger different behaviours; and the outcomes on staff, patients and wider system of healthcare*”.

### Review process

Figure 2 provides an overview of the six steps of the realist review which are described in more detail below. Key realist methodology terms are defined in (Table 1).

1. Building initial programme theories. We started by searching for UB policy and guidance literature (reports) on organisational websites such as NHS England, King’s Fund, BMA, HCPC, and NHS Employers websites, to build initial programme theories, as well as using literature known to the project team or that was in the protocol. This helped explore the variety and scope of strategies for addressing UB in acute healthcare settings. To develop initial CMOCs, we examined literature describing how and why each contributor may worsen UB. We organised and coded this data in NVivo12, focusing on finding out how strategies work in different contexts. We also created ‘if, then, because’ statements for each contributor iteratively within the team. These initial programme theories (IPTs) are presented in Additional File 2.
2. Searching for evidence (iterative). From November 2021 to December 2022, we sought evidence in published and grey literature for use in testing and refining this IPT. We searched Embase, CINAHL and MEDLINE databases for published literature and HMIC, NICE Evidence Search, Patient Safety Network, Google and Google Scholar databases, and NHS Employers and NHS Health Education England websites for grey literature. Additional File 3 contains the full search process and strategy details.
3. Report selection. We selected reports based on inclusion criteria, rigour, and relevance (including conceptual richness). JA screened the search results and RA checked 10% of them randomly at title and abstract, full text, and relevancy stages. JAA, RA, and JM discussed any disagreements. JAA made the decisions for the other 90% of the results at these stages. We used Rayyan.ai software (http://www.rayyan.ai/) for title and abstract screening and Mendeley (Mendeley Ltd) [44] for full text screening. We also used realist relevancy and rigour criteria, as well as adapted criteria from Pearson et al. (2015) [45] to assess conceptual richness and select the most theoretically useful literature. Inclusion criteria were as follows (Table 2):

**Table 1:** Definitions of realist concepts as used in this paper

**Realist term**	**Operational definition**
Context	Aspects of the setting in which UB occurs which affect how mechanisms are triggered. This can include geographical, social, resource, participant, or other features [40, 46]
Context–mechanism–outcome configurations (CMOCs)	A realist heuristic which enables an understanding of generative causation. This is typically constructed as “*an outcome (O) of interest was generated by relevant mechanism(s) (M) being triggered in specific context(s) (C)”* [40]
Demi-regularity	*“semi-predictable patterns or pathways of programme functioning”* [40]
Mechanisms	“… *mechanisms are a combination of resources offered by the social programme under study and stakeholders’ reasoning in response*” [41]
Programme theory	“*A set of theoretical explanations or assumptions about how a particular programme, process or interventions is expected to work*” [37] Initial programme theories are those created at the start of the analysis process from a limited amount of literature and stakeholder input. Refined programme theories are those that arise from the end of the analysis process after a process of comparison, refutation, consolidation, and creation as necessary
Retroduction	“*Identification of hidden causal forces that lie behind identified patterns or changes in those patterns*” [37]
Outcomes	“*Outcomes are any intended or unintended changes in individuals, teams or organisational culture generated by context-mechanism interactions*” [47]. These can be proximal and distal in the causal chain

**Table 2:** Inclusion criteria

**Category**	**Criterion**
Study design	Any (including non-empirical papers/editorials reports)
Study setting	Acute healthcare settings—acute, critical, emergency (and potentially wider, see relevance criteria below)
Types of UB	All as exhibited and experienced by healthcare staff towards other staff (not towards patients nor patient to staff)
Types of participants	Employed staff groups including students on placements
Types of contributor to UB	Any
Outcomes	Included but not limited to a focus on one or more of: staff wellbeing (stress, burnout, resilience) staff turnover, absenteeism, malpractice claims, patient complaints, magnet hospital/recruitment, patient safety (avoidable harm, errors, speaking up rates, safety incidents, improved listening/response), cost
Language	English only

We included reports based on the criteria (above) and applied both relevance and rigour criteria [48]. Relevance depended on how well the reports met the major/minor criteria below and how they could inform programme theories.

Assessment of rigour depended on how well the reports described their methods and how reliable and generalisable their findings were based on those methods [40, 48]. Data organisation in NVivo enabled traceability from data source through to final programme theories. Rigour was therefore assessed at both the level of data source (i.e. trustworthiness of data sources, hence two entries being excluded due to lack of trustworthiness, see Fig. 3) as well as at the level of the programme theories (i.e. programme theory coherency) [48]. All final programme theories relied on multiple sources of evidence and at least one peer-reviewed article.

**Fig. 1:**
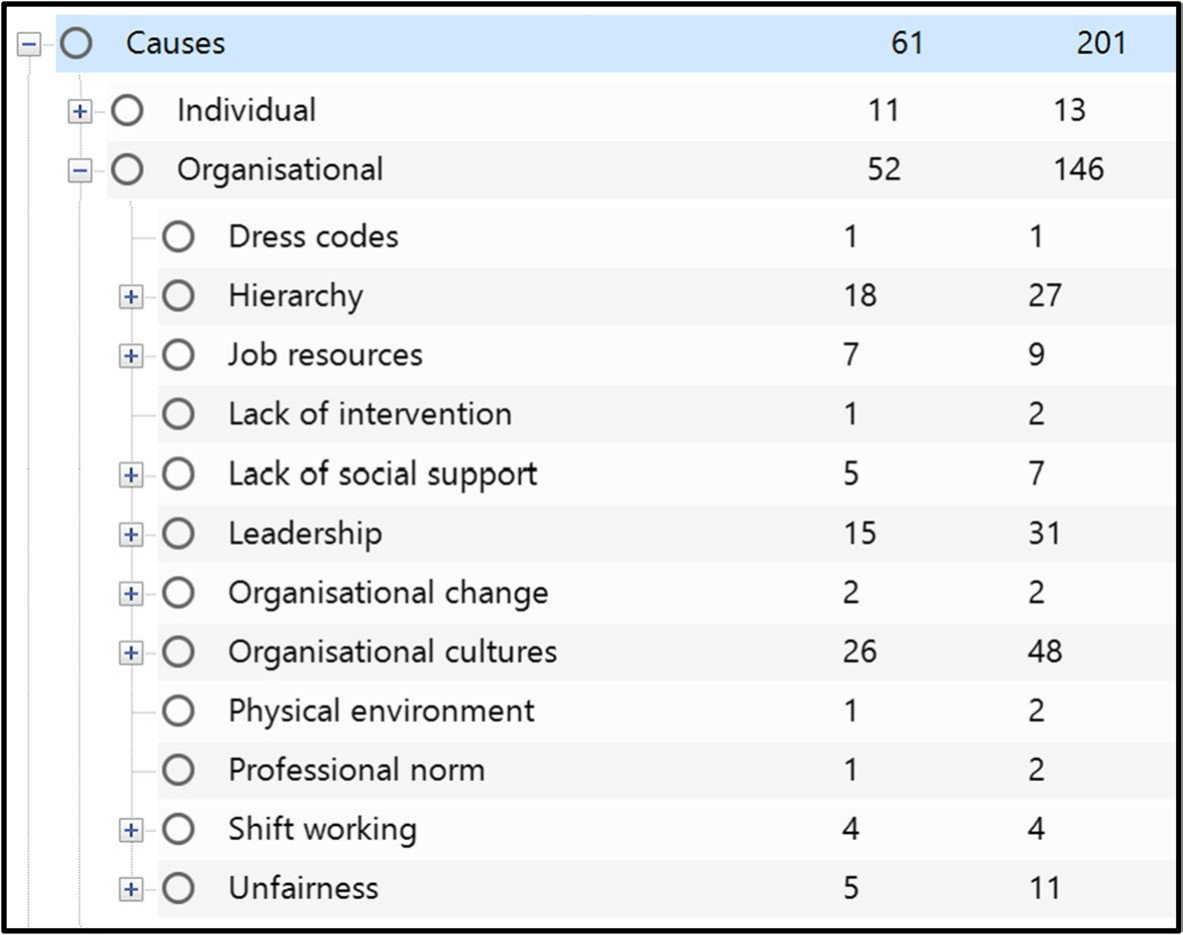
Example coding structure for contributors to UB in Step 4

Our formal criteria for classifying the relevance of reports are below. To be included, reports must have:

- contributed to the study aims and been conducted in an NHS context in acute care; or,
- contributed to the study aims and been conducted in an NHS context; or,
- contributed to the study aims and been conducted in contexts with similarities to the NHS (e.g., universal, publicly funded health-care systems);
- for intervention studies: been conducted in UK or non-UK health-care systems that are markedly different to the NHS (e.g., fee-for-service, private insurance scheme systems) but where the mechanisms causing or moderating UBs could plausibly operate in the context of those working in the NHS.

4) Data extraction. We imported PDF files for all reports into NVivo12 software (QSR International) for data extraction. We used NVivo to sort and categorise the data using a combination of inductive and deductive codes in line with guidance for use of NVivo in other realist syntheses [49, 50]. We created codes for each contributor that we found in the literature so that we could develop theories for each of them in the next analysis step (Fig. 1). We also extracted key excerpts for demi-regularities (*semi-predictable patterns or pathways of programme functioning”* [40]) that we noticed across studies into a separate Microsoft Word document to collect and examine patterns across literature to assist in understanding how contributors interact. Additionally, we transferred key characteristics of the included reports into a Microsoft Excel spreadsheet. This table of characteristics of included reports can be found in Additional File 4.
5) Synthesis. Data synthesis with use of NVivo enabled us to compare and contrast, reconcile, adjudicate, and consolidate different sources of evidence to build an understanding of how contributors to UB work and why. Identifying demi-regularities (or “*semi-predictable patterns or pathways of programme functioning*” [37]) across studies enabled us to categorise, by common underlying mechanisms, these contributors to UB. It also enabled us to identify how contributors interact with each other, enabling us to formulate a full, coherent programme theory that draws the contributors together.
6) Testing and refining programme theories. Through comparison against this literature from Step 2 onwards, the initial programme theories were tested, confirmed, refuted, or new theories developed and added to our analysis.

### Stakeholder and patient and public involvement

**Fig. 2:**
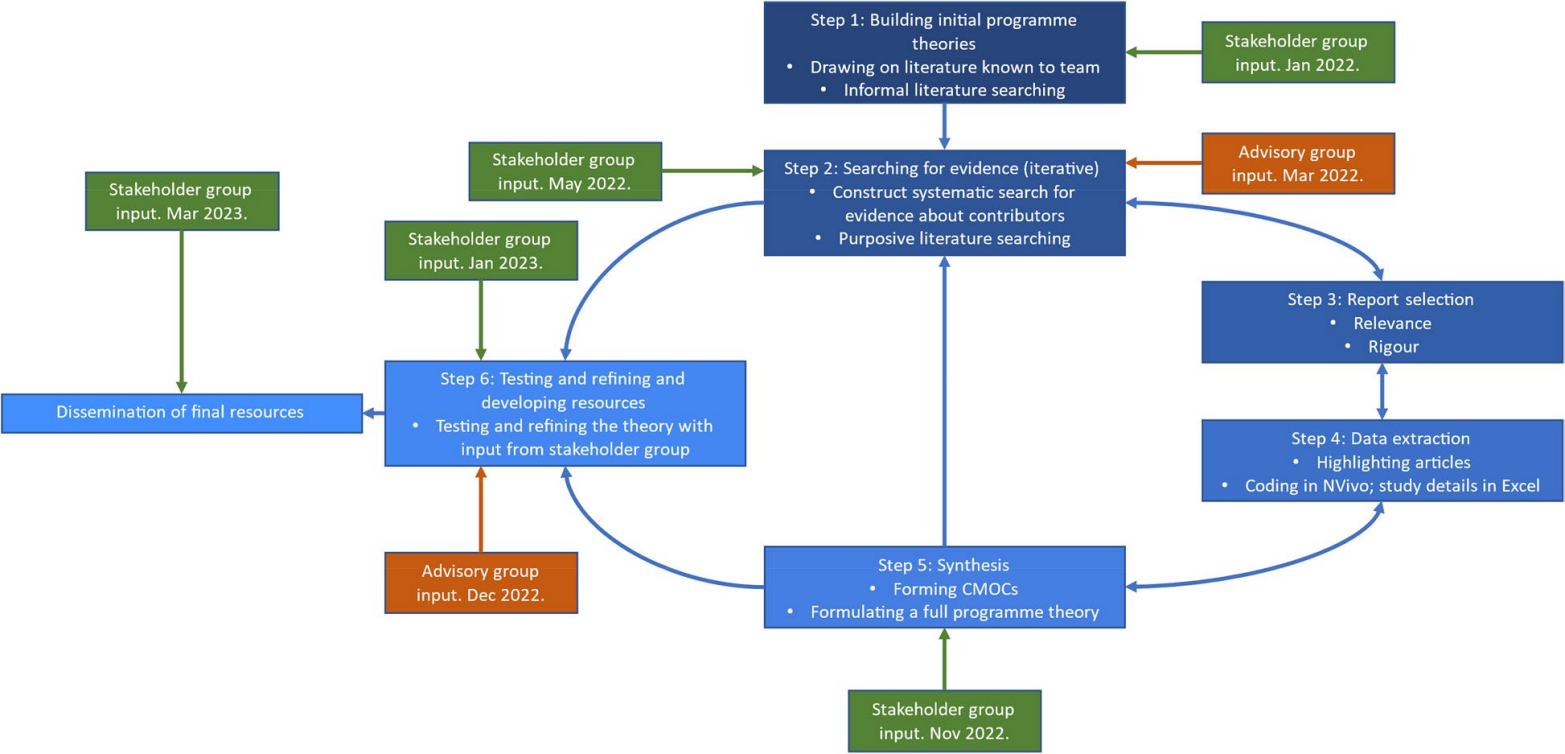
Flow diagram for realist review process. Updated from Maben et al. (2023) [43]

Stakeholder feedback was incorporated at five stages (Fig. 2). Stakeholders and advisors included senior staff of professional standards bodies, members of regulatory bodies and trade unions in the UK, patients and members of the public from diverse backgrounds, academics with expertise in the field, and healthcare professionals with lived experience of UB.

Stakeholder feedback was incorporated using the following process: (1) documenting theory presentation to stakeholders for refinement; (2) documenting suggested alterations; (3) performing purposive searching to sense-check non-aligned suggestions; (4) discussing discrepancies within the team to determine consensus and action taken; (5) re-presenting changes made to stakeholders/group for further sense-checking (e.g. using “you said, we did” summaries at start of each stakeholder group meeting). Key points of stakeholder feedback that we incorporated, and an example of our notes of stakeholder meetings with team annotation, are reported in Additional File 5.

When comparing against the ACTIVE (Authors and Consumers Together Impacting on eVidencE) framework for reporting stakeholder involvement in systematic reviews, this research adhered to a continuous, multiple-time, closed event approach, in which stakeholders were able to influence the results of the review [51].

### Changes to methodology in the study protocol

Since publication of our study protocol, there have been no significant changes, other than renaming causes to contributors to reflect that these relationships are often not fully deterministic [43]. Where flexibility was built into our protocol (e.g., with our relevancy criteria), the reporting of methods in this paper has been updated to reflect the final criteria we drew upon in the selection of documents.

## Results

### Document selection

**Fig. 3:**
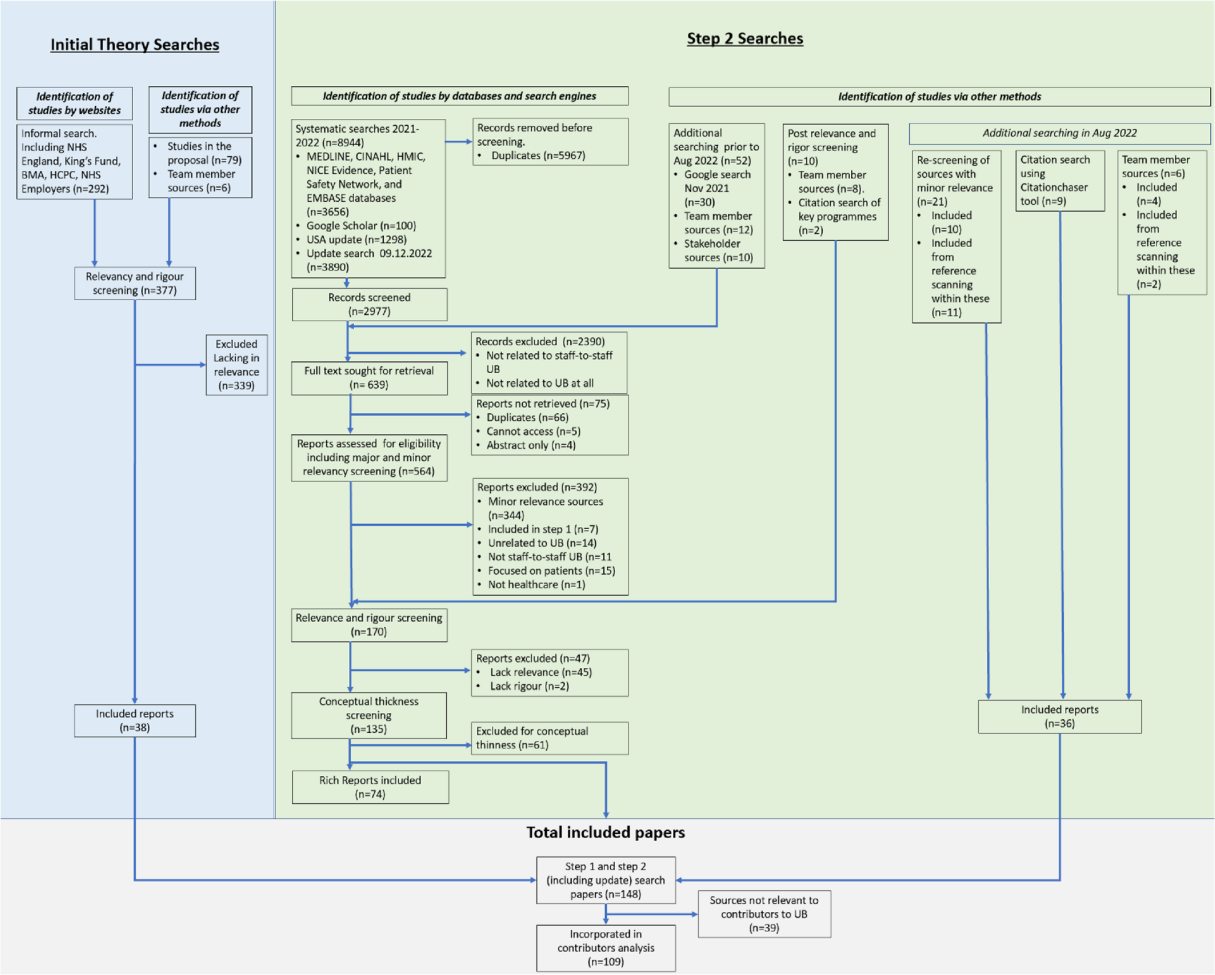
PRISMA style diagram reflecting the document selection process

We included 38 reports in Step 1 [2, 9, 14, 15, 17–20, 22, 24–28, 52–75]. Step 2 searches identified 8,944 records, reducing to 2,977 after de-duplication (*n* = 5,967). We added reports identified via Google, team members, and stakeholders (*n* = 62). In August 2022, we performed additional searching to identify further interventional literature for the wider project, which resulted in 36 reports being added. After application of inclusion and exclusion criteria, full text and conceptual richness screening, and relevancy and rigour screening, 148 reports were included. We then further screened reports for information relevant to the contributors to UB. In total, we included 28 reports for initial theory building [2, 14, 15, 17–20, 24–26, 28, 52, 55–57, 61–65, 67–70, 72–75] and 81 for theory refinement [11–13, 23, 30, 31, 70, 76–151], comprising 109 reports. Figure 3 depicts the complete document selection process.

The majority of reports used to understand contributors reported on an intervention (*n=*26), editorials (*n=*24), or were qualitative studies (*n=*16). In terms of healthcare type, most reports referred to healthcare in general or did not specify the context (i.e., were ‘general healthcare, *n=*50) or acute care (*n=*41). The primary unprofessional behaviour that studies focused on was bullying (*n=*30), followed by lateral or horizontal violence (*n=*14) and unprofessionalism in the form of communication issues (other forms of UB, *n=*12) and incivility (*n=*12). Lastly, for geographical region, most reports focused on the UK (*n=*29), no particular country (*n=*28) or the USA (*n=*28). Full details on these included reports can be found in Additional File 4.

### How do contributors to unprofessional behaviours work and why?

**Fig. 4:**
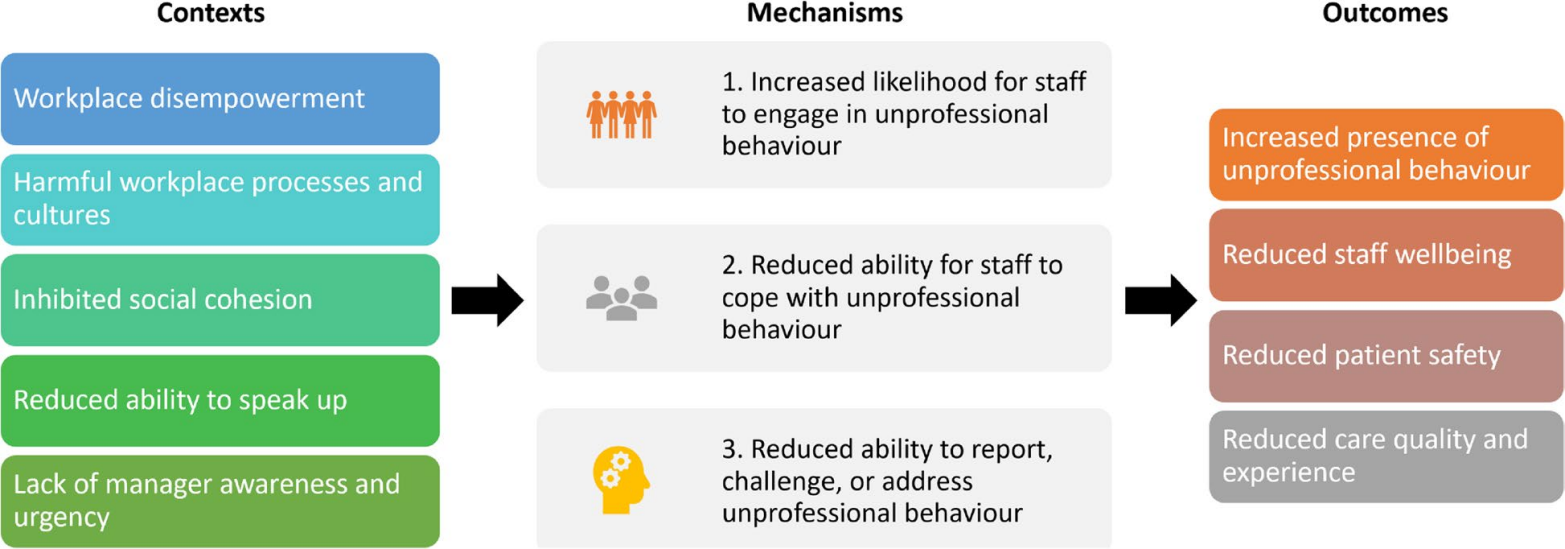
Overarching, high-level programme theory

This section initially explores how contributors worsen UB, before identifying five main types of contributor and programme theories for how and why these work. The five main categories of contributor that we identified included those that lead to: (1) workplace disempowerment, (2) enablement of harmful cultures and workplace processes, (3) inhibited social cohesion, (4) reduced ability to speak up, and (5) lack of manager awareness and urgency (Fig. 4). These are summarised in tabular form in Additional File 6.

### How contributors worsen unprofessional behaviour

Our analysis of contributors, which led to the development of our programme theories, identified three main mechanisms that worsened UB and its impacts including: (1) an increased propensity to engage in UB, (2) reduced ability to cope with the effects of UB, and (3) reduced ability to report or challenge UB. These allow UB to continue, as a reduced ability to cope worsens the impacts of UB on staff, UB becomes less frequently challenged, and managers may not be aware of where UB is taking place (Fig. 4).

**Fig. 5:**
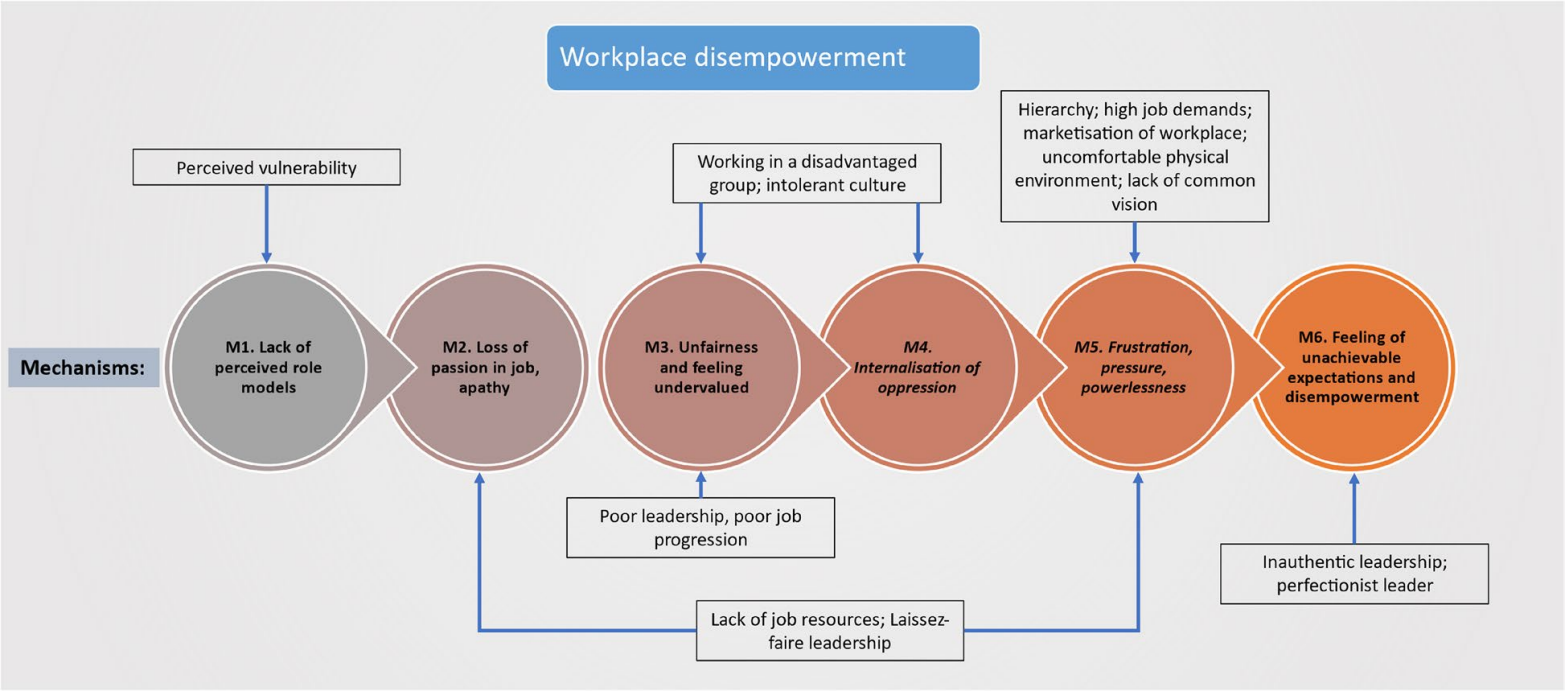
The process of workplace disempowerment (mechanisms 1 to 6). Italicised mechanisms are those that can directly increase incidence of UB. Boxed items are individual contributors

**Fig. 6:**
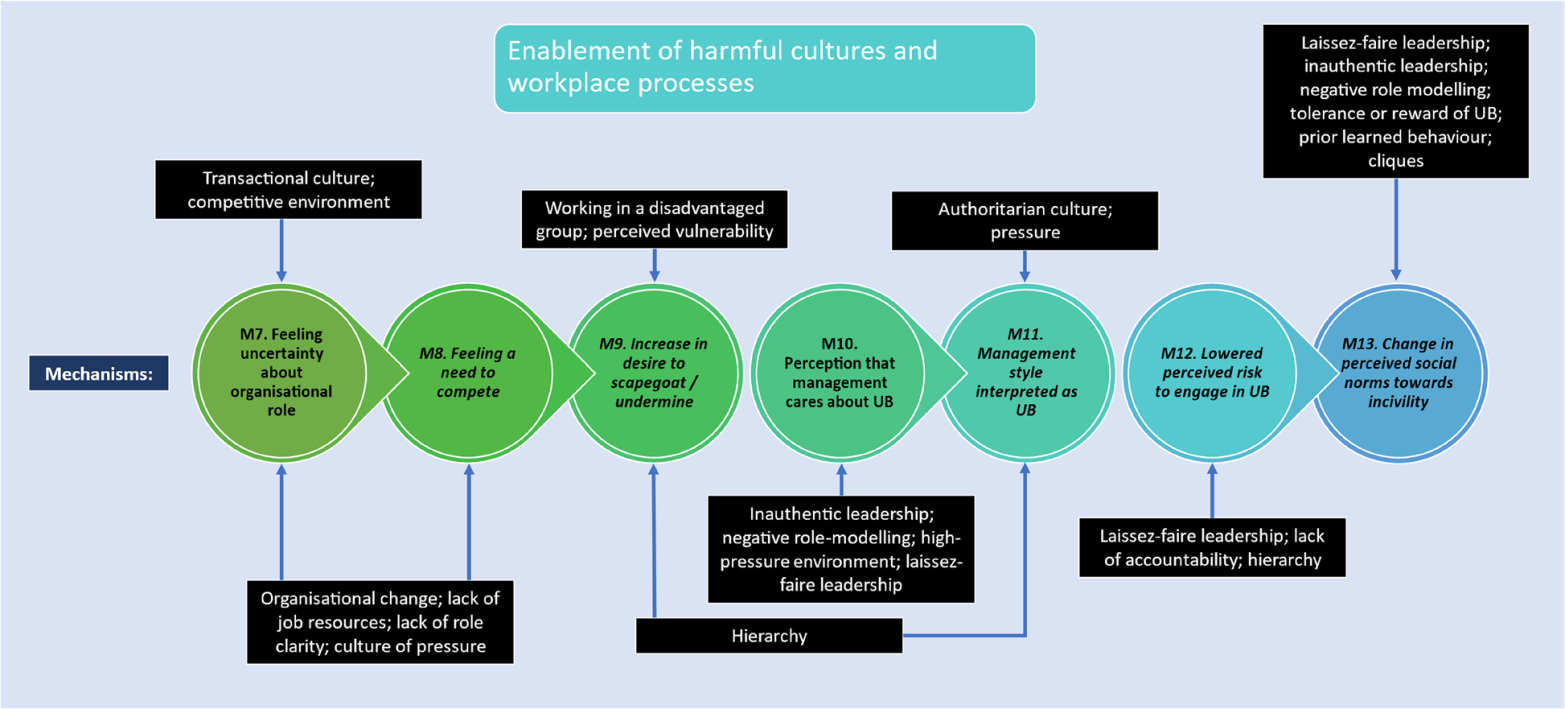
Programme theory for how harmful work processes and cultures work (mechanisms 7 to 13). Italicised mechanisms are those that can directly increase incidence of UB

**Fig. 7:**
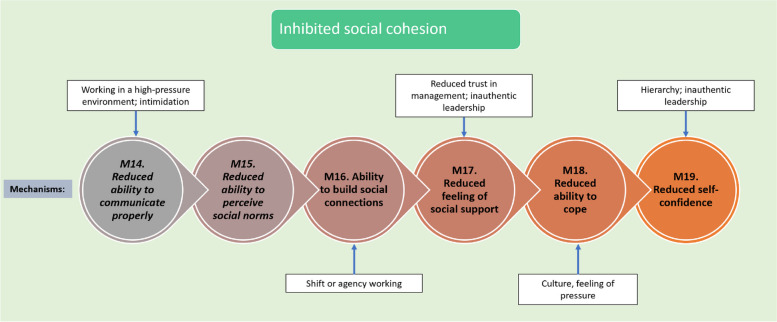
Programme theory for how inhibition of social cohesion works (mechanisms 14 to 19). Italicised mechanisms are those that can directly increase incidence of UB

**Fig. 8:**
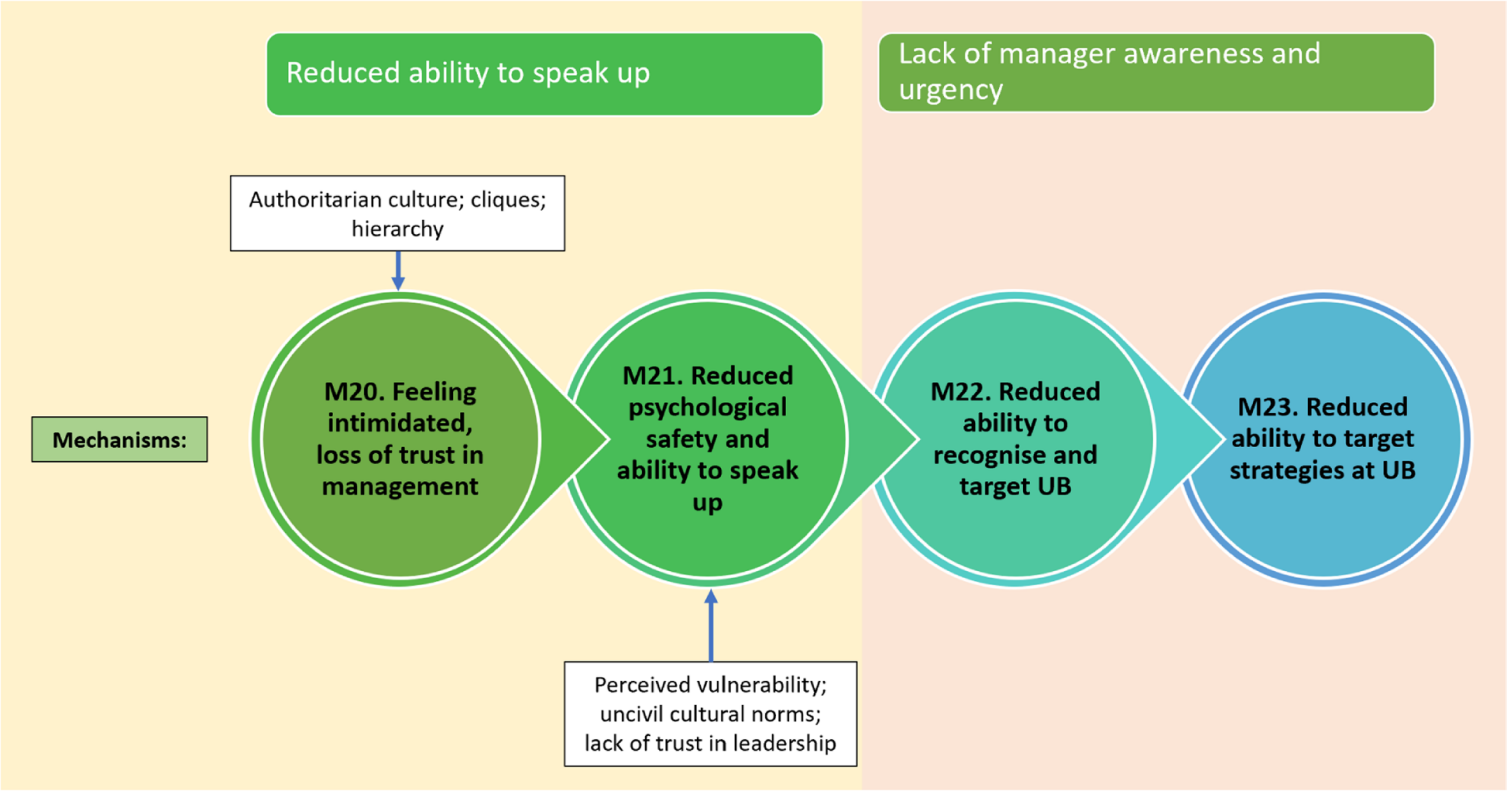
Programme theory for how reduced ability to speak up and lack of manager awareness and urgency work (mechanisms 20 to 23)

**Fig. 9:**
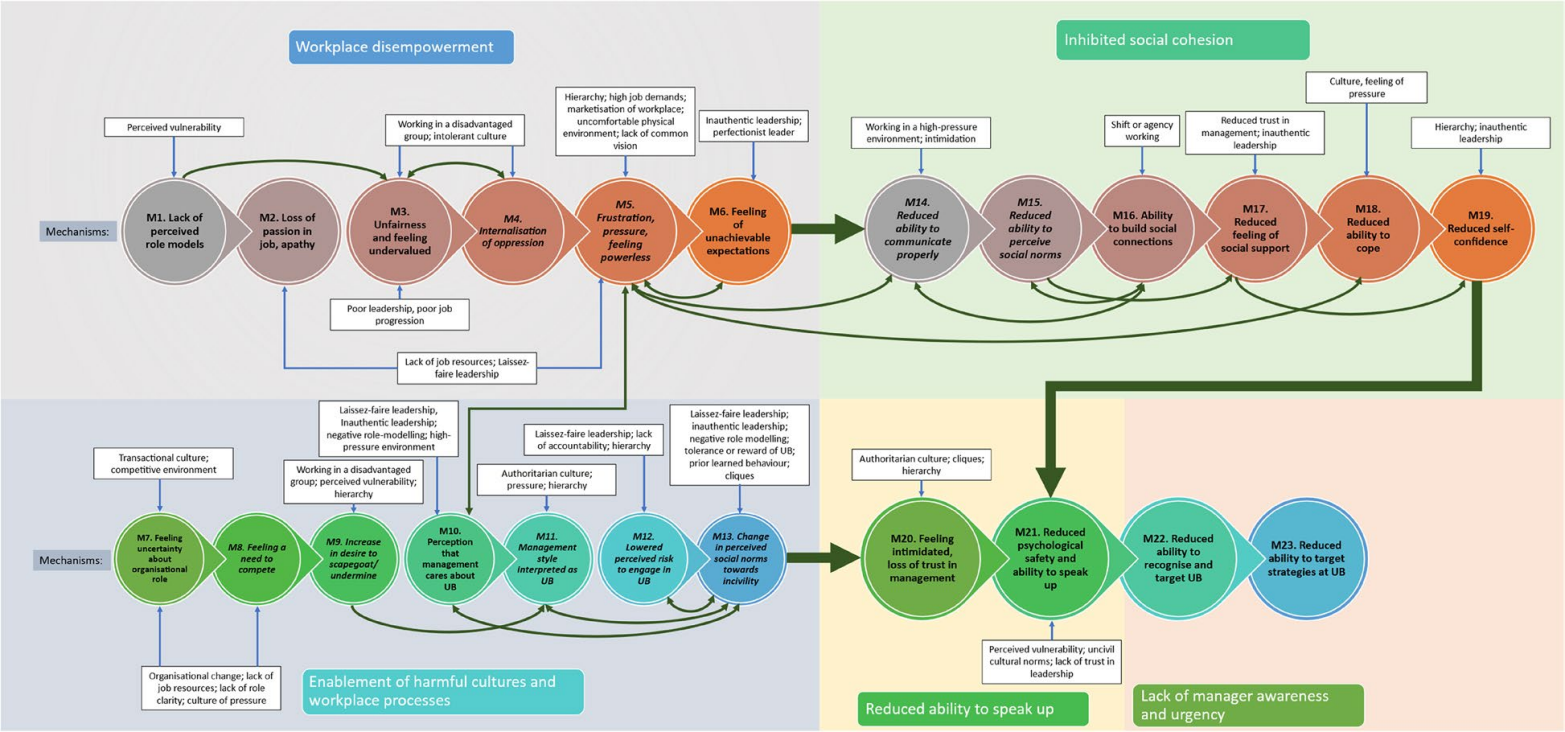
Programme theory depicting interactions of contributors and the mechanisms they trigger. Arrows depict interactions and vicious cycles between mechanisms (double ended arrows)

This section discusses the five major categories of contributor (or context in which UB is more likely to occur) outlined above, presenting a simplified programme theory diagram for each category in Figs. 5, 6, 7 and 8 (note, in Fig. 8, contributor categories 4 and 5 above are combined, due to there being fewer mechanisms to portray). These diagrams depict more information than is discussed in the text and they are not intended to suggest that the relationships between mechanisms are linear. How these categories of contributor interact in their full complexity are then discussed and depicted in Fig. 9. Mechanisms are consistently numbered throughout this paper (in both the diagrams and in text and CMOCs) for consistency and clarity.

#### Context 1: Workplace disempowerment

Multiple factors can create workplace disempowerment including (1) organisational hierarchies; (2) unfairness; and (3) the physical environment [105, 125, 146, 151].

Organisational hierarchies can exist both within and between professions. An interprofessional hierarchy is exemplified by the relationship between doctors and nurses, whereby doctors are often considered to be in a position of power relative to nurses [115, 146]. Intraprofessional hierarchies also exist and can be between a nurse and a nurse manager, or, for example, between more entrenched/experienced and newer staff. Hierarchy can be a result of a socially constructed environment where certain groups or individuals are perceived as more ‘powerful’ than others, or can be a result of the design of wider society in which organisations operate [114] (e.g. gender, cultural, or age-based hierarchies) [90]. Hierarchies can have multiple effects, such as making it more difficult to speak up. One source exemplified how hierarchies can lead to a culture of blame and intimidation:

*“There appeared to be a style of management within nursing at this hospital that was based on fear rather than respect. There was an impression that nurses were tolerated rather than valued, that they should keep their heads down and not threaten those above them by disagreeing with them.”* [116]*.*

**Table:** 

**CMOC 1.** If staff work in a disempowered position, such as at the bottom of a hierarchy (C), then this can increase the likelihood of experiencing, exhibiting or being impacted by UB (O) because it can make staff an easier target (M12).	

Being in a disempowered position can often lead to feelings of frustration with one’s position, and studies reported that this could result in displacement of these frustrations onto others in the workplace [132].

**Table:** 

**CMOC 2.** If staff work in a disadvantaged group (C) then this can lead to displacement of aggression to others (O1) and a feeling of being undervalued (O2) due to internalisation of oppression (M4).	

Being in a lower hierarchical position can make staff feel disempowered and their organization is unjust. A feeling of injustice or unfairness can also arise as a result of unfair processes, for example, “*bestowing apparent favours on some doctors in training by giving them access to resources, such as study leave or training opportunities, while denying these to others*” [67]. Such inequity can be considered discrimination, or ostracisation, in itself. Over time, this can lead to a sense of annoyance and frustration that can eventually lead to conflict [24]. This is highlighted in CMOC 3 below.

**Table:** 

**CMOC 3**. If staff work in a disempowered position where there does not seem to be a level playing field (C1) or work in a physically uncomfortable environment (C2), then this can cause people to externalise these frustrations, increasing proclivity to engage in UB (O2) because staff feel like they are being treated unfairly (M3) experience frustration (M5) and have a reduced ability to cope (M18/O1)	

Physically uncomfortable environments are common in healthcare workplaces, e.g., where it is too hot or crowded, or in close proximity to disease (such as during the COVID-19 pandemic) and even death. Working in such environments can increase a sense of pressure, frustration, and reduce ability to cope [24] (see CMOC 3 above).

Figure 5 below depicts the process of workplace disempowerment. Certain elements which have not been discussed here also feature, including leadership behaviours. These will be discussed in a later section to keep this discussion more concise.

#### Context 2: Harmful workplace processes and cultures

Harmful workplace processes can cause challenges in building relationships and increase frustrations, which, in turn, increases conflict. Harmful workplace processes include: (1) organisational change; (2) a lack of resources and high job demands; (3) a culture of pressure and; (4) a lack of role clarity. Harmful work cultures can also create an environment permissive of UB, and leadership is often a reason why such cultures manifest. A harmful culture can be enabled by: (5) complicit and permissive leadership; (6) negative role modelling and prior learned behaviour; (7) an authoritarian culture a; (8) lack of organisational accountability; (9) organisational deafness; and (10) cliques. The dynamics discussed in this section are depicted in Fig. 6.

Organisational change can increase UB if managed poorly. Reports suggest an increase in uncertainty about one’s organisational role and/or an increase in workload, as well as potential job insecurity [144], may result in an increase in competitive attitudes that can set employees against each other. This increase in competitive attitudes can further reduce the ability to engage in teamwork and generate conflict and UB in an organization (CMOC 4). For example:

*“In competitive environments, organizational re- structure or periods of rapid change may create opportunities for individuals to engage in the misuse of legitimate authority for furthering self-interest or career opportunities”* [147]*.*

**Table:** 

**CMOC 4**. If staff experience a period of organisational uncertainty, such as organisational change (C) or they experience a lack of job resources (C2), then this can lead to conflict and UB (O) because staff perceive their job is at risk and an increase in competitive attitudes ensues (M8).	

Demanding work environments with high job demands and a lack of resources were also identified as contributors to UB. These worked by reducing the ability to communicate effectively with colleagues, which could then increase the chance of communications being perceived as UB. For example, one source highlighted that:

*“… consultants told us they were more likely to speak sharply to doctors in training when they themselves were stressed. They reported that they were less able to prioritise the training needs of their juniors, and less likely to treat them with respect at all times“* [67]*.*

**Table:** 

**CMOC 5.** If high job demands are experienced regularly in the absence of adequate resources (C1) then escalation of conflicts are more likely (O2), because there is both a high-pressure environment (M5) and reduced ability to communicate effectively (M14/O1)	

This dynamic is explored further with the culture of pressure section below.

Job demands can also include the complexities of healthcare work and organisational bureaucracy. Opaque organisational processes (such as not knowing whose responsibility it is to do a certain task) were found to needlessly add to frustration and drive conflict, highlighted in the following example:

*“When incompatible social structures bear on a situation, the clash creates unresolvable conflicts fuelled by the simultaneous compulsion to fulfil irreconcilably oppositional obligations, creating a downward spiral of communication that circles back to escalate the conflicts“* [87]*.*

A lack of job resources can include financial, human, and clinical resources, all of which contribute to an inability to adequately perform one’s role. Worldwide staff shortages worsen this issue. One paper highlighted the impact of austerity in the UK on job resources, highlighting that:

*“… tightening regulation and oversight requires staff to provide efficient, high quality and safe care despite growing material and staff scarcity, increasingly complex workloads, worsening pay morale and development prospects, and a pressurized, high stakes environment. Unsurprisingly, this can undermine cultures of solidarity and respect between frontline professionals”* [80]*.*

These dynamics tie in with CMOC 5 (above) but can also lead to a culture of pressure (CMOC 6). Significant job demands or a lack of resources can contribute to a sense of a pervasive culture of pressure which can make it difficult to meet expectations and complete work to a high standard, increasing stress. This manifests in a feeling that one is not in control, which can generate a sense of frustration. In some cases, frustration can be externalised as a coping mechanism. One report highlighted this dynamic:

“… *nurses commit negative workplace behaviours in order to release the feelings of frustration and regain a sense of control*” [114].

**Table:** 

**CMOC 6.** If staff experience a lack of resources, or high job demands that increase pressure (C) then this may lead to an inability to cope with the impact of UB, (M18/O1), because communication with co-workers can be inhibited (M14) meaning it is difficult to build relationships with colleagues (M16) which can reduce feeling of social support (M17)	

A lack of role clarity can result in a situation in which the lines between staff members’ responsibilities, and those of others, are blurred, and staff can find themselves in unnecessary conflict through no fault of their own (CMOC 7).

**Table:** 

**CMOC 7.** If staff are disadvantaged by organisational processes outside of their control, such as a lack of role clarity or high job demands (C) then this may increase levels of curtness in communication (O2) because they begin to feel pressured and their tasks become rushed (M5), reducing ability to communicate effectively (M14/O1)	

Our findings indicate that enablement of harmful work cultures generally requires the permission of complicit or permissive leaders to persist. Highlighting this are the many examples in which ‘high-performers’ are promoted or allowed to continue work without reprisal, despite it being known that they engage in UB:

*“‘The work environment is toxic with male surgeons who bring in high revenue streams to the hospital (and) seem to be allowed to treat staff as they please despite the fact that staff have raised concerns.’”* [12]*.*

This can result in staff learning that UB is acceptable or even necessary to succeed, causing such behaviours to be replicated (CMOC 8). Laissez-faire (or permissive) leadership is a related scenario which can lead to a situation where UB is allowed to persist through avoidance or negligence. This can signal that there would be no consequences for potential instigators of UB [144, 146] (CMOC 11). Negative role modelling and prior learned behaviour within the workplace, particularly by senior staff, can also give the impression that management tacitly support UB [99], as reflected in CMOC 8:

**Table:** 

**CMOC 8.** If a workplace has a prevalence of UB and leaders/ managers are not seen to address it by being complicit or laissez-faire (C1), then this can cause staff to engage in UB (O) and reduce trust in leadership (O2), because UB is perceived as accepted and normalised (M13)	

In some cases, negative role modelling can create learned behaviours, e.g., where “*nursing students can be bullied by one another and by faculty in both face-to-face and online learning environments*” [125]. This can lead students or staff moving organisations and bringing practices with them into the workplace that can propagate a bullying culture [77] (CMOC 9):

**Table:** 

**CMOC 9.** If a workplace has a high prevalence of UB and leaders or managers are not seen to address it (C) or role model it themselves (C2) then there is an increasing likelihood of others engaging in UB (O) and a loss of trust in leadership (O2) because the impression can be given that engaging in incivility and other UBs is the norm (M13) which reduces perceived risk for instigators (M12), and ability to speak up for victims (M21)	

An authoritarian leadership style can further foster an environment in which UB can thrive. This may happen in high-pressure environments, such as surgical departments, in which “*professional stressors related to surgical processes and procedures appear to activate or elicit authoritarian and hierarchical modes of interacting between inter-professional groups”* [12]. Such a leadership style can lead to development of an authoritarian culture, which can also inhibit psychological safety and thus ability to speak up, resulting in the continuation of UB.

When people speak up but do not feel heard, and no action is taken, this can also be referred to as organisational deafness [68]. Jones & Kelly (2014) highlight that, in many cases, employees who do try to speak up and indicate that there is a problem, receive no acknowledgement or response from their managers or the organisation (a deaf effect) [68]. The signal this sends to staff cannot be underestimated, and the damage to trust between employees and their leaders may be as damaging as the UB itself. This places an onus on organisations to be actively listening and acting on concerns to tackle UB as it arises. If leaders are seen to listen but not to act, then this can also be interpreted as a lack of accountability, which is reflected in CMOC 10 below.

**Table:** 

**CMOC 10.** If UB is prevalent in a workplace and managers are not seen to address it (i.e. lack accountability) (C1) or even negatively role model such behaviours themselves (C2) then this can reduce psychological safety (M21/O1) and reduce trust in management (O2) because staff sense that the organisation is deaf (i.e. that they do not care about UB and do not act upon reporting of UB) (M10) which creates an intimidating culture (M20)	

#### Context 3: Inhibited social cohesion 

Inhibited social cohesion can increase the likelihood of UB occurring, leading to a reduction in staff members’ ability to cope with UB. This can occur due to factors discussed previously, including: (1) a lack of social support, (2) shift or agency working, and (3) ability to communicate effectively. How these factors contribute to UB are depicted in Fig. 7.

A lack of social support can be a result of other contributors outlined earlier, such as a culture of pressure undermining an ability to build relationships or being at the bottom of an organisational hierarchy. One study stated that when social support exists:

*“… rallying around a victim in solidarity demonstrates a united front against the bully and is a means of taking power away, thus deflating the perceived outcomes from the bullying”* [83]*.*

A lack of social support causes a reduced sense of self-confidence, which also inhibits speaking up. This will be discussed further in the next section.

A move to shift and agency working can make it more difficult to build relationships with colleagues. This may reduce ability to communicate effectively and can make staff feel unsupported [61]. The consequences of this, in one case led:

*“…to staff being unable to build a sense of team collegiality ‘because I now work with so many different people – no-one has my back anymore’”* [144]*.*

This was highlighted as inhibiting an ability to build social connections and to cope, because: “’*I can’t confide to my manager because I never see them and now I can’t confide with my mate because I don’t know who my mate is’*” [144].

**Table:** 

**CMOC 11.** If staff work in shifts (C1) and/or lack social support (C2), then this can reduce the ability to cope when experiencing UB or workplace stressors (M18/O1) and reduce self-confidence (M19/O2) which can worsen the impact of UB on health and wellbeing (O3) because these can reduce ability to build social connections (M16) and lessen feeling that one is socially supported (M17).	

The ability to communicate effectively is also crucial for maintaining a civil work environment and for maintaining clinical quality. As highlighted earlier, several factors can reduce the ability to communicate effectively, including a culture of pressure or intimidation, and high job demands. Thus, an inability to communicate effectively can cause vicious cycles to increase the impact of UB on staff, and directly exacerbate conflicts. This is highlighted in the following quote in which a cycle between communication and frustration is outlined: “*a lack of communication between the physician and the nurse can result in stress for the nurse […], and conflict between nurses and physicians and between nurses results in feelings of anger and frustration* “ [83]. Reports highlighted that “s*imple gestures such as open, honest, transparent communication go a long way to build rapport with workers*” [145]. This is reflected in CMOC 12.

**Table:** 

**CMOC 12.** If staff work in a high-pressure environment or in a culture of intimidation (C) then this can lead to reduced ability to build social connections (M16/O1), a reduced ability to determine social norms (M15/O2), and a reduced sense of social support (M17/O3) because there is a reduced ability to communicate effectively (M14).	

#### Context 4: Reduced ability to speak up 

Informal alliances or cliques can create an environment in which UB is tolerated or even encouraged by local line managers, with one study stating “*a tolerance of bullying behaviour formed because of the power of these alliances. 'They were really a strong force, really opposing anything different. And, they were (…) fairly united and stuck together’*” [19]. Reports suggested that cliques can manifest at any hierarchical level and can operate to undermine and minimise challenge and speaking out by weaponizing social ostracization and intimidation tactics [76].

In addition to cliques, many of the prior factors discussed here, including all forms of workplace disempowerment (e.g., hierarchies), harmful work cultures, and social inhibition, can lead to reduced psychological safety. These are reflected in CMOC 13 and depicted in Fig. 8.

**Table:** 

**CMOC 13.** If staff work in a disempowered position such as at the bottom of an organisational or professional hierarchy (C1) or within a harmful organisational culture (C2) or work in an environment with exposure to negative cliques (C2) then this can inhibit willingness to speak up (M21/O1) and reduce ability to communicate (M14/O2) because staff experience a sense of intimidation and reduced psychological safety (M20).	

#### Context 5: Lack of manager awareness and urgency

A vicious cycle can be created when a culture inhibits ability to speak up, which can in turn mean managers do not know that there is a problem with UB, which means that the culture is not addressed. This dynamic is reflected in CMOC 14. How both a reduced ability to speak up and lack of manager awareness and urgency contribute to UB are depicted in Fig. 8.

**Table:** 

**CMOC 14.** If a reduced sense of psychological safety leads people to not speak up (C) then strategies to address UB are not able to be implemented (M23/O1), reducing trust in leadership (O2) because managers are not aware that UB is taking place (M22).	

### How do contributors to unprofessional behaviours interact?

Our overarching programme theory combining all contributors, presented in Fig. 9, depicts the categories of contributors (labels in rectangles), the sub-contributors (white boxes), and the mechanisms (circles) and interactions between them (blue arrows for contributors to mechanisms, and green for mechanisms to mechanisms). Each of the 5 major contributors discussed and presented above are represented in the 5 rectangle sections in the diagram.

The simplified linear depictions in Figs. 5, 6, 7 and 8 are augmented in Fig. 9 by the addition of mechanism-to-mechanism interactions. This depiction represents the interconnections between programme theories discussed in the sections above. Outcomes of certain CMOCs become contexts for others creating “ripple effects” [152] or causal chains. For example, a lack of job resources (C) can lead to frustration and a feeling of powerlessness (M5) which can then lead to reduced ability to cope (O/M18). Likewise, an authoritarian culture (C) can lead to a management style being interpreted as UB (M11) leading to a change in social norms towards incivility (O/M13). This can then lead to concomitant effects because the change in organisational norm towards incivility (C) can create an atmosphere of intimidation (M20) which can reduce sense of psychological safety to speak up (O/M21). These dynamics highlight how mechanisms for one CMOC can form the contexts or outcomes for another CMOC, leading to escalations.

### How do unprofessional behaviours impact patient safety?

**Fig. 10:**
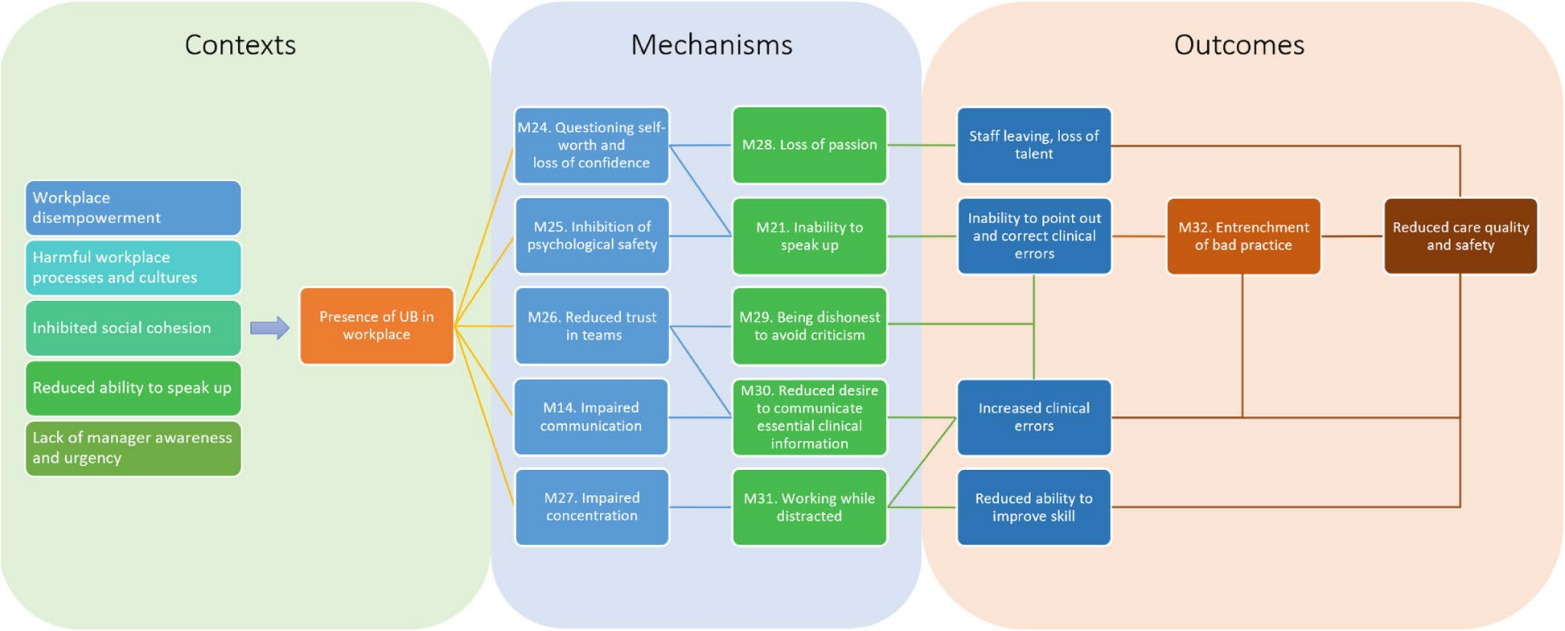
Programme theory for how UB leads to reduced care quality and safety. Previously presented CMOCs are numbered in the diagram. Intended to be read from left to right

The presence of unprofessional behaviour can significantly impact patient safety [2, 27]. Our analysis identified six main ways in which this could occur, namely: (1) a loss of confidence in one’s abilities, (2) reduced psychological safety inhibiting the ability to report medical errors, (3) reduced trust in teams reducing information sharing, (4) reduced communication overall, (5) impaired ability to concentrate and think, and (6) a culture that becomes accepting of mistakes. These factors could also interact with each other to increase the impact of UB. Programme theories and supportive quotes for these are outlined below. Since many of these relationships are explained in Fig. 10 below, these CMOCs are presented in Table 3 for brevity.

**Table 3:** Programme theories underpinning the impact of UB on patient safety

**Programme theories**	**Example illustrative data**
**CMOC 15. Loss of confidence** If unprofessional behaviours are present in a clinical workplace (C) then staff can experience a loss of passion for work (M28/O1) and desire to leave one’s organisation (O2) because UB causes staff to question their self-worth and experience a loss of confidence in one’s abilities (M24)	“… *this study suggests that incivility can cause a practitioner to question their actions and ability which can negatively impact on clinical decision- making. Participants suggested that they may ‘take the path of least resistance’*” [105]
**CMOC 16. Reduced psychological safety** If unprofessional behaviours are present in a clinical workplace (C) it can cause greater incidence of medical errors (O1) and therefore reduced care quality and safety (O2) because presence of UB can lead to reduced psychological safety (M25) which can inhibit ability to speak up when medical errors occur (M21)	“… *individuals who engaged with impunity in transgressive or disruptive behaviour—corrupted the conditions of a healthy working environment, resulting in personnel feeling fearful and lacking in psychological safety*” [104]
**CMOC 17. Reduced trust in teams** If unprofessional behaviours are present in a clinical workplace (C) it can lead to increased clinical errors (O1) and therefore reduced care quality and safety (O2) because UB can reduce trust in teams (M26) or be dishonest to avoid criticism or reprisal (M29) and reduce desire to communicate essential clinical information (M30)	“*Incivility caused participants to ruminate on their behaviour to try and find fault with themselves rather than question the negative behaviour of colleagues. A feeling of paranoia is generated, again reducing trust*” [13]
**CMOC 18. Impaired concentration and thinking** If unprofessional behaviours are present in a clinical workplace (C) it can lead to increased medical errors (O), and a reduced ability to improve one’s skill (O2), causing a reduction in patient safety and care quality (O3) because UB can impair concentration (M27) and cause staff to be distracted while working (M31)	“’*…you might be more focused on what’s just happened rather than on the patient themselves and you might miss something on an assessment or treatment [] your mind will not be on the job’*” [13]
**CMOC 19. Culture accepting of mistakes** If there is an unprofessional environment consistently rife with errors (C) then this can lead to further loss of care quality and safety (O1) because such an environment can cause entrenchment and normalisation of medical errors in the culture (M32)	“*This includes surgeons in difficulty blaming others, dismissive of concerns raised about them, and becoming entrenched in their position, sometimes becoming ‘controlling’ or ‘arrogant’ in their approach*” [112]

The interactions of these factors are complex, so to better depict them, we developed a programme theory diagram (Fig. 10). Within this, we have numbered the mechanisms outlined above. Some, such as M14 and M21, have already featured in previous sections. These highlight how certain contributors, such as hierarchy, may lead to inhibition of ability to speak up about medical errors, even if UB does not occur.

### How do unprofessional behaviours impact staff and their wellbeing?

Exposure to UB can detrimentally affect mental and emotional health of staff [37]. A recent review investigating factors influencing psychological distress among nurses, midwives, and paramedics revealed that working in an environment where staff members mistreat each other can lead to moral distress, ultimately contributing to increased stress levels and burnout [37]. Additional consequences of being exposed to UB include the development of conditions like post-traumatic stress disorder, anxiety, depression, and disruptions in sleep patterns [20]. Even bystanders and those who witness UB can undergo a corresponding decline in their own psychological well-being, often experiencing moral injury due to their inability to prevent such behavior [24]. In severe instances, staff who are subjected to UB have taken their own lives [22]. Moreover, other repercussions of UB, such as a decrease in self-confidence, can have a cascading effect, further diminishing the affected individuals' capacity to handle UB. CMOC 20 reflects these findings.

**Table:** 

**CMOC 20. Impact to staff wellbeing** If staff experience UB which they consider severe or prolonged (C) then they can experience post-traumatic stress, anxiety, depression, or burnout (O) that, if unaddressed, can lead to them taking sick leave, leaving an organisation, or even having suicidal thoughts (O2). This is because staff can become disempowered and lose self-confidence (M24), experience prolonged negative emotions (M33) and social isolation (M17) and can become fearful of coming to work in such conditions (M34)	

### Who is most affected by UB?

We identified that UB in the healthcare workplace affects particular groups more than others. Individual responses to UB are often complex and dependent upon several factors including the type of UB experienced, individual circumstances, and interactions with factors already explored in this review, such as a lack of social support [12]. This means that not everyone will experience UB in the same way. Recent research suggests that vulnerability to UB (i.e. likelihood of negative impact to wellbeing) among doctors increases with greater exposure to UB, being female, and under 55 years of age [32]. The groups most affected by UB, and why this may be the case, are outlined in this section. CMOCs outlined here draw on new mechanisms as well as mechanisms explained and numbered across prior sections.

#### Staff from minoritised groups and women (e.g., ability, race, gender, religion)

Staff can be discriminated against based on age, gender, race, religion, sexual orientation, relationship status, country of origin, disability, or pregnancy. Groups of staff with such characteristics were more likely to be targets of UB in the form of harassment, discrimination, and microaggressions [119, 148]. Literature highlighted that LGBTQ + staff were second-most likely to experience UB in the healthcare workplace after staff with a disability [65]. Staff with a disability were found to more frequently experience forms of ableism from managers, often stemming from a sense that managers were not sufficiently accommodating of their needs in the workplace [75]. Staff from overseas were also found to encounter difficulties. Speaking English less well than others or with an accent was found to affect peer relationships [110] and could cause staff to feel marginalised. For example, one interviewee reflected that “*…in my earlier job when I was leading the team people will come… and will meet me sitting down and they will bypass me and go to white staff”* [110]*.*

Exposure to UB amongst marginalised groups may be affected by harmful cultures that reflect wider societal attitudes; including issues such as structural racism and patriarchy [110], as well as interactions of other dynamics such as hierarchies, such as between nurses and doctors. As such, societal attitudes provide the wider context in which UB in healthcare organisations occurs. Agency staff and staff from marginalised backgrounds are more likely to work in shifts. This has manifested in a number of ways. For example, during COVID-19, in the UK, 47% of minority staff worked in pandemic-specific roles, compared to 31% of all staff [79].

Microaggressions were also highlighted as an insidious form of UB that have significant impact over time. Microaggressions have been defined as “*stunning and automatic acts of disrespect arising from unconscious attitudes inflicted by the culturally dominant groups*” [110]. Microaggressions were found to silence recipients and making them feel disregarded, as well as reduce their job performance, contributing to poor psychological health [63]. This indicates microaggressions are no less serious than other forms of UB [119, 148]. A review of culture at an NHS trust by Kline (2022) highlighted that few people could define microaggressions or understood their impact [151].

Interventions to address UB towards minoritised staff groups are almost non-existent [3, 35], which may be due to stigma around acknowledging racism or sexism. For example, the Kline report on culture at a UK NHS hospital found that a harmful culture in which senior leaders “*won’t say anything because they’re afraid of being called racist*” led to inhibition of strategies being implemented to address the issue [151]. These factors are represented in CMOC 21.

**Table:** 

**CMOC 21. Outcomes for staff with protected characteristics.** Staff from minoritised groups, and women (C) are more likely to experience UB from other staff members (O) because they are at higher risk of being in disempowered positions in the workplace (M35), may be less likely to receive social support from colleagues (M17), and because stigma can prevent structural issues (racism, sexism, ableism, or transphobia) being acknowledged or addressed (M37).	

#### Students and those new to the profession

Newly qualified staff and students were highlighted within the literature as more likely to be on the receiving end of UB. Students and new staff are often at the bottom of the workplace hierarchy and are unsure about social norms in their workplace [109, 133]. Reports referred to both medical students and graduate nurses as more prone to the impacts of UB. For example, one study identified that 31% of newly graduated nursing students reported experiencing bullying [149]. This could lead nurses, particularly those from ethnic minority backgrounds, to leaving the workforce at an early career stage and/or completely changing career [94]. Included literature reported that graduate nurses experienced UB more frequently, often in tandem with exclusionary behaviours such being dismissed and ostracised [30].

Graduate nurses and students are reliant on their relationships with mentors and supervisors and trust in their supervisor is key. This must also be balanced with a feeling that they are being useful and contributing to their work environment [146]. The impact of high job demands can most negatively impact those new to the profession, reducing time interacting with more experienced colleagues [129]. Seeking interaction with mentors and supervisors means that if mentors and supervisors model UB-promoting behaviours, graduate students are at greater risk of internalising and potentially subsequently reproducing UB (and perceiving such behaviour as normal) [150]. CMOC 22 highlights this dynamic and how the experience of new entrants to an organisation can be underpinned by inhibited social cohesion (context 3 above).

**Table:** 

**CMOC 22. Experience of UB by students and new graduates.** Staff such as students and others new to the healthcare profession (C) can experience more UB than other groups (O) because they are initially lacking self-confidence (M19), are relatively disempowered due to hierarchy (M5), are socially vulnerable due to seeking to integrate into the social environment and be accepted (M16-17); may have less concept of the pervading social norms (M15) and may be perceived by others as less capable (M36).	

#### Inter- and intra-professional UB

Overall evidence regarding interprofessional UB and its frequency was mixed [14]. However, surgical contexts were consistently reported as ‘hot-zones’ for UB, which perhaps relates both to a greater and more rigid hierarchy, gender dynamics, and the high-pressure and high risk work environment [112]. The presence of a steep hierarchy was often cited as inhibiting effective communication (reducing psychological safety), and impacting patient safety and care quality [130]. A study across seven Australian hospitals in 2022 highlighted that the environment in surgery is an ongoing problem, with one participant stating “*Some (surgical medical staff) in theatres are still very rude to nurses—every week I would witness a surgeon raising his voice, yelling, throwing things out of anger and impatience…*” [12]. A recent UK study also highlighted the high prevalence of sexual harassment and assault of female surgeons by male surgeons [153]. Indeed, these intraprofessional forms of UB were consistently reported as more damaging to wellbeing. For example, nurses reported that UB originating from other nurses was more hurtful than negative behaviours originating with other professional groups, indicating that intra-professional UB may be perceived as more psychologically damaging [83]. This might be because staff desire closer relations with coworkers in the same professional group, and at their hierarchical level and in their team. See CMOC 23.

**Table:** 

**CMOC 23. Inter- and intra-professional UB.** If UB is experienced at an intra-professional level (C), then it can be perceived as more harmful (O) because it can feel like more of a betrayal (from within own profession) when horizontal violence occurs (M36) and can have a greater impact on interpersonal relationships that are more frequent and meaningful (M37)	

### Individual factors as contributors to unprofessional behaviour

The literature emphasises individual and personal characteristics as contributors to unprofessional behaviours, or as attributes of targets of UB that increase vulnerability. For example, one report discussed ‘maladaptive personality traits’ such as being “*paranoid, narcissistic, passive- aggressive and borderline types*” as being causes of UB. It also described instigators of UB as having, “*poorly controlled anger”* or perhaps experiencing a “*spillover of home problems*” [112]. These individuals who are perceived to have a greater proclivity to engage in UB than others are often referred to as ‘bad apples’ in the literature [56]. Literature also suggests that staff who may experience UB are simply not resilient enough [114], or are, in some cases, interpreting behaviours as UB that others may consider to be benign [105]. However, suggestions by organisations that UB will always take place and that staff should just be ‘more resilient’ can inadvertently send the message that UB is tolerated.

While certain individuals may have a predisposition to engage in UB, or may be more susceptible to organisational contributors, these personalities are not predictable or readily modifiable outside of recruitment processes and thus are not generally targetable by interventions. Furthermore, discrimination by employers based on perceived personality types or traits might be unlawful in certain countries, especially if staff have not actually behaved poorly. Still, in many interventions, organisations often try to identify these ‘bad apples’ based on behavioural patterns to address their behaviour through disciplinary proceedings in the absence of any system-level interventions [3, 35].

Some literature argues that by focusing on individual characteristics as contributors of UB, organisations can use this as a ‘get out of jail free’ card to enable abrogation of responsibility and accountability for enabling wider cultural change interventions targeting UB [99, 154, 155]. Moreover, evidence regarding individual-level contributors such as personality types, gender, or professional group, is often very mixed and purely based on associations, meaning it is impossible to identify potential instigators based on any particular characteristic [14]. The focus on ‘bad apples’, or groups of staff (e.g., surgeons or doctors), places blame on people who exist within teams and organisations that often have many unaddressed systemic contributors to UB that can be more readily targeted by interventions.

As such, it is likely to be more productive for organisations to focus on modifiable factors, targetable by interventions, to reduce UB. Such interventions could include a focus on improving working conditions, improving climate and culture, fostering processes to speak up and be heard, and eliminating barriers to providing high quality care [3, 35]. In this manner, an organisation can create an environment that is intolerant of UB, regardless of the characteristics of the individual staff working at their organisation. Mannion et al. (2019) referred to this as addressing problems at the level of bad cellars (organisations), bad barrels (health systems) and bad orchards (professions) rather than the usual ‘bad apples’ [56]. Professional accountability programmes, such as Ethos in Australia, and Vanderbilt in the USA, show promise in fostering this kind of culture change [35, 136, 156].

## Discussion

This is the first realist review to draw on a comprehensive understanding of contributors to a wide range of UB types in acute care. A realist review published in 2013 focused on bullying in healthcare and explored its antecedents [24], but did not include wider forms of UB, including incivility, microaggressions, etc. In a systematic review from 2020 on this topic, situational factors such as workload, communication, and teamwork have also been highlighted as core triggers of UB [14]. While these prior reviews identified many of the same contributors identified in the present review, we have developed a much more comprehensive programme theory to describe how, why, and in what circumstances UB occurs and whom it most impacts. We found that key contributors to UB are workplace processes that promote frustration such as understaffing and bureaucracy, cultures which perpetuate and tolerate UB (often stemming from leadership), and issues such as shift working. Some of these factors such as shift working have led to staff experiencing worsened rapport with their colleagues. These contributors come together to collectively constrain the ability for staff to speak up and challenge UB, further inhibiting development of a safety culture, and meaning that managers are often unaware that there is a problem with UB or how to address it.

While predicting how experience of UB erodes wellbeing varies from person to person, we found that UB can lead to loss of self-worth, burnout, a desire to exit the workplace, and, in some severe cases, suicidal ideation [14]. These outcomes can be even more severe in staff from minoritised groups. The impact on patient safety and care quality can also be severe, and we developed a programme theory which highlights the process by which this can occur (Fig. 10). We found that there is an interconnected causal chain from presence of UB through factors which distract staff, lower their confidence, and reduce their capacity to speak up about medical errors, all of which can significantly impact patient safety and care quality over time. Many of these factors, such as a reduced ability to speak up, are also mechanisms which themselves worsen the impact of UB. Likewise, many of these issues, such as a loss of passion for one’s job, and the impacts on care quality, can in themselves be compounded through staff turnover, loss of reputation, etc., to cause significant issues for healthcare organisations. A recent systematic review of the impact of unacceptable conduct between healthcare workers echoes these findings, showing that it negatively affects clinical performance, workplace productivity and quality of care, and results in negative patient outcomes [157]. Organisations must pay significant attention to fostering a psychologically safe organisational culture to minimise these poor outcomes.

### Strengths and limitations

This research had several strengths. The realist method, informed by the RAMESES standards [42], enabled us to formulate a coherent programme theory underpinning contributors to UB and their effects. We included a significant number of reports for a realist review with strong international representation. Our review search strategy was robust; it drew on a range of published and grey literature sources, use of multiple searches guaranteed we were drawing on literature likely to be relevant at different stages of the review, and searches were updated until December 2022.

As for limitations, we sought to understand factors which led to UB and not factors which can promote professionalism. As such, we may have missed some important elements which would be reflected in the pro-professionalism literature. Documents included in this review were limited to settings similar to UK NHS settings, with the exception of studies reporting an intervention (which we expanded to all settings). Therefore, the transferability and generalisability of findings is likely limited to being applicable to similar settings (i.e., publicly funded acute healthcare institutions). Further research may be needed to test these theories and explore transferability across different settings e.g. to primary care, or private healthcare institutions.

### Future research

The literature highlights that staff with particular characteristics, such as women, those from minoritised backgrounds, with a disability, and those who are LGBTQIA+ experience UB more frequently and more severely [10]. However, there is still limited understanding as to why this is the case. Some studies highlight, for example, that much of the increased experience of UB by staff with a disability may be “*correlated with several unreasonable management behaviours”* [144], i.e. that managers are not sufficiently accommodating of their conditions. Identifying contributors such as these that impact these groups could significantly aid the design of interventions to help address these imbalances and increase the current dearth of interventions focussed on such groups.

The systematic review by Keller et al. [14] also highlighted that there is minimal literature from the perspective of instigators of UB, meaning that reflections in the literature come only from those witnessing or experiencing it. Humans are predisposed to the fundamental attribution error [158] (in which people tend to *solely* attribute a person’s negative behaviour to their personality rather than acknowledging that often, behaviour is a combination of a person’s behaviour and their environment) when considering such events, meaning that they may consider an instigator of UB to have a personality disorder, when in actuality it may have been due to a confluence of factors such as overwork, stress, frustration, etc., that led to UB taking place. This is an important bias to acknowledge which can colour interpretation of this literature and help stop the favoured response of “it’s only a few bad apples”. While it may have practical and ethical difficulties, future research could try to understand contributors from the perspective of people who have behaved unprofessionally in the past, to further understand what they believe led to such behaviours.

## Conclusions

Contributors to UB include dysfunctional and toxic workplace processes and cultures, factors which lead to workplace disempowerment, inhibited social cohesion, a reduced ability to speak up, and lack of manager awareness and urgency to take remedial action. These contributors can lead to direct increases in UB, a reduced ability for staff to cope, and reduced ability to address UB. Contributors can interact to create further vicious cycles. Literature highlights that certain individuals and groups may be predisposed to engaging in UB or being susceptible to it; however, personality traits are not deterministic, and it is unclear how knowledge of these traits will better enable organisations to address UB. Organisations should instead focus on aspects under their control, i.e., understanding where UB is happening, and optimising their culture and processes to minimise the risk of UB. Mechanisms underlying how UB erodes staff wellbeing and patient safety includes negative impacts to ability to communicate, concentrate, and speak up about medical errors. Staff who are female, from minoritised backgrounds, or who have disabilities, are at greater risk of UB and its effects. Further research should investigate why this is the case and how these contributors can be addressed.

### Supplementary Information

**Additional file 1. **RAMESES Checklist.

**Additional file 2. **Initial theories for causes/contributors.

**Additional file 3. **Full search syntax and process description.

**Additional file 4. **Characteristics of included sources.

**Additional file 5. **Stakeholder feedback summary.

**Additional file 6. **Contributors, their programme theories, and illustrative quotes.

## Data Availability

The datasets used and/or analysed during the current study are available from the corresponding author on reasonable request.
