# Supplementary material for: Drivers of unprofessional behaviour between staff in acute care hospitals: a realist review
Source: BMC Health Serv Res. 2023 Nov 30;23:1326. doi: 10.1186/s12913-023-10291-3 (PMC10687856; doi:10.1186/s12913-023-10291-3)
Supplement: Supplementary file 2 — Additional file 2. Initial theories for causes/contributors. [file 12913_2023_10291_MOESM2_ESM.docx]

# Additional File 2. Initial theories for causes/contributors

Early on in our review process we surfaced our initial assumptions relating to the possible causes of UB. We determined a cause as:

Cause (i.e., environmental factor that can cause a change in reasoning) 🡪 Mechanism (change in reasoning) which increases proclivity to engage in UB 🡪 Outcome (usually increased UB)

At the time, we allocated numbers to reflect how well they were based on data which is indicated after each CMOC from 1-3. (1) indicated the CMOC was based on little data, (2) indicated partial data, and (3) meant that every aspect of the CMOC was supported by data. We did not retain this system in our final programme theory because we had greater data to rely on; however, understanding which theories were less supported by the literature helped us to direct our efforts during the refinement stage.

Table 1. Initial theories of causes.

| **Causes** | **CMOCs** |
| --- | --- |
| Individual factors | |
| 1. Perceived unfairness | A consistent sense of perceived unfairness in the workplace (e.g. a feeling that one is being wrongfully passed over for promotion) (C) leads to a feeling of anger and resentment (M) which increases chance of a negative response/becoming an instigator of UB (O) - 3 |
| 1. Personality disorders (instigator) | (Included but CMOC not formulated) |
| 1. Risk/reward analysis of the environment (instigator) | An environment that is not likely to punish an instigator, or otherwise rewards engaging in UB (e.g., a ‘bro’ culture) (C) makes workers perceive the environment as less risky for engaging in UB (M1) or that it may even be socially beneficial to engage in UB (M2) leading to increased proclivity to engage in UB (O) - 3 |
| 1. Interpretation of situation (victim) | If a victim has different expectations of social norms in their workplace (C) then they may interpret more forms of behaviour (i.e., criticism, feedback) as UB (M), therefore this may increase desire to reciprocate by engaging in UB, creating conflict (O1) or lead to more reports of UB (O2) - 3 |
| 1. Job demands (stress, pressure) vs. Job resources (sufficient time, autonomy, lack of organisational constraints) | Perpetual and unsustainable workplace pressure or a decrease in work resources (C) can lead to ineffective ability to communicate (e.g. due to time pressures) (M) which can lead to an increase in conflict (O) - 3  Perpetual and unsustainable workplace pressure or a decrease in work resources (C) can lead to reduced ability to cope (e.g. due to lack of time to break) (M) and therefore increase chance of inappropriate or unprofessional responses (O) – 3 |
| 1. Social support from colleagues | When social support is offered by colleagues (C) it can lead to an improved ability to cope (M1) and improved feeling of respect and belonging from colleagues (M2) which can reduce desire to reciprocate UB (O1) and mitigate impact of UB on mental health / wellbeing (O2) – 3 |
| 1. Role ambiguity (instigator) | Lack of clarity, control, or clear goals in job role (C) can lead to irritation and frustration (M1), and boredom (M2) which increases search for stimulating behaviours & likelihood to engage in conflict and UB (O) - 3 |
| 1. Physical discomfort | Physical discomfort due to conditions in the workplace (e.g. temperature, crowding) (C) can lead to increased lashing out (O) due to irritation and frustration (M) - 2 |
| 1. Role-modelling, social learning of UB norms, and the cycle of abuse (instigator) | Having a senior role model who frequently engages in UB with no consequence (C) could lead to social learning of inappropriate behavioural norms (M) and therefore increased desire to imitate /model the UB themselves (O) - 3 |
| Organisational factors | |
| 1. Hierarchies | Perceiving oneself to be in a low position in an organisational power hierarchy (C) reduces ability to communicate vertically within the hierarchy (M) and reduces psychological safety (M2) leading to inability to report mistakes by superiors (O) and increase chance of being a target for UB (O2) - 3  Perceiving oneself to be in a low position in an organisational power hierarchy (C) can lead to a reduced perception of respect for one’s role (M). This can increase anger/resentment leading to increased likelihood of engaging in UB (O1) – 3  Perceiving another person to be lower in the hierarchy as an instigator (C) makes the victim a perceptually less risky target than the instigator (M) leading to a higher chance of them becoming a victim (O) - 3 |
| 1. Leadership styles (destructive – autocratic) | Working under a leader using an autocratic leadership style (C) can lead to increased role-modelling of UB by employees (M1), and normalisation of UB in organisational culture (M2) which can lead to increased UB (O) - 3  Working under a leader using an autocratic leadership style (C) can lead to reduced sense of psychological safety (M3) which can lead to inability to speak up about UB (O2) - 3 |
| 1. Leadership styles (destructive – laissez-faire) | Working under leaders who are laissez-faire or ‘hands-off’ (C) can lead to the perception by instigators that there is low risk to engage in UB, as there’s little chance of punishment (M1) leading to increased proclivity to engage in UB (O) - 3  Working under leaders who are laissez-faire or ‘hands-off’ (C) can lead to increased role ambiguity (M1) leading to increased boredom (O1) and frustration (O2) - 3 |
| 1. Leadership styles (destructive – transactional) | Working under a transactional leader (C) can lead to a culture of tolerance of mistreatment (M1) and reduction in risk for instigators to engage in UB (M2) leading to increased proclivity to engage in UB & to become an instigator (O) - 3 |
| 1. Leadership styles (destructive – tyrannical) | Working under a tyrannical leader (C) can lead to a reduction in feeling of social support (M1) and a reduction in psychological safety (M2) leading to an increased chance of becoming a victim of UB (O) and increased personal impact of being exposed to UB (O2) - 2 |
| 1. Leadership styles (constructive – transformational, democratic, or compassionate) | Working under a constructive leader (C) can lead to an increased sense of psychological safety (M) and increased ability to speak up (O) - 3  Working under a constructive leader (C) can lead to an increased perception of risk for potential instigators (M) and reduction in desire to engage in UB (O) - 3  Working under a constructive leader (C) can lead to a reduction in role ambiguity (M) and therefore a reduction in boredom and frustration (O) - 3  Working under a democratic leader (C) can lead to an increased sense of social support from colleagues (M) and therefore improved ability to cope (O1) and improved sense of respect (O2) - 3 |
| 1. Organisational change | Working amidst significant organisational reorganisation or turnover (C) reduces feelings of psychological safety (M1) and can increase uncertainty about one’s organisational role (M2) leading to greater competition, bullying, and incivility between staff (O) - 3  Change in organisational leadership (C) can enlarge the power gap between employees and leaders (M) leading to a reduction in psychological safety (O) - 3 |
| 1. Organisational culture (Tolerance of unprofessional behaviours) | A perceived tolerance of UB in the organisation or team (C) can lead to a lowering of risk to engage in UB by instigators (M) leading to increased proclivity to engage in UB (O) - 3  A perceived tolerance of UB in the organisation or team (C) can lead to an increase in perceived role modelling of UB (M) leading to increased proclivity to engage in UB (O) - 3  A perceived tolerance of UB in the organisation or team (C) can lead to ridiculing of professional behaviours such as acting compassionate (M) leading to reduced desire to act against the social norm where UB is prevalent (O) – 3  Witnessing UB occur without negative consequence in the organisational environment (C) can lead to increased perception that UB is acceptable (M1) and reduce perception of risk to engage in UB (M2) leading to increased proclivity to engage in UB (O) - 3 |
| 1. Organisational culture – (Belonging to an ‘outgroup’ or workplace minority in intolerant work environment) | Being a member of a minority group in a workplace that is intolerant towards minorities (C) can lead to a reduced perception by minority members that they have social support (M1) and may appear as being in lower perceived position in the power hierarchy to potential instigators (M2) which may make such minority members more susceptible to experiencing UB (O1) and make victims less likely to speak up (O2) - 3 |
| 1. Organisational culture (oppression of certain workers) | Workers from professional groups that have a lack of power in their organisational structure (C) may engage in greater unprofessional behaviours (O) because they can internalise the oppression and lash out due to frustration (M) – 2  Being a member of an oppressed professional group in an organiastion (C) can make you more likely to be a target of UB (O) due to being seen as a less risky target for instigators of UB (M) - 2 |
| 1. Organisational culture (integrity and fairness) | When staff members feel undervalued, underappreciated and oppressed within their workplaces (C), they may internalise the oppression (M1) causing a decrease in empathy for one another (M2) and in turn become bullies of those below or equal to themselves (O) - 3 |
| 1. Organisational culture (social norm of prolonged and unnecessary high stress) | In cultures of high stress and high stakes for patient safety, (C) staff may experience anger, frustration and fear (M) making them more likely to lash out and produce an environment rife with conflict (O) - 3 |
| Team factors | |
| 1. Collaboration across departments | If there is significant collaboration across departments when teamworking (C) then there may be an increased chance of being exposed to a perceived interprofessional power hierarchy (M) which can increase chance of conflict (O1) and reduce effective communication (O2) - 2 |
| 1. Team leaders having unreasonable expectations | When senior members of staff have high expectations that feel unreasonable to junior staff members (e.g., a culture of perfectionism) (C), working relationships become tense and unpleasant (M), which creates an environment for bullying to occur (O) - 3 |
| 1. Team - Lacking a common vision/direction | If the team lacks a common vision (C) then this may increase sense of frustration (M) which can lead to an increase in conflict and UB between team members (O) - 2  When a team lacks common direction or purpose (C) then boredom/frustration can lead to selection of scapegoats within the team (M) which can lead to targeted forms of UB (O) - 2 |
| 1. Team leadership | A more democratic team leadership style (C) can lead to an improved sense of social support (M1) and psychological safety (M2) which can therefore reduce the impact of UB (O) - 3 |
| 1. Shift working | If team members are working in shifts (C) then they may not be able to build interpersonal relationships with their co-workers (M) leading to an absence of social support (O) - 3 |
| Societal factors | |
| 1. Public pressure | If the public becomes aware through media (or other) reports about high prevalence of UB in an organisation (C) then this can lead to a sense of pressure on management to resolve the problem (M) which can reduce UB (O) - 3 |
| 1. Regulation | If there is regulation in place to reduce UB or punish it, and this is implemented by organisations (C) then this may increase perception of risk for the instigator due to fear of breaking organisational policies and the law (M), leading to a reduction in desire to engage in UB (O) – 2 |


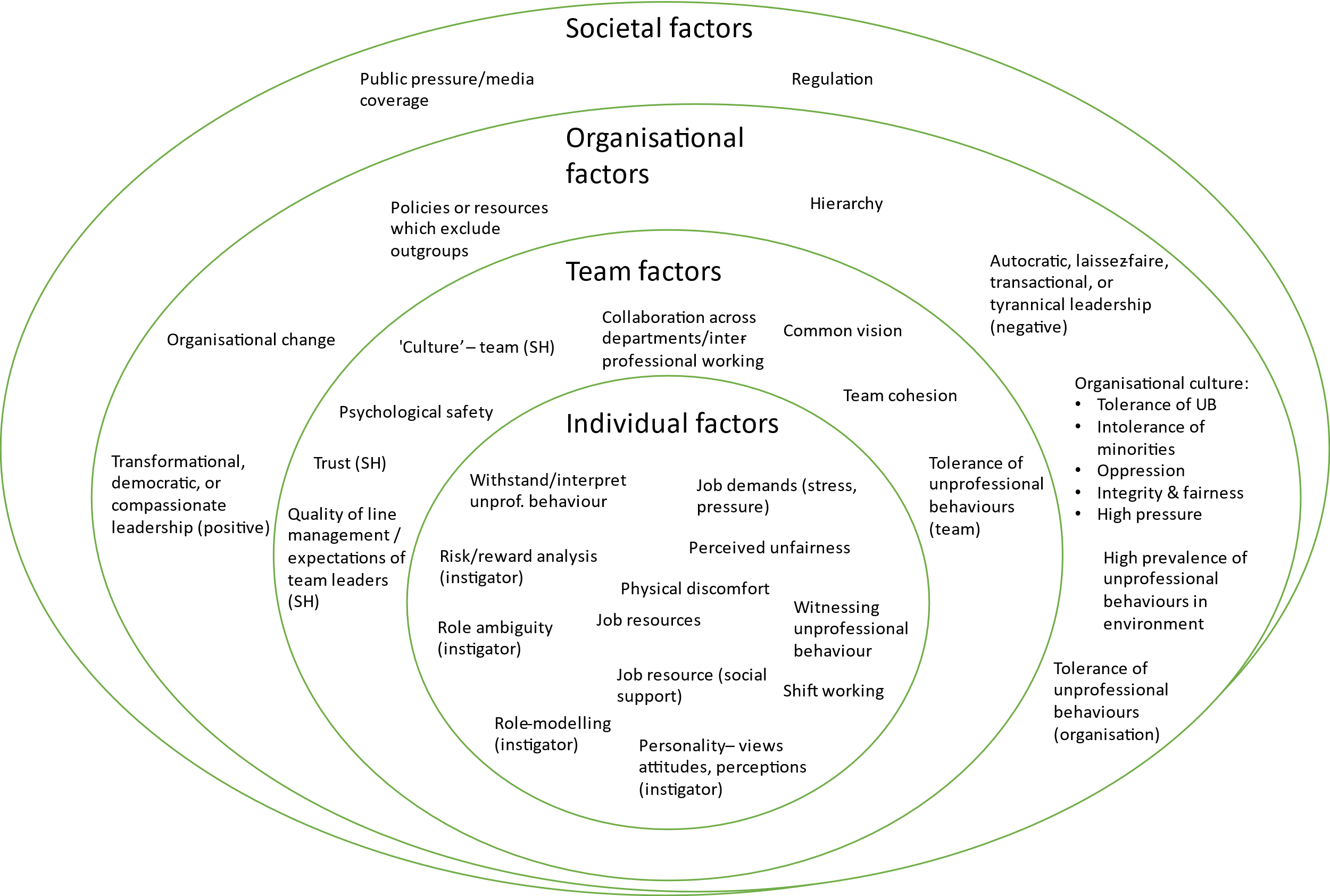


Figure 1. Initial programme theory for causes.
