# Supplementary material for: Drivers of unprofessional behaviour between staff in acute care hospitals: a realist review
Source: BMC Health Serv Res. 2023 Nov 30;23:1326. doi: 10.1186/s12913-023-10291-3 (PMC10687856; doi:10.1186/s12913-023-10291-3)
Supplement: Supplementary file 3 — Additional file 3. Full search syntax and process description. [file 12913_2023_10291_MOESM3_ESM.docx]

# Additional File 3. Full search syntax and process description.

This Additional File outlines our full search process and full list of search strategies used throughout the review across multiple databases and timepoints. These are outlined below.

## Full description of search process

### Results of searching and screening

The following results are in chronological order and reflect multiple cycles of searches, screening, and relevancy/rigour assessments. The PRISMA diagram in the manuscript, however, displays the process of searching, screening and inclusion in a non-chronological order, i.e., the update search results are incorporated with the initial search results in the diagram. This demonstrates more clearly how many studies were found, included, and excluded, at Step 1 vs. Step 2 of the review.

#### Step 1 (November 2021): Identifying existing theories and scoping the literature

We included 38 documents in the initial theory generation step, comprising 30 identified from the proposal, five from informal searches, and three from the team.

#### Step 2 (November 2021 – February 2022): Searching for evidence

The initial systematic search identified 2,629 records after 99 Google Scholar search results after cross-database deduplication. Initial independent pilot screening of 54 (of 2,629) records resulted in 16 papers included for full text screening with 100% agreement. Independent screening by two reviewers (JA and RA) of 267 (10% of 2,629) records led to 72 being included for the next step. Disagreement occurred on 40 items (15%) and was resolved through discussion between JA and RA. The remaining 2,308 papers were screened by JA against the inclusion/exclusion criteria. Of these, 400 papers were selected for full text screening.

##### Google and Google Scholar Searches (November 2021)

Two searches were performed on Google Scholar via use of Harzing’s Publish or Perish software (each was limited to the 50 most relevant results). This was to ensure we captured relevant grey or academic literature identified by Google’s algorithms. Search strategies are presented in Appendix 3. After duplicate removal, 99 papers remained for title and abstract screening. Papers were excluded largely because they were not related to unprofessional behaviours between staff. Sixty-three papers were selected for full text screening and then combined with the systematic search results at the full text screening stage. An additional 52 sources were identified through searches on Google (30) in 2021, from the project team (12) and from stakeholders (10) during 2022 (see Additional searching below). In total, 603 papers were selected for full-text screening. After cross-deduplication of these various sources of literature, 537 full-text papers were eligible for screening.

##### Step 2: Full text screening

Following application of major/minor screening criteria to the 537 potentially relevant papers, 193 papers were determined to have major relevancy, and 352 were excluded for having minor relevancy. In addition, 34 (10%) of those papers excluded at this stage were selected for independent screening (JA and RA). From these, two decisions were found to be in conflict. These two discrepancies were resolved through discussion and remained excluded. The 193 papers were then screened against inclusion criteria and conceptual richness and 148 papers were included.

##### Relevance and rigour

The remaining 148 papers were screened for relevancy according to the realist method, meaning papers had to include passages suitable for theory gleaning, testing, or refining with respect to either causes or strategies(1). Studies which lacked such passages were therefore screened out (n=45) resulting in 103 papers. An additional 6 studies from the team and 2 studies from citation tracking key intervention papers were added at this stage, thus, 111 documents were included for conceptual thickness screening.

##### Conceptual thickness screening

As a result of conceptual thickness screening as outlined in Table 2 above, 47 sources were excluded at this stage for lacking conceptual thickness. This meant that 64 rich sources were included at this stage.

#### Additional searching and evidence gathering (August – November 2022)

##### Search update to expand relevancy criteria to USA intervention studies (August 2022)

The team decided to include USA-based literature because earlier searches had not identified any UK interventions. We reran the same searches for ‘step 2’ but limited result to USA (excluding HMIC due to its predominance of UK content, and NHS Evidence which was withdrawn in April 2022). This identified 1,298 records, which reduced to 57 records once duplicates and previously screened records were removed. These 57 records were screened but none were included because no interventions were identified. However, we did re-include 10 USA studies from our step 2 search which had previously been excluded because of country.

A further 9 relevant studies were identified by citation searching (forwards and backwards) from nine key further USA studies using the CitationChaser Shiny App(2). Four studies were identified by the team. Reference scanning of the 10 included USA studies and the 4 identified by the team found a further 11 and 2, resulting in a total of 36 additional papers from August 2022 searches. A total of 138 papers were included (step 1 and 2) prior to final update searches.

##### Searches for behavioural and organisational psychology theories (November 2022)

Members of the advisory group indicated that literature from behavioural or organisational psychology may provide useful evidence, so we searched for anti-bullying interventions that use behavioural science theory in: ABI/INFORM Collection (ProQuest), Business Source Premier (Ebscohost) and Google Scholar. The lack of relevant search results prompted a further search (in ABI/INFORM Collection (ProQuest) and Google Scholar). Due to low relevancy of results during screening, papers found in this search were not utilised.

#### Search update (December 2022)

The above searches were repeated on 9^th^ December 2022 (except for NICE Evidence (withdrawn in April 2022)) to ensure our review was as up to date. The search strategies were reviewed before running the final update searches and no changes made. This identified 3890 records, which reduced to 192 records when we removed duplicates and previously identified records. After title and abstract screening, 36 papers were retained for full text screening. Included from this update were 8 papers and an additional 2 studies from the team (10 total).

### Total included literature (March 2023)

148 total papers were included. This included 38 papers from step 1; 100 papers from step 2; and 10 from our updated December 2022 search.

### Final contributor screening

We then further screened sources for information relevant to the contributors to UB. In total, we included 28 documents for initial theory building (3,4,13–22,5,23–30,6–12) and 81 for theory refinement (26,31,40–49,32,50–59,33,60–69,34,70–79,35,80–89,36,90–99,37,100–109,38,110–112,39), comprising 109 sources.

## List of search syntaxes

1. Google Scholar search 2021
2. Google Search 2021
3. Systematic search (multiple sources) 15-02-2022
4. USA update search (multiple sources) 25-08-2022
5. CitationChaser search 25-08-2022
6. Update Systematic Search (multiple sources) 09-12-2022

#### 1. Google Scholar (Via Harzing’s Publish or Perish)

Google Scholar (Via Harzing’s Publish or Perish)

Date searched: 19-11-2021

Records downloaded: 100

Search 1. 446 records found, first 50 downloaded (sorted by relevance)

bullying | harassment | discrimination|unprofessional AND workplace|worker|staff AND model|framework|concept|idea|opinion|theory|view|perception|attitude|theories [Searched in Publish or Perish Title Field]

Search 2. ~1,120,000 records found, first 50 downloaded (sorted by relevance)

interventions|strategies|techniques|program|programs|programme|programmes AND unprofessional|bullying|harassment|discrimination AND nurse|doctor|paramedic|hospital|ambulance|staff|professional AND emergency|acute|trauma [searched in Publish or Perish Keywords field]

#### Google

Date searched: 23-11-2021

Records browsed: First 5 pages

allintext: interventions|strategies|strategy|techniques|program|programs|programme|programmes unprofessional|bullying|harassment|discrimination nurse|doctor|paramedic|hospital|ambulance|staff|professionals emergency|acute

#### 2. Systematic search 15-02-2022

Sources searched:

- CINAHL (EBSCOhost)
- Embase Classic+Embase (Ovid) 1947 to 2022 February 11
- Ovid MEDLINE(R) ALL 1946 to February 11, 2022
- HMIC Health Management Information Consortium 1979 to November 2021
- NICE Evidence Search https://www.evidence.nhs.uk/
- Patient Safety Network <https://psnet.ahrq.gov/>

CINAHL (EBSCOhost)

Date searched: 14-02-2022

Records found: 1279

# Query Results

S37 S35 NOT S36 1,279

S36 TX ( ("elder mistreat*" or "elder abuse*" or "elder neglect*") ) 4,823

S35 S33 NOT S34 1,322

S34 ( ( (MH "Child") OR (MH "Adolescence+") OR (MH "Minors (Legal)") ) NOT (MH "Adult+") ) 469,789

S33 S22 NOT S32 1,391

S32 S28 NOT S31 365,968

S31 S29 OR S30 1,683,919

S30 (MH "Australia+") OR (MH "Europe") OR (MH "Austria") OR (MH "Baltic States+") OR (MH "Belgium") OR (MH "Canada+") OR (MH "Chile") OR (MH "Colombia") OR (MH "Costa Rica") OR (MH "Czech Republic") OR (MH "Scandinavia+") OR (MH "France") OR (MH "Germany+") OR (MH "Greece") OR (MH "Hungary") OR (MH "Iceland") OR (MH "Ireland") OR (MH "Italy") OR (MH "Israel") OR (MH "Japan") OR (MH "South Korea") OR (MH "Luxembourg") OR (MH "Mexico") OR (MH "Netherlands") OR (MH "New Zealand") OR (MH "North America") OR (MH "Poland") OR (MH "Portugal") OR (MH "Slovakia") OR (MH "Slovenia") OR (MH "Spain") OR (MH "Switzerland") OR (MH "Turkey") OR (MH "United Kingdom+") OR (MH "United States+") 1,677,371

S29 (MH "Developed Countries") or (MH "European Union") or (MH "Organisation for Economic Co-Operation and Development") 10,234

S28 (S23 OR S24 OR S25 OR S26 OR S27) 404,118

S27 (MH "Africa+") 92,227

S26 (MH "Asia, Southeastern+") OR (MH "China+") OR (MH "Hong Kong") OR (MH "Macao") OR (MH "Mongolia") OR (MH "North Korea") OR (MH "Taiwan") OR (MH "Atlantic Islands") OR (MH "Indian Ocean Islands+") OR (MH "Melanesia+") OR (MH "Micronesia+") OR (MH "Polynesia+") 127,545

S25 (MH "Bangladesh") OR (MH "Bhutan") OR (MH "India") OR (MH "Yemen") OR (MH "United Arab Emirates") OR (MH "Syria") OR (MH "Saudi Arabia") OR (MH "Qatar") OR (MH "Oman") OR (MH "Lebanon") OR (MH "Kuwait") OR (MH "Jordan") OR (MH "Iraq") OR (MH "Iran") OR (MH "Bahrain") OR (MH "Afghanistan") OR (MH "Nepal") OR (MH "Pakistan") OR (MH "Sri Lanka") OR (MH "Asia, Central+") 108,764

S24 (MH "Argentina") OR (MH "Bolivia") OR (MH "Brazil") OR (MH "Ecuador") OR (MH "French Guiana") OR (MH "Guyana") OR (MH "Paraguay") OR (MH "Peru") OR (MH "Suriname") OR (MH "Uruguay") OR (MH "Venezuela") OR (MH "Belize") OR (MH "El Salvador") OR (MH "Guatemala") OR (MH "Honduras") OR (MH "Nicaragua") OR (MH "Panama+") or (MH "West Indies+") 72,856

S23 (MH "Albania") OR (MH "Andorra") OR (MH "Armenia") OR (MH "Azerbaijan") or (MH "Byelarus") OR (MH "Bosnia-Herzegovina") OR (MH "Croatia") OR (MH "Bulgaria") OR (MH "Georgia (Republic)") OR (MH "Gibraltar") OR (MH "Liechtenstein") OR (MH "Macedonia (Republic)") OR (MH "Moldova") OR (MH "Monaco") OR (MH "Romania") OR (MH "Russia") OR (MH "San Marino") OR (MH "Serbia") OR (MH "Ukraine") OR (MH "Yugoslavia") 11,801

S22 S5 AND S21 1,505

S21 S16 OR S17 OR S18 OR S19 OR S20 23,637

S20 TI ( (staff or employee* or work* or nurs* or doctor#) n5 (sexism or sexist or ableis* or racism or racist or (racial n3 abus*)) ) OR AB ( (staff or employee* or work* or nurs* or doctor#) n5 (sexism or sexist or ableis* or racism or racist or (racial n3 abus*)) ) OR SU ( (staff or employee* or work* or nurs* or doctor#) n5 (sexism or sexist or ableis* or racism or racist or (racial n3 abus*)) ) 661

S19 TI ( ((staff or employee* or work* or nurs* or doctor#) n6 ((gender or disabilit* or disabled) n4 (inequalit* or equalit*))) OR ((staff or employee* or nurs* or doctor?) n1 (aggressi* or abus*))) OR AB ( ((staff or employee* or work* or nurs* or doctor#) n6 ((gender or disabilit* or disabled) n4 (inequalit* or equalit*))) OR ((staff or employee* or nurs* or doctor?) n1 (aggressi* or abus*)) ) OR SU ( ((staff or employee* or work* or nurs* or doctor#) n6 ((gender or disabilit* or disabled) n4 (inequalit* or equalit*))) OR ((staff or employee* or nurs* or doctor?) n1 (aggressi* or abus*)) ) 1,978

S18 ((MH "Workplace Violence") or (MH "Aggression") or (MH "Prejudice+") or (MH "Discrimination+") or (MH "Dehumanization") OR (MH "Oppressed Group Behavior") OR (MH "Coercion")) AND ( (MH "Interprofessional Relations+") or (MH "Intraprofessional Relations")) 1,028

S17 TI ( ((staff or employee* or work* or nurs* or doctor#) n4 (hostil* or undermin* or discrimination or discriminatory or discriminated or humiliat*) ) OR ((staff or employee* or work* or nurs* or doctor#) n4 (WPV or violen* or victim* or coerci*) n3 among*) OR "abusive supervision" OR (workplace n2 (conflict* or aggressi* or abus*)) ) OR AB ( ((staff or employee* or work* or nurs* or doctor#) n4 (hostil* or undermin* or discrimination or discriminatory or discriminated or humiliat*) ) OR ((staff or employee* or work* or nurs* or doctor#) n4 (WPV or violen* or victim* or coerci*) n3 among*) OR "abusive supervision" OR (workplace n2 (conflict* or aggressi* or abus*)) ) OR SU ( ((staff or employee* or work* or nurs* or doctor#) n4 (hostil* or undermin* or discrimination or discriminatory or discriminated or humiliat*) ) OR ((staff or employee* or work* or nurs* or doctor#) n4 (WPV or violen* or victim* or coerci*) n3 among*) OR "abusive supervision" OR (workplace n2 (conflict* or aggressi* or abus*)) ) 3,111

S16 S9 AND S15 18,280

S15 S10 OR S11 OR S12 OR S13 OR S14 2,214,829

S14 (MH "Health Personnel+") or (MH "Students, Health Occupations+") or (MH "Internship and Residency") OR (MH "Education, Graduate") OR (MH "Teamwork") 694,847

S13 TI ( (student# n2 (health* or clinic* or medic*)) OR employee# or worker# or Staff or personnel or practitioner# or professional# or workforce* or workplace* or "work place*" or worksite* or "work site*" or "work setting*" OR team*) OR AB ( (student# n2 (health* or clinic* or medic*)) OR employee# or worker# or Staff or personnel or practitioner# or professional# or workforce* or workplace* or "work place*" or worksite* or "work site*" or "work setting*" OR team*) OR SU ( (student# n2 (health* or clinic* or medic*)) OR employee# or worker# or Staff or personnel or practitioner# or professional# or workforce* or workplace* or "work place*" or worksite* or "work site*" or "work setting*" OR team*) 1,086,560

S12 TI ( Pathologist# or P#ediatrician# or Physiatrist# or Psychiatrist# or Pulmonologist# or Radiographer# or Radiologist# OR assistant# or cleaner# or ancillary or porter# or auxillary or auxillaries or administrator# or secretary or secretaries or receptionist# or technician# ) OR AB ( Pathologist# or P#ediatrician# or Physiatrist# or Psychiatrist# or Pulmonologist# or Radiographer# or Radiologist# OR assistant# or cleaner# or ancillary or porter# or auxillary or auxillaries or administrator# or secretary or secretaries or receptionist# or technician# ) OR SU ( Pathologist# or P#ediatrician# or Physiatrist# or Psychiatrist# or Pulmonologist# or Radiographer# or Radiologist# OR assistant# or cleaner# or ancillary or porter# or auxillary or auxillaries or administrator# or secretary or secretaries or receptionist# or technician# ) 153,037

S11 TI ( Audiologist# or Anatomist# or Allergist# or An#esthetist# or An#esthesiologist# or Cardiologist# or Dieti#ian# or Endocrinologist# or Gastroenterologist# or GP# or Geriatrician# or Hospitalist# or Oncologist# OR Ophthalmologist# or Otolaryngologist# ) OR AB ( Audiologist# or Anatomist# or Allergist# or An#esthetist# or An#esthesiologist# or Cardiologist# or Dieti#ian# or Endocrinologist# or Gastroenterologist# or GP# or Geriatrician# or Hospitalist# or Oncologist# OR Ophthalmologist# or Otolaryngologist# ) OR SU ( Audiologist# or Anatomist# or Allergist# or An#esthetist# or An#esthesiologist# or Cardiologist# or Dieti#ian# or Endocrinologist# or Gastroenterologist# or GP# or Geriatrician# or Hospitalist# or Oncologist# OR Ophthalmologist# or Otolaryngologist# ) 85,337

S10 TI ( ( nurs* or midwif* or midwiv or paramedic# or doctor# or physician# or clinician# or surgeon# or consultant# OR medic# or intern# or resident# or Therapist# or Pharmacist# or Optometrist# or Nutritionist# or Dentist# or Physiotherapist# ) ) OR AB ( ( nurs* or midwif* or midwiv or paramedic# or doctor# or physician# or clinician# or surgeon# or consultant# OR medic# or intern# or resident# or Therapist# or Pharmacist# or Optometrist# or Nutritionist# or Dentist# or Physiotherapist# ) ) OR SU ( ( nurs* or midwif* or midwiv or paramedic# or doctor# or physician# or clinician# or surgeon# or consultant# OR medic# or intern# or resident# or Therapist# or Pharmacist# or Optometrist# or Nutritionist# or Dentist# or Physiotherapist# ) ) 1,499,154

S9 S6 OR S7 OR S8 31,363

S8 TI ( ( ((lateral* or horizontal*) n2 violence) ) OR ( ((transgressive or disruptive or unprofessional) n2 behavio#r*) ) OR (professional* n2 misconduct) OR ( "negative behavio*" or "negative act" ) ) OR AB ( ( ((lateral* or horizontal*) n2 violence) ) OR ( ((transgressive or disruptive or unprofessional) n2 behavio#r*) ) OR (professional* n2 misconduct) OR ( "negative behavio*" or "negative act" ) ) OR SU ( ( ((lateral* or horizontal*) n2 violence) ) OR ( ((transgressive or disruptive or unprofessional) n2 behavio#r*) ) OR (professional* n2 misconduct) OR ( "negative behavio*" or "negative act" ) ) 11,945

S7 TI ( bully* or harass* or intimidat* or micro-aggress* or microaggress* or incivil* or uncivil* or rude* or mistreat* or mobbing or hazing or gaslight* or malic* or (Hidden n5 (aggressi* or abus* or violenc*))) OR AB ( bully* or harass* or intimidat* or micro-aggress* or microaggress* or incivil* or uncivil* or rude* or mistreat* or mobbing or hazing or gaslight* or malic* or (Hidden n5 (aggressi* or abus* or violenc*))) OR SU ( bully* or harass* or intimidat* or micro-aggress* or microaggress* or incivil* or uncivil* or rude* or mistreat* or mobbing or hazing or gaslight* or malic* or (Hidden n5 (aggressi* or abus* or violenc*))) 18,468

S6 (MH "Disruptive Behavior") or (MH "Bullying+") OR (MH "Emotional Abuse") OR (MH "Verbal Abuse") or (MH "Sexual Harassment") or (MH "Professional Misconduct") or (MH "Incivility") or (MH "Scapegoating") 22,192

S5 S1 OR S2 OR S3 OR S4 505,019

S4 TI ( ( (critical* n2 (care or ill*)) ) OR ( (urgent n2 (care or service* or medic*)) ) OR "intensive care" OR paramedic*) OR AB ( ( (critical* n2 (care or ill*)) ) OR ( (urgent n2 (care or service* or medic*)) ) OR "intensive care" OR paramedic*) OR SU ( ( (critical* n2 (care or ill*)) ) OR ( (urgent n2 (care or service* or medic*)) ) OR "intensive care" OR paramedic*) 165,198

S3 TI ( ( ((trauma* or ambulan*) n4 (care or service* or ill* or unit* or centre* or centre* or department* or setting)) ) ) OR AB ( ( ((trauma* or ambulan*) n4 (care or service* or ill* or unit* or centre* or centre* or department* or setting)) ) ) OR SU ( ( ((trauma* or ambulan*) n4 (care or service* or ill* or unit* or centre* or centre* or department* or setting)) ) ) 18,725

S2 TI ( ( ((emergenc* or acute*) n4 (care or service* or health* or ill* or treat* or medic* or unit* or centre* or centre* or department* or setting* or ward#)) ) ) OR AB ( ( ((emergenc* or acute*) n4 (care or service* or health* or ill* or treat* or medic* or unit* or centre* or centre* or department* or setting* or ward#)) ) ) OR SU ( ( ((emergenc* or acute*) n4 (care or service* or health* or ill* or treat* or medic* or unit* or centre* or centre* or department* or setting* or ward#)) ) ) 246,695

S1 (MH "Emergency Medical Services+") or (MH "Emergency Treatment+") or (MH "Emergency Care") or (MH "Airway Management+") or (MH "Ambulatory Care") OR (MH "Acute Care") or (MH "Critical Care+") OR (MH "Perioperative Care") OR (MH "Preoperative Care+") or (MH "Critical Care Nursing+") or (MH "Emergency Nursing+") 303,236

1. Embase Classic+Embase (Ovid) 1947 to 2022 February 11

Date searched: 14-02-2022

Records found: 1319

1 emergency health service/ or emergency medical dispatch/ or hospital emergency service/ or psychiatric emergency service/ 118852

2 emergency treatment/ or evidence based emergency medicine/ 18594

3 emergency care/ or advanced trauma life support/ or emergency ward/ 219986

4 respiration control/ or exp assisted ventilation/ or exp artificial ventilation/ 302732

5 exp ambulatory care/ or exp intensive care/ 817957

6 perioperative nursing/ or exp perioperative period/ or exp preoperative care/ 111940

7 exp hotline/ or poison center/ or exp ambulance/ 22714

8 exp intensive care nursing/ or emergency nursing/ 9915

9 (emergenc* adj5 (care or service* or health* or ill* or treat* or medic* or unit* or centre* or centre* or department* or setting*)).tw,kf. 297016

10 (acute* adj5 (care or service* or health* or ill* or treat* or medic* or unit* or centre* or centre* or department* or setting* or ward?)).tw,kf. 332752

11 (trauma* adj5 (care or service* or ill* or unit* or centre* or centre* or department*)).tw,kf. 36375

12 (ambula* adj5 (care or service* or unit* or centre* or centre* or department* or setting*)).tw,kf. 38326

13 (critical* adj2 (care or ill*)).tw,kf. 145798

14 (urgent adj3 (care or service* or medic*)).tw,kf. 8305

15 "intensive care".tw,kf. 255322

16 paramedic*.tw,kf. 13838

17 or/1-16 [Acute Care or Ambulance services] 1840430

18 agonistic behavior/ 193

19 exp bullying/ 9218

20 disruptive behavior/ 3154

21 harassment/ or non-sexual harassment/ or exp online harassment/ or exp sexual harassment/ 5032

22 incivility/ 363

23 microaggression/ 181

24 professional misconduct/ 3804

25 exp hostility/ 14279

26 bully*.tw,kf. 8592

27 harass*.tw,kf. 5336

28 intimidat*.tw,kf. 1973

29 (lateral* adj2 violence).tw,kf. 113

30 (horizontal* adj2 violence).tw,kf. 153

31 (transgressive adj3 behavio?r*).tw,kf. 46

32 (disruptive adj3 behavio?r*).tw,kf. 5605

33 (unprofessional adj3 behavio?r*).tw,kf. 479

34 (micro-aggress* or microaggress*).tw,kf. 562

35 incivil*.tw,kf. 688

36 uncivil*.tw,kf. 217

37 rude*.tw,kf. 1299

38 mistreat*.tw,kf. 3110

39 (professional* adj3 misconduct).tw,kf. 363

40 mobbing.tw,kf. 525

41 (negative behavio* or negative act?).tw,kf. 2078

42 hazing.tw,kf. 97

43 (gaslight* or malic*).tw,kf. 9947

44 (Hidden adj5 (aggressi* or abus* or violenc*)).tw,kf. 247

45 or/18-44 [Unprofessional behaviours] 61184

46 exp health care personnel/ 1827002

47 exp health student/ 129944

48 exp medical education/ 364812

49 public relations/ 63422

50 workplace/ 48445

51 teamwork/ 20182

52 (nurs* or midwif* or midwiv*).tw,kf. 620312

53 paramedic?.tw,kf. 9205

54 (doctor? or physician? or clinician? or surgeon? or consultant?).tw,kf. 1496063

55 (student? adj2 (medic* or health* or clinic*)).tw,kf. 88543

56 intern?.tw,kf. 16380

57 resident?.tw,kf. 239903

58 (Therapist? or Pharmacist? or Optometrist? or Nutritionist? or Dentist? or Physiotherapist?).tw,kf. 218724

59 (Audiologist? or Anatomist? or Allergist? or An?esthetist? or An?esthesiologist? or Cardiologist? or Dieti#ian? or Endocrinologist? or Gastroenterologist? or GP? or Geriatrician? or Hospitalist? or Oncologist?).tw,kf. 362665

60 (Ophthalmologist? or Otolaryngologist? or Pathologist? or P?ediatrician? or Physiatrist? or Psychiatrist? or Pulmonologist? or Radiographer? or Radiologist?).tw,kf. 281398

61 medic?.tw,kf. 42012

62 assistant?.tw,kf. 41928

63 (cleaner? or ancillary or porter?).tw,kf. 35085

64 (auxillary or auxillaries or administrator? or secretary or secretaries or receptionist? or technician?).tw,kf. 57136

65 (employee? or worker? or staff or personnel or practitioner? or professional? or workforce* or team*).tw,kf. /freq=2 573697

66 (workplace* or "work place*" or worksite* or "work site*" or "work setting*").tw,kf. 70577

67 or/46-66 [Staff] 4107447

68 45 and 67 [UB and Staff search 1] 18031

69 public relations/ and (aggression/ or aggressiveness/ or prejudice/ or exp social discrimination/ or coercion/) 419

70 ((staff or employee* or work* or nurs* or doctor?) adj8 (WPV or violen*) adj5 among*).tw,kf. 597

71 ((staff or employee* or work* or nurs* or doctor?) adj8 Victim* adj5 among*).tw,kf. 131

72 ((staff or employee* or work* or nurs* or doctor?) adj8 (humiliat* or hostil*)).tw,kf. 734

73 ((staff or employee* or work* or nurs* or doctor?) adj3 undermin*).tw,kf. 368

74 ((staff or employee* or work* or nurs* or doctor?) adj6 (discrimination or discriminatory or discriminated)).tw,kf. 2949

75 "abusive supervision".tw,kf. 121

76 (workplace adj3 (conflict* or aggressi* or abus*)).tw,kf. 634

77 ((staff or employee* or work* or nurs* or doctor?) adj5 (gender adj2 (inequalit* or equalit*))).tw,kf. 191

78 ((staff or employee* or work* or nurs* or doctor?) adj5 (racism or racist or (racial adj3 abus*))).tw,kf. 355

79 ((staff or employee* or work* or nurs* or doctor?) adj5 (sexism or sexist)).tw,kf. 80

80 ((staff or employee* or work* or nurs* or doctor?) adj8 ((disabilit* or disabled) adj5 (inequalit* or equalit*))).tw,kf. 20

81 ((staff or employee* or work* or nurs* or doctor?) adj8 ableis*).tw,kf. 10

82 ((staff or employee* or work* or nurs* or doctor?) adj8 coerci* adj5 among*).tw,kf. 11

83 ((staff or employee* or nurs* or doctor?) adj1 (aggressi* or abus*)).tw,kf. 275

84 or/69-83 [UB and staff search 2] 6650

85 68 or 84 [UB among Staff final search] 23586

86 17 and 85 [UB among Staff in Acute Care] 1669

87 afghanistan/ or africa/ or "africa south of the sahara"/ or albania/ or algeria/ or andorra/ or angola/ or argentina/ or "antigua and barbuda"/ or armenia/ or exp azerbaijan/ or bahamas/ or bahrain/ or bangladesh/ or barbados/ or belarus/ or belize/ or benin/ or bhutan/ or bolivia/ or borneo/ or exp "bosnia and herzegovina"/ or botswana/ or exp brazil/ or brunei darussalam/ or bulgaria/ or burkina faso/ or burundi/ or cambodia/ or cameroon/ or cape verde/ or central africa/ or central african republic/ or chad/ or exp china/ or comoros/ or congo/ or cook islands/ or coted'ivoire/ or croatia/ or cuba/ or cyprus/ or democratic republic congo/ or djibouti/ or dominica/ or dominican republic/ or ecuador/ or el salvador/ or egypt/ or equatorial guinea/ or eritrea/ or eswatini/ or ethiopia/ or exp "federated states of micronesia"/ or fiji/ or gabon/ or gambia/ or exp "georgia (republic)"/ or ghana/ or grenada/ or guatemala/ or guinea/ or guinea-bissau/ or guyana/ or haiti/ or honduras/ or exp india/ or exp indonesia/ or iran/ or exp iraq/ or jamaica/ or jordan/ or kazakhstan/ or kenya/ or kiribati/ or kosovo/ or kuwait/ or kyrgyzstan/ or laos/ or lebanon/ or liechtenstein/ or lesotho/ or liberia/ or libyan arab jamahiriya/ or madagascar/ or malawi/ or exp malaysia/ or maldives/ or mali/ or malta/ or mauritania/ or mauritius/ or melanesia/ or moldova/ or monaco/ or mongolia/ or "montenegro (republic)"/ or morocco/ or mozambique/ or myanmar/ or namibia/ or nauru/ or nepal/ or nicaragua/ or niger/ or nigeria/ or niue/ or north africa/ or oman/ or exp pakistan/ or palau/ or palestine/ or panama/ or papua new guinea/ or paraguay/ or peru/ or philippines/ or polynesia/ or qatar/ or "republic of north macedonia"/ or romania/ or exp russian federation/ or rwanda/ or sahel/ or "saint kitts and nevis"/ or "saint lucia"/ or "saint vincent and the grenadines"/ or saudi arabia/ or senegal/ or exp serbia/ or seychelles/ or sierra leone/ or singapore/ or "sao tome and principe"/ or solomon islands/ or exp somalia/ or south africa/ or south asia/ or south sudan/ or exp southeast asia/ or sri lanka/ or sudan/ or suriname/ or syrian arab republic/ or taiwan/ or tajikistan/ or tanzania/ or thailand/ or timor-leste/ or togo/ or tonga/ or "trinidad andtobago"/ or tunisia/ or turkmenistan/ or tuvalu/ or uganda/ or exp ukraine/ or exp united arab emirates/ or uruguay/ or exp uzbekistan/ or vanuatu/ or venezuela/ or viet nam/ or western sahara/ or yemen/ or zambia/ or zimbabwe/ 1583231

88 "organisation for economic co-operation and development"/ 1912

89 exp australia/ or "australia and new zealand"/ or austria/ or baltic states/ or exp belgium/ or exp canada/ or chile/ or colombia/ or costa rica/ or czech republic/ or denmark/ or estonia/ or europe/ or exp finland/ or exp france/ or exp germany/ or greece/ or hungary/ or iceland/ or ireland/ or israel/ or exp italy/ or japan/ or korea/ or latvia/ or lithuania/ or luxembourg/ or exp mexico/ or netherlands/ or new zealand/ or north america/ or exp norway/ or poland/ or exp portugal/ or scandinavia/ or sweden/ or slovakia/ or slovenia/ or south korea/ or exp spain/ or switzerland/ or exp united kingdom/ or "turkey (republic)"/ or exp united states/ or western europe/ 3795776

90 european union/ 29352

91 developed country/ 34992

92 or/88-91 3826946

93 87 not 92 1435767

94 86 not 93 [non OECD countries removed] 1504

95 exp juvenile/ not exp adult/ 2726374

96 94 not 95 [Child studies removed] 1363

97 (elder mistreat* or elder abuse* or elder neglect*).tw,kf. 2267

98 elder abuse/ 1666

99 97 or 98 2722

100 96 not 99 [Elder abuse studies removed] 1319

Ovid MEDLINE(R) ALL 1946 to February 11, 2022

Date searched: 14-02-2022

Records found: 838

1 emergency medical services/ or advanced trauma life support care/ or call centers/ or emergency medical dispatch/ or emergency medical service communication systems/ 47416

2 exp airway management/ or exp emergency treatment/ or exp ambulatory care/ or exp critical care/ or exp perioperative care/ or exp preoperative care/ 457199

3 exp emergency service, hospital/ or emergency services, psychiatric/ or hotlines/ or poison control centers/ or exp "transportation of patients"/ or triage/ or critical care nursing/ or emergency nursing/ 129355

4 (emergenc* adj5 (care or service* or health* or ill* or treat* or medic* or unit* or centre* or centre* or department* or setting*)).tw,kf. 201058

5 (acute* adj5 (care or service* or health* or ill* or treat* or medic* or unit* or centre* or centre* or department* or setting* or ward?)).tw,kf. 220841

6 (trauma* adj5 (care or service* or ill* or unit* or centre* or centre* or department*)).tw,kf. 26703

7 (ambula* adj5 (care or service* or unit* or centre* or centre* or department* or setting*)).tw,kf. 26152

8 (critical* adj2 (care or ill*)).tw,kf. 92532

9 (urgent adj3 (care or service* or medic*)).tw,kf. 5316

10 "intensive care".tw,kf. 171685

11 paramedic*.tw,kf. 9163

12 or/1-11 [Acute Care or Ambulance services] 1064466

13 agonistic behavior/ 1779

14 exp bullying/ 5625

15 problem behavior/ 3242

16 exp harassment, non-sexual/ 5708

17 sexual harassment/ 2022

18 incivility/ 234

19 Professional Misconduct/ 3434

20 Hostility/ 5399

21 bully*.tw,kf. 7062

22 harass*.tw,kf. 4657

23 intimidat*.tw,kf. 1509

24 (lateral* adj2 violence).tw,kf. 100

25 (horizontal* adj2 violence).tw,kf. 146

26 (transgressive adj3 behavio?r*).tw,kf. 39

27 (disruptive adj3 behavio?r*).tw,kf. 4367

28 (unprofessional adj3 behavio?r*).tw,kf. 400

29 (micro-aggress* or microaggress*).tw,kf. 477

30 incivil*.tw,kf. 686

31 uncivil*.tw,kf. 215

32 rude*.tw,kf. 1194

33 mistreat*.tw,kf. 2451

34 (professional* adj3 misconduct).tw,kf. 303

35 mobbing.tw,kf. 418

36 hazing.tw,kf. 84

37 (negative behavio* or negative act?).tw,kf. 1716

38 (gaslight* or malic*).tw,kf. 8372

39 (Hidden adj5 (aggressi* or abus* or violenc*)).tw,kf. 195

40 or/13-39 [Unprofessional behaviours] 47372

41 exp Health Personnel/ 572761

42 exp Students, Health Occupations/ 79918

43 exp education, graduate/ or "internship and residency"/ or teaching rounds/ 94478

44 exp Interprofessional Relations/ 71806

45 exp Patient Care Team/ 71777

46 Workplace/ 26813

47 (nurs* or midwif* or midwiv*).tw,kf. 516983

48 paramedic?.tw,kf. 6046

49 (doctor? or physician? or clinician? or surgeon? or consultant?).tw,kf. 1005171

50 (student? adj2 (medic* or health* or clinic*)).tw,kf. 65931

51 intern?.tw,kf. 11187

52 resident?.tw,kf. 179417

53 (Therapist? or Pharmacist? or Optometrist? or Nutritionist? or Dentist? or Physiotherapist?).tw,kf. 132734

54 (Audiologist? or Anatomist? or Allergist? or An?esthetist? or An?esthesiologist? or Cardiologist? or Dieti#ian? or Endocrinologist? or Gastroenterologist? or GP? or Geriatrician? or Hospitalist? or Oncologist?).tw,kf. 234452

55 (Ophthalmologist? or Otolaryngologist? or Pathologist? or P?ediatrician? or Physiatrist? or Psychiatrist? or Pulmonologist? or Radiographer? or Radiologist?).tw,kf. 170783

56 medic?.tw,kf. 21509

57 assistant?.tw,kf. 28841

58 (cleaner? or ancillary or porter?).tw,kf. 24976

59 (auxillary or auxillaries or administrator? or secretary or secretaries or receptionist? or technician?).tw,kf. 41556

60 (employee? or worker? or Staff or personnel or practitioner? or professional? or workforce* or team*).tw,kf. /freq=2 414261

61 (workplace* or "work place*" or worksite* or "work site*" or "work setting*").tw,kf. 56083

62 or/41-61 [Staff] 2697074

63 40 and 62 [UB and Staff search 1] 13280

64 physician-nurse relations/ or interprofessional relations/ 54906

65 aggression/ or prejudice/ or Social Discrimination/ or ageism/ or gender equity/ or homophobia/ or exp racism/ or sexism/ or weight prejudice/ or xenophobia/ or Perceived Discrimination/ or Coercion/ 74909

66 64 and 65 [Aggression or prejudice among staff MeSH] 767

67 ((staff or employee* or work* or nurs* or doctor?) adj8 (WPV or violen*) adj5 among*).tw,kf. 562

68 ((staff or employee* or work* or nurs* or doctor?) adj8 coerci* adj5 among*).tw,kf. 13

69 ((staff or employee* or work* or nurs* or doctor?) adj8 Victim* adj5 among*).tw,kf. 109

70 ((staff or employee* or work* or nurs* or doctor?) adj8 (humiliat* or hostil*)).tw,kf. 570

71 ((staff or employee* or work* or nurs* or doctor?) adj3 undermin*).tw,kf. 337

72 ((staff or employee* or work* or nurs* or doctor?) adj6 (discrimination or discriminatory or discriminated)).tw,kf. 2538

73 "abusive supervision".tw,kf. 140

74 (workplace adj3 (conflict* or aggressi* or abus*)).tw,kf. 562

75 ((staff or employee* or work* or nurs* or doctor?) adj5 (gender adj2 (inequalit* or equalit*))).tw,kf. 210

76 ((staff or employee* or work* or nurs* or doctor?) adj5 (racism or racist or (racial adj3 abus*))).tw,kf. 331

77 ((staff or employee* or work* or nurs* or doctor?) adj5 (sexism or sexist)).tw,kf. 83

78 ((staff or employee* or work* or nurs* or doctor?) adj8 ((disabilit* or disabled) adj5 (inequalit* or equalit*))).tw,kf. 15

79 ((staff or employee* or work* or nurs* or doctor?) adj8 ableis*).tw,kf. 6

80 ((staff or employee* or nurs* or doctor?) adj1 (aggressi* or abus*)).tw,kf. 237

81 or/66-80 [UB and staff search 2] 6326

82 63 or 81 [UB among Staff final search] 18616

83 12 and 82 [UB among Staff in Acute Care] 1023

84 afghanistan/ or africa/ or africa, northern/ or africa, central/ or africa, eastern/ or "africa south of the sahara"/ or africa, southern/ or africa, western/ or albania/ or algeria/ or andorra/ or angola/ or "antigua and barbuda"/ or argentina/ or armenia/ or azerbaijan/ or bahamas/ or bahrain/ or bangladesh/ or barbados/ or belize/ or benin/ or bhutan/ or bolivia/ or borneo/ or "bosnia and herzegovina"/ or botswana/ or brazil/ or brunei/ or bulgaria/ or burkina faso/ or burundi/ or cabo verde/ or cambodia/ or cameroon/ or central african republic/ or chad/ or exp china/ or comoros/ or congo/ or cote d'ivoire/ or croatia/ or cuba/ or "democratic republic of the congo"/ or cyprus/ or djibouti/ or dominica/ or dominican republic/ or ecuador/ or egypt/ or el salvador/ or equatorial guinea/ or eritrea/ or eswatini/ or ethiopia/ or fiji/ or gabon/ or gambia/ or "georgia (republic)"/ or ghana/ or grenada/ or guatemala/ or guinea/ or guinea-bissau/ or guyana/ or haiti/ or honduras/ or independent state of samoa/ or exp india/ or indian ocean islands/ or indochina/ or indonesia/ or iran/ or iraq/ or jamaica/ or jordan/ or kazakhstan/ or kenya/ or kosovo/ or kuwait/ or kyrgyzstan/ or laos/ or lebanon/ or liechtenstein/ or lesotho/ or liberia/ or libya/ or madagascar/ or malaysia/ or malawi/ or mali/ or malta/ or mauritania/ or mauritius/ or mekong valley/ or melanesia/ or micronesia/ or monaco/ or mongolia/ or montenegro/ or morocco/ or mozambique/ or myanmar/ or namibia/ or nepal/ or nicaragua/ or niger/ or nigeria/ or oman/ or pakistan/ or palau/ or exp panama/ or papua new guinea/ or paraguay/ or peru/ or philippines/ or qatar/ or "republic of belarus"/ or "republic of north macedonia"/ or romania/ or exp russia/ or rwanda/ or "saint kitts and nevis"/ or saint lucia/ or "saint vincent and the grenadines"/ or "sao tome and principe"/ or saudi arabia/ or serbia/ or sierra leone/ or senegal/ or seychelles/ or singapore/ or somalia/ or south africa/ or south sudan/ or sri lanka/ or sudan/ or suriname/ or syria/ or taiwan/ or tajikistan/ or tanzania/ or thailand/ or timor-leste/ or togo/ or tonga/ or "trinidad and tobago"/ or tunisia/ or turkmenistan/ or uganda/ or ukraine/ or united arab emirates/ or uruguay/ or uzbekistan/ or vanuatu/ or venezuela/ or vietnam/ or west indies/ or yemen/ or zambia/ or zimbabwe/ 1199047

85 "Organisation for Economic Co-Operation and Development"/ 415

86 australasia/ or exp australia/ or austria/ or baltic states/ or belgium/ or exp canada/ or chile/ or colombia/ or costa rica/ or czech republic/ or exp denmark/ or estonia/ or europe/ or finland/ or exp france/ or exp germany/ or greece/ or hungary/ or iceland/ or ireland/ or israel/ or exp italy/ or exp japan/ or korea/ or latvia/ or lithuania/ or luxembourg/ or mexico/ or netherlands/ or new zealand/ or north america/ or exp norway/ or poland/ or portugal/ or exp "republic of korea"/ or "scandinavian and nordic countries"/ or slovakia/ or slovenia/ or spain/ or sweden/ or switzerland/ or turkey/ or exp united kingdom/ or exp united states/ 3382217

87 European Union/ 17106

88 Developed Countries/ 21067

89 or/85-88 3397472

90 84 not 89 [OECD search filter NICE 2021] 1112292

91 83 not 90 [UB among Acute Care Staff with non-OECD countries removed] 931

92 (exp Child/ or Adolescent/ or exp Infant/) not exp Adult/ 2021427

93 91 not 92 [Child studies removed] 863

94 (elder mistreat* or elder abuse* or elder neglect*).tw,kf. 1880

95 93 not 94 [Elder abuse studies removed] 838

HMIC Health Management Information Consortium (Ovid) 1979 to November 2021

Date searched: 14-02-2022

Records found: 58

1 exp emergency health services/ 3422

2 exp emergency treatment/ 872

3 hospital departments/ or exp accident & emergency departments/ or exp acute units/ or exp critical care units/ or exp intensive care units/ or exp pre operative units/ or exp trauma centres/ 1963

4 accident & emergency nursing/ or ambulance service nursing/ or intensive care nursing/ 149

5 accident & emergency nursing/ or ambulance service nursing/ or intensive care nursing/ or triage/ 442

6 (acute* adj5 (care or service* or health* or ill* or treat* or medic* or unit* or centre* or centre* or department* or setting* or ward?)).tw,hw. 6620

7 (trauma* adj5 (care or service* or ill* or unit* or centre* or centre* or department*)).tw,hw. 440

8 (ambula* adj5 (care or service* or unit* or centre* or centre* or department* or setting*)).tw,hw. 2109

9 (critical* adj2 (care or ill*)).tw,hw. 1076

10 (urgent adj3 (care or service* or medic*)).tw,hw. 455

11 "intensive care".tw,hw. 2518

12 paramedic*.tw,hw. 484

13 or/1-12 [Acute Care or Ambulance services] 15204

14 bullying/ 319

15 exp harassment/ 488

16 malpractice/ 276

17 hostility/ 12

18 exp discrimination/ 2100

19 bully*.tw,hw. 430

20 harass*.tw,hw. 435

21 intimidat*.tw,hw. 107

22 (lateral* adj2 violence).tw,hw. 2

23 (horizontal* adj2 violence).tw,hw. 6

24 (transgressive adj3 behavio?r*).tw,hw. 0

25 (disruptive adj3 behavio?r*).tw,hw. 81

26 (unprofessional adj3 behavio?r*).tw,hw. 11

27 (micro-aggress* or microaggress*).tw,hw. 2

28 incivil*.tw,hw. 17

29 uncivil*.tw,hw. 4

30 rude*.tw,hw. 29

31 mistreat*.tw,hw. 51

32 (professional* adj3 misconduct).tw,hw. 31

33 mobbing.tw,hw. 2

34 (negative behavio* or negative act?).tw,hw. 34

35 hazing.tw,kf. 0

36 (gaslight* or malic*).tw,hw. 17

37 (Hidden adj5 (aggressi* or abus* or violenc*)).tw,hw. 18

38 or/14-37 [Unprofessional behaviours] 3329

39 exp health service staff/ 55488

40 exp medical education/ 3378

41 exp interprofessional relations/ 2548

42 exp teamwork/ 1159

43 workplace/ 80

44 (nurs* or midwif* or midwiv*).tw,hw. 48975

45 paramedic?.tw,hw. 270

46 (doctor? or physician? or clinician? or surgeon? or consultant?).tw,hw. 31472

47 (student? adj2 (medic* or health* or clinic*)).tw,hw. 1471

48 intern?.tw,hw. 61

49 resident?.tw,hw. 4966

50 (Therapist? or Pharmacist? or Optometrist? or Nutritionist? or Dentist? or Physiotherapist?).tw,hw. 8803

51 (Audiologist? or Anatomist? or Allergist? or An?esthetist? or An?esthesiologist? or Cardiologist? or Dieti#ian? or Endocrinologist? or Gastroenterologist? or GP? or Geriatrician? or Hospitalist? or Oncologist?).tw,hw. 14609

52 (Ophthalmologist? or Otolaryngologist? or Pathologist? or P?ediatrician? or Physiatrist? or Psychiatrist? or Pulmonologist? or Radiographer? or Radiologist?).tw,hw. 2785

53 medic?.tw,hw. 275

54 assistant?.tw,hw. 2110

55 (cleaner? or ancillary or porter?).tw,hw. 681

56 (auxillary or auxillaries or administrator? or secretary or secretaries or receptionist? or technician?).tw,hw. 4510

57 (employee? or worker? or Staff or personnel or practitioner? or professional? or workforce* or team*).tw,hw. 115226

58 (workplace* or "work place*" or worksite* or "work site*" or "work setting*").tw,hw. 3499

59 or/39-58 [Staff] 166259

60 38 and 59 [UB and Staff search 1] 1743

61 exp aggressive behaviour/ and (nurse doctor relations/ or interprofessional relations/) 3

62 exp prejudice/ and (nurse doctor relations/ or interprofessional relations/) 2

63 ((staff or employee* or work* or nurs* or doctor?) adj8 (WPV or violen*) adj5 among*).tw,hw. 15

64 ((staff or employee* or work* or nurs* or doctor?) adj8 Victim* adj5 among*).tw,hw. 0

65 ((staff or employee* or work* or nurs* or doctor?) adj8 (humiliat* or hostil*)).tw,hw. 76

66 ((staff or employee* or work* or nurs* or doctor?) adj3 undermin*).tw,hw. 71

67 ((staff or employee* or work* or nurs* or doctor?) adj6 (discrimination or discriminatory or discriminated)).tw,hw. 364

68 "abusive supervision".tw,hw. 0

69 (workplace adj3 (conflict* or aggressi* or abus*)).tw,hw. 35

70 ((staff or employee* or work* or nurs* or doctor?) adj5 (gender adj2 (inequalit* or equalit*))).tw,hw. 32

71 ((staff or employee* or work* or nurs* or doctor?) adj5 (racism or racist or (racial adj3 abus*))).tw,hw. 167

72 ((staff or employee* or work* or nurs* or doctor?) adj5 (sexism or sexist)).tw,hw. 18

73 ((staff or employee* or work* or nurs* or doctor?) adj8 ((disabilit* or disabled) adj5 (inequalit* or equalit*))).tw,hw. 18

74 ((staff or employee* or work* or nurs* or doctor?) adj8 ableis*).tw,hw. 0

75 ((staff or employee* or work* or nurs* or doctor?) adj8 coerci* adj5 among*).tw,hw. 1

76 ((staff or employee* or nurs* or doctor?) adj1 (aggressi* or abus*)).tw,hw. 31

77 or/61-76 [UB and staff search 2] 800

78 60 or 77 [UB and Staff final search] 2224

79 78 and 13 [UB among Acute Care Staff] 59

80 ((child* or infant* or baby or babies or preterm* or adolesc* or newborn* or pediatric* or paediatri* or neonate* or teen* or schoolchild*) not (adult or elderly or geriatric*)).ti. 21099

81 79 not 80 [Child studies removed] 58

82 (elder mistreat* or elder abuse* or elder neglect*).tw,hw. 301

83 81 not 82 [Elder abuse studies removed] 58

NICE Evidence Search <https://www.evidence.nhs.uk/>

Date searched: 15-02-2022

Records found: 79

("workplace bullying" or "workplace discrimination" or "unprofessional behaviour" or "staff bullying" or "bullying staff" or "staff harassment" or "staff discrimination") and (health or healthcare or hospital or medicine or medical or nurse or doctor) limited to filters

Filter applied: Area of interest : Clinical OR Area of interest: Commissioning and Management

Patient Safety Network <https://psnet.ahrq.gov/>

Date searched: 15-02-2022

Records found: 83

abuse bullying unprofessional harassment discrimination microaggression incivility intimidation humiliation hostility mobbing hazing gaslighting malicious

Limited to Ambulatory Care setting OR Limit to Hospital setting

#### 3. USA update search Aug 2022

Sources searched:

- CINAHL (EBSCOhost)
- Embase Classic+Embase (Ovid) 1947 to 2022 August 24
- Ovid MEDLINE(R) ALL 1946 to August 24, 2022
- Google Scholar
- Patient Safety Network <https://psnet.ahrq.gov/>

CINAHL (EBSCOhost)

Date searched: 25-08-2022

Records found: 357

# Query Results

S39 S37 AND S38 357

S38 (MH "United States+") OR AF ( USA or "united states" or america* ) OR TI ( USA or "united states" or america* ) OR AB ( USA or "united states" or america* ) 1,752,918

S37 S35 NOT S36 1,316

S36 TX ( ("elder mistreat*" or "elder abuse*" or "elder neglect*") ) 4,919

S35 S33 NOT S34 1,360

S34 ( ( (MH "Child") OR (MH "Adolescence+") OR (MH "Minors (Legal)") ) NOT (MH "Adult+") ) 483,829

S33 S22 NOT S32 1,436

S32 S28 NOT S31 381,030

S31 S29 OR S30 1,714,259

S30 (MH "Australia+") OR (MH "Europe") OR (MH "Austria") OR (MH "Baltic States+") OR (MH "Belgium") OR (MH "Canada+") OR (MH "Chile") OR (MH "Colombia") OR (MH "Costa Rica") OR (MH "Czech Republic") OR (MH "Scandinavia+") OR (MH "France") OR (MH "Germany+") OR (MH "Greece") OR (MH "Hungary") OR (MH "Iceland") OR (MH "Ireland") OR (MH "Italy") OR (MH "Israel") OR (MH "Japan") OR (MH "South Korea") OR (MH "Luxembourg") OR (MH "Mexico") OR (MH "Netherlands") OR (MH "New Zealand") OR (MH "North America") OR (MH "Poland") OR (MH "Portugal") OR (MH "Slovakia") OR (MH "Slovenia") OR (MH "Spain") OR (MH "Switzerland") OR (MH "Turkey") OR (MH "United Kingdom+") OR (MH "United States+") 1,707,539

S29 (MH "Developed Countries") or (MH "European Union") or (MH "Organisation for Economic Co-Operation and Development") 10,460

S28 (S23 OR S24 OR S25 OR S26 OR S27) 419,922

S27 (MH "Africa+") 95,616

S26 (MH "Asia, Southeastern+") OR (MH "China+") OR (MH "Hong Kong") OR (MH "Macao") OR (MH "Mongolia") OR (MH "North Korea") OR (MH "Taiwan") OR (MH "Atlantic Islands") OR (MH "Indian Ocean Islands+") OR (MH "Melanesia+") OR (MH "Micronesia+") OR (MH "Polynesia+") 132,915

S25 (MH "Bangladesh") OR (MH "Bhutan") OR (MH "India") OR (MH "Yemen") OR (MH "United Arab Emirates") OR (MH "Syria") OR (MH "Saudi Arabia") OR (MH "Qatar") OR (MH "Oman") OR (MH "Lebanon") OR (MH "Kuwait") OR (MH "Jordan") OR (MH "Iraq") OR (MH "Iran") OR (MH "Bahrain") OR (MH "Afghanistan") OR (MH "Nepal") OR (MH "Pakistan") OR (MH "Sri Lanka") OR (MH "Asia, Central+") 113,865

S24 (MH "Argentina") OR (MH "Bolivia") OR (MH "Brazil") OR (MH "Ecuador") OR (MH "French Guiana") OR (MH "Guyana") OR (MH "Paraguay") OR (MH "Peru") OR (MH "Suriname") OR (MH "Uruguay") OR (MH "Venezuela") OR (MH "Belize") OR (MH "El Salvador") OR (MH "Guatemala") OR (MH "Honduras") OR (MH "Nicaragua") OR (MH "Panama+") or (MH "West Indies+") 74,518

S23 (MH "Albania") OR (MH "Andorra") OR (MH "Armenia") OR (MH "Azerbaijan") or (MH "Byelarus") OR (MH "Bosnia-Herzegovina") OR (MH "Croatia") OR (MH "Bulgaria") OR (MH "Georgia (Republic)") OR (MH "Gibraltar") OR (MH "Liechtenstein") OR (MH "Macedonia (Republic)") OR (MH "Moldova") OR (MH "Monaco") OR (MH "Romania") OR (MH "Russia") OR (MH "San Marino") OR (MH "Serbia") OR (MH "Ukraine") OR (MH "Yugoslavia") 12,299

S22 S5 AND S21 1,557

S21 S16 OR S17 OR S18 OR S19 OR S20 24,298

S20 TI ( (staff or employee* or work* or nurs* or doctor#) n5 (sexism or sexist or ableis* or racism or racist or (racial n3 abus*)) ) OR AB ( (staff or employee* or work* or nurs* or doctor#) n5 (sexism or sexist or ableis* or racism or racist or (racial n3 abus*)) ) OR SU ( (staff or employee* or work* or nurs* or doctor#) n5 (sexism or sexist or ableis* or racism or racist or (racial n3 abus*)) ) 732

S19 TI ( ((staff or employee* or work* or nurs* or doctor#) n6 ((gender or disabilit* or disabled) n4 (inequalit* or equalit*))) OR ((staff or employee* or nurs* or doctor?) n1 (aggressi* or abus*))) OR AB ( ((staff or employee* or work* or nurs* or doctor#) n6 ((gender or disabilit* or disabled) n4 (inequalit* or equalit*))) OR ((staff or employee* or nurs* or doctor?) n1 (aggressi* or abus*)) ) OR SU ( ((staff or employee* or work* or nurs* or doctor#) n6 ((gender or disabilit* or disabled) n4 (inequalit* or equalit*))) OR ((staff or employee* or nurs* or doctor?) n1 (aggressi* or abus*)) ) 2,017

S18 ((MH "Workplace Violence") or (MH "Aggression") or (MH "Prejudice+") or (MH "Discrimination+") or (MH "Dehumanization") OR (MH "Oppressed Group Behavior") OR (MH "Coercion")) AND ( (MH "Interprofessional Relations+") or (MH "Intraprofessional Relations")) 1,053

S17 TI ( ((staff or employee* or work* or nurs* or doctor#) n4 (hostil* or undermin* or discrimination or discriminatory or discriminated or humiliat*) ) OR ((staff or employee* or work* or nurs* or doctor#) n4 (WPV or violen* or victim* or coerci*) n3 among*) OR "abusive supervision" OR (workplace n2 (conflict* or aggressi* or abus*)) ) OR AB ( ((staff or employee* or work* or nurs* or doctor#) n4 (hostil* or undermin* or discrimination or discriminatory or discriminated or humiliat*) ) OR ((staff or employee* or work* or nurs* or doctor#) n4 (WPV or violen* or victim* or coerci*) n3 among*) OR "abusive supervision" OR (workplace n2 (conflict* or aggressi* or abus*)) ) OR SU ( ((staff or employee* or work* or nurs* or doctor#) n4 (hostil* or undermin* or discrimination or discriminatory or discriminated or humiliat*) ) OR ((staff or employee* or work* or nurs* or doctor#) n4 (WPV or violen* or victim* or coerci*) n3 among*) OR "abusive supervision" OR (workplace n2 (conflict* or aggressi* or abus*)) ) 3,244

S16 S9 AND S15 18,721

S15 S10 OR S11 OR S12 OR S13 OR S14 2,270,521

S14 (MH "Health Personnel+") or (MH "Students, Health Occupations+") or (MH "Internship and Residency") OR (MH "Education, Graduate") OR (MH "Teamwork") 712,763

S13 TI ( (student# n2 (health* or clinic* or medic*)) OR employee# or worker# or Staff or personnel or practitioner# or professional# or workforce* or workplace* or "work place*" or worksite* or "work site*" or "work setting*" OR team*) OR AB ( (student# n2 (health* or clinic* or medic*)) OR employee# or worker# or Staff or personnel or practitioner# or professional# or workforce* or workplace* or "work place*" or worksite* or "work site*" or "work setting*" OR team*) OR SU ( (student# n2 (health* or clinic* or medic*)) OR employee# or worker# or Staff or personnel or practitioner# or professional# or workforce* or workplace* or "work place*" or worksite* or "work site*" or "work setting*" OR team*) 1,118,297

S12 TI ( Pathologist# or P#ediatrician# or Physiatrist# or Psychiatrist# or Pulmonologist# or Radiographer# or Radiologist# OR assistant# or cleaner# or ancillary or porter# or auxillary or auxillaries or administrator# or secretary or secretaries or receptionist# or technician# ) OR AB ( Pathologist# or P#ediatrician# or Physiatrist# or Psychiatrist# or Pulmonologist# or Radiographer# or Radiologist# OR assistant# or cleaner# or ancillary or porter# or auxillary or auxillaries or administrator# or secretary or secretaries or receptionist# or technician# ) OR SU ( Pathologist# or P#ediatrician# or Physiatrist# or Psychiatrist# or Pulmonologist# or Radiographer# or Radiologist# OR assistant# or cleaner# or ancillary or porter# or auxillary or auxillaries or administrator# or secretary or secretaries or receptionist# or technician# ) 157,381

S11 TI ( Audiologist# or Anatomist# or Allergist# or An#esthetist# or An#esthesiologist# or Cardiologist# or Dieti#ian# or Endocrinologist# or Gastroenterologist# or GP# or Geriatrician# or Hospitalist# or Oncologist# OR Ophthalmologist# or Otolaryngologist# ) OR AB ( Audiologist# or Anatomist# or Allergist# or An#esthetist# or An#esthesiologist# or Cardiologist# or Dieti#ian# or Endocrinologist# or Gastroenterologist# or GP# or Geriatrician# or Hospitalist# or Oncologist# OR Ophthalmologist# or Otolaryngologist# ) OR SU ( Audiologist# or Anatomist# or Allergist# or An#esthetist# or An#esthesiologist# or Cardiologist# or Dieti#ian# or Endocrinologist# or Gastroenterologist# or GP# or Geriatrician# or Hospitalist# or Oncologist# OR Ophthalmologist# or Otolaryngologist# ) 88,249

S10 TI ( ( nurs* or midwif* or midwiv or paramedic# or doctor# or physician# or clinician# or surgeon# or consultant# OR medic# or intern# or resident# or Therapist# or Pharmacist# or Optometrist# or Nutritionist# or Dentist# or Physiotherapist# ) ) OR AB ( ( nurs* or midwif* or midwiv or paramedic# or doctor# or physician# or clinician# or surgeon# or consultant# OR medic# or intern# or resident# or Therapist# or Pharmacist# or Optometrist# or Nutritionist# or Dentist# or Physiotherapist# ) ) OR SU ( ( nurs* or midwif* or midwiv or paramedic# or doctor# or physician# or clinician# or surgeon# or consultant# OR medic# or intern# or resident# or Therapist# or Pharmacist# or Optometrist# or Nutritionist# or Dentist# or Physiotherapist# ) ) 1,532,802

S9 S6 OR S7 OR S8 32,395

S8 TI ( ( ((lateral* or horizontal*) n2 violence) ) OR ( ((transgressive or disruptive or unprofessional) n2 behavio#r*) ) OR (professional* n2 misconduct) OR ( "negative behavio*" or "negative act" ) ) OR AB ( ( ((lateral* or horizontal*) n2 violence) ) OR ( ((transgressive or disruptive or unprofessional) n2 behavio#r*) ) OR (professional* n2 misconduct) OR ( "negative behavio*" or "negative act" ) ) OR SU ( ( ((lateral* or horizontal*) n2 violence) ) OR ( ((transgressive or disruptive or unprofessional) n2 behavio#r*) ) OR (professional* n2 misconduct) OR ( "negative behavio*" or "negative act" ) ) 12,059

S7 TI ( bully* or harass* or intimidat* or micro-aggress* or microaggress* or incivil* or uncivil* or rude* or mistreat* or mobbing or hazing or gaslight* or malic* or (Hidden n5 (aggressi* or abus* or violenc*))) OR AB ( bully* or harass* or intimidat* or micro-aggress* or microaggress* or incivil* or uncivil* or rude* or mistreat* or mobbing or hazing or gaslight* or malic* or (Hidden n5 (aggressi* or abus* or violenc*))) OR SU ( bully* or harass* or intimidat* or micro-aggress* or microaggress* or incivil* or uncivil* or rude* or mistreat* or mobbing or hazing or gaslight* or malic* or (Hidden n5 (aggressi* or abus* or violenc*))) 19,245

S6 (MH "Disruptive Behavior") or (MH "Bullying+") OR (MH "Emotional Abuse") OR (MH "Verbal Abuse") or (MH "Sexual Harassment") or (MH "Professional Misconduct") or (MH "Incivility") or (MH "Scapegoating") 22,843

S5 S1 OR S2 OR S3 OR S4 520,545

S4 TI ( ( (critical* n2 (care or ill*)) ) OR ( (urgent n2 (care or service* or medic*)) ) OR "intensive care" OR paramedic*) OR AB ( ( (critical* n2 (care or ill*)) ) OR ( (urgent n2 (care or service* or medic*)) ) OR "intensive care" OR paramedic*) OR SU ( ( (critical* n2 (care or ill*)) ) OR ( (urgent n2 (care or service* or medic*)) ) OR "intensive care" OR paramedic*) 170,862

S3 TI ( ( ((trauma* or ambulan*) n4 (care or service* or ill* or unit* or centre* or centre* or department* or setting)) ) ) OR AB ( ( ((trauma* or ambulan*) n4 (care or service* or ill* or unit* or centre* or centre* or department* or setting)) ) ) OR SU ( ( ((trauma* or ambulan*) n4 (care or service* or ill* or unit* or centre* or centre* or department* or setting)) ) ) 19,577

S2 TI ( ( ((emergenc* or acute*) n4 (care or service* or health* or ill* or treat* or medic* or unit* or centre* or centre* or department* or setting* or ward#)) ) ) OR AB ( ( ((emergenc* or acute*) n4 (care or service* or health* or ill* or treat* or medic* or unit* or centre* or centre* or department* or setting* or ward#)) ) ) OR SU ( ( ((emergenc* or acute*) n4 (care or service* or health* or ill* or treat* or medic* or unit* or centre* or centre* or department* or setting* or ward#)) ) ) 254,587

S1 (MH "Emergency Medical Services+") or (MH "Emergency Treatment+") or (MH "Emergency Care") or (MH "Airway Management+") or (MH "Ambulatory Care") OR (MH "Acute Care") or (MH "Critical Care+") OR (MH "Perioperative Care") OR (MH "Preoperative Care+") or (MH "Critical Care Nursing+") or (MH "Emergency Nursing+") 310,494

Embase Classic+Embase (Ovid) 1947 to 2022 August 24

Date searched: 25-08-2022

Records found: 585

1 emergency health service/ or emergency medical dispatch/ or hospital emergency service/ or psychiatric emergency service/ (122336)

2 emergency treatment/ or evidence based emergency medicine/ (18782)

3 emergency care/ or advanced trauma life support/ or emergency ward/ (233211)

4 respiration control/ or exp assisted ventilation/ or exp artificial ventilation/ (319367)

5 exp ambulatory care/ or exp intensive care/ (850847)

6 perioperative nursing/ or exp perioperative period/ or exp preoperative care/ (116685)

7 exp hotline/ or poison center/ or exp ambulance/ (23525)

8 exp intensive care nursing/ or emergency nursing/ (9995)

9 (emergenc* adj5 (care or service* or health* or ill* or treat* or medic* or unit* or centre* or centre* or department* or setting*)).tw,kf. (311715)

10 (acute* adj5 (care or service* or health* or ill* or treat* or medic* or unit* or centre* or centre* or department* or setting* or ward?)).tw,kf. (343867)

11 (trauma* adj5 (care or service* or ill* or unit* or centre* or centre* or department*)).tw,kf. (38174)

12 (ambula* adj5 (care or service* or unit* or centre* or centre* or department* or setting*)).tw,kf. (39628)

13 (critical* adj2 (care or ill*)).tw,kf. (152661)

14 (urgent adj3 (care or service* or medic*)).tw,kf. (8970)

15 "intensive care".tw,kf. (267368)

16 paramedic*.tw,kf. (14376)

17 or/1-16 [Acute Care or Ambulance services] (1915208)

18 agonistic behavior/ (215)

19 exp bullying/ (9865)

20 disruptive behavior/ (3294)

21 harassment/ or non-sexual harassment/ or exp online harassment/ or exp sexual harassment/ (5440)

22 incivility/ (409)

23 microaggression/ (260)

24 professional misconduct/ (3824)

25 exp hostility/ (14620)

26 bully*.tw,kf. (9103)

27 harass*.tw,kf. (5669)

28 intimidat*.tw,kf. (2045)

29 (lateral* adj2 violence).tw,kf. (119)

30 (horizontal* adj2 violence).tw,kf. (156)

31 (transgressive adj3 behavio?r*).tw,kf. (48)

32 (disruptive adj3 behavio?r*).tw,kf. (5760)

33 (unprofessional adj3 behavio?r*).tw,kf. (511)

34 (micro-aggress* or microaggress*).tw,kf. (672)

35 incivil*.tw,kf. (735)

36 uncivil*.tw,kf. (228)

37 rude*.tw,kf. (1347)

38 mistreat*.tw,kf. (3323)

39 (professional* adj3 misconduct).tw,kf. (367)

40 mobbing.tw,kf. (536)

41 (negative behavio* or negative act?).tw,kf. (2165)

42 hazing.tw,kf. (104)

43 (gaslight* or malic*).tw,kf. (10258)

44 (Hidden adj5 (aggressi* or abus* or violenc*)).tw,kf. (257)

45 or/18-44 [Unprofessional behaviours] (63710)

46 exp health care personnel/ (1900535)

47 exp health student/ (135620)

48 exp medical education/ (375149)

49 public relations/ (63614)

50 workplace/ (50638)

51 teamwork/ (20829)

52 (nurs* or midwif* or midwiv*).tw,kf. (637750)

53 paramedic?.tw,kf. (9666)

54 (doctor? or physician? or clinician? or surgeon? or consultant?).tw,kf. (1549923)

55 (student? adj2 (medic* or health* or clinic*)).tw,kf. (93266)

56 intern?.tw,kf. (16967)

57 resident?.tw,kf. (250684)

58 (Therapist? or Pharmacist? or Optometrist? or Nutritionist? or Dentist? or Physiotherapist?).tw,kf. (226034)

59 (Audiologist? or Anatomist? or Allergist? or An?esthetist? or An?esthesiologist? or Cardiologist? or Dieti#ian? or Endocrinologist? or Gastroenterologist? or GP? or Geriatrician? or Hospitalist? or Oncologist?).tw,kf. (376178)

60 (Ophthalmologist? or Otolaryngologist? or Pathologist? or P?ediatrician? or Physiatrist? or Psychiatrist? or Pulmonologist? or Radiographer? or Radiologist?).tw,kf. (291043)

61 medic?.tw,kf. (42867)

62 assistant?.tw,kf. (43633)

63 (cleaner? or ancillary or porter?).tw,kf. (36354)

64 (auxillary or auxillaries or administrator? or secretary or secretaries or receptionist? or technician?).tw,kf. (58834)

65 (employee? or worker? or staff or personnel or practitioner? or professional? or workforce* or team*).tw,kf. /freq=2 (600934)

66 (workplace* or "work place*" or worksite* or "work site*" or "work setting*").tw,kf. (73577)

67 or/46-66 [Staff] (4249002)

68 45 and 67 [UB and Staff search 1] (18910)

69 public relations/ and (aggression/ or aggressiveness/ or prejudice/ or exp social discrimination/ or coercion/) (419)

70 ((staff or employee* or work* or nurs* or doctor?) adj8 (WPV or violen*) adj5 among*).tw,kf. (649)

71 ((staff or employee* or work* or nurs* or doctor?) adj8 Victim* adj5 among*).tw,kf. (142)

72 ((staff or employee* or work* or nurs* or doctor?) adj8 (humiliat* or hostil*)).tw,kf. (765)

73 ((staff or employee* or work* or nurs* or doctor?) adj3 undermin*).tw,kf. (389)

74 ((staff or employee* or work* or nurs* or doctor?) adj6 (discrimination or discriminatory or discriminated)).tw,kf. (3096)

75 "abusive supervision".tw,kf. (128)

76 (workplace adj3 (conflict* or aggressi* or abus*)).tw,kf. (668)

77 ((staff or employee* or work* or nurs* or doctor?) adj5 (gender adj2 (inequalit* or equalit*))).tw,kf. (207)

78 ((staff or employee* or work* or nurs* or doctor?) adj5 (racism or racist or (racial adj3 abus*))).tw,kf. (421)

79 ((staff or employee* or work* or nurs* or doctor?) adj5 (sexism or sexist)).tw,kf. (83)

80 ((staff or employee* or work* or nurs* or doctor?) adj8 ((disabilit* or disabled) adj5 (inequalit* or equalit*))).tw,kf. (24)

81 ((staff or employee* or work* or nurs* or doctor?) adj8 ableis*).tw,kf. (15)

82 ((staff or employee* or work* or nurs* or doctor?) adj8 coerci* adj5 among*).tw,kf. (12)

83 ((staff or employee* or nurs* or doctor?) adj1 (aggressi* or abus*)).tw,kf. (282)

84 or/69-83 [UB and staff search 2] (7035)

85 68 or 84 [UB among Staff final search] (24790)

86 17 and 85 [UB among Staff in Acute Care] (1767)

87 afghanistan/ or africa/ or "africa south of the sahara"/ or albania/ or algeria/ or andorra/ or angola/ or argentina/ or "antigua and barbuda"/ or armenia/ or exp azerbaijan/ or bahamas/ or bahrain/ or bangladesh/ or barbados/ or belarus/ or belize/ or benin/ or bhutan/ or bolivia/ or borneo/ or exp "bosnia and herzegovina"/ or botswana/ or exp brazil/ or brunei darussalam/ or bulgaria/ or burkina faso/ or burundi/ or cambodia/ or cameroon/ or cape verde/ or central africa/ or central african republic/ or chad/ or exp china/ or comoros/ or congo/ or cook islands/ or coted'ivoire/ or croatia/ or cuba/ or cyprus/ or democratic republic congo/ or djibouti/ or dominica/ or dominican republic/ or ecuador/ or el salvador/ or egypt/ or equatorial guinea/ or eritrea/ or eswatini/ or ethiopia/ or exp "federated states of micronesia"/ or fiji/ or gabon/ or gambia/ or exp "georgia (republic)"/ or ghana/ or grenada/ or guatemala/ or guinea/ or guinea-bissau/ or guyana/ or haiti/ or honduras/ or exp india/ or exp indonesia/ or iran/ or exp iraq/ or jamaica/ or jordan/ or kazakhstan/ or kenya/ or kiribati/ or kosovo/ or kuwait/ or kyrgyzstan/ or laos/ or lebanon/ or liechtenstein/ or lesotho/ or liberia/ or libyan arab jamahiriya/ or madagascar/ or malawi/ or exp malaysia/ or maldives/ or mali/ or malta/ or mauritania/ or mauritius/ or melanesia/ or moldova/ or monaco/ or mongolia/ or "montenegro (republic)"/ or morocco/ or mozambique/ or myanmar/ or namibia/ or nauru/ or nepal/ or nicaragua/ or niger/ or nigeria/ or niue/ or north africa/ or oman/ or exp pakistan/ or palau/ or palestine/ or panama/ or papua new guinea/ or paraguay/ or peru/ or philippines/ or polynesia/ or qatar/ or "republic of north macedonia"/ or romania/ or exp russian federation/ or rwanda/ or sahel/ or "saint kitts and nevis"/ or "saint lucia"/ or "saint vincent and the grenadines"/ or saudi arabia/ or senegal/ or exp serbia/ or seychelles/ or sierra leone/ or singapore/ or "sao tome and principe"/ or solomon islands/ or exp somalia/ or south africa/ or south asia/ or south sudan/ or exp southeast asia/ or sri lanka/ or sudan/ or suriname/ or syrian arab republic/ or taiwan/ or tajikistan/ or tanzania/ or thailand/ or timor-leste/ or togo/ or tonga/ or "trinidad andtobago"/ or tunisia/ or turkmenistan/ or tuvalu/ or uganda/ or exp ukraine/ or exp united arab emirates/ or uruguay/ or exp uzbekistan/ or vanuatu/ or venezuela/ or viet nam/ or western sahara/ or yemen/ or zambia/ or zimbabwe/ (1644602)

88 "organisation for economic co-operation and development"/ (2158)

89 exp australia/ or "australia and new zealand"/ or austria/ or baltic states/ or exp belgium/ or exp canada/ or chile/ or colombia/ or costa rica/ or czech republic/ or denmark/ or estonia/ or europe/ or exp finland/ or exp france/ or exp germany/ or greece/ or hungary/ or iceland/ or ireland/ or israel/ or exp italy/ or japan/ or korea/ or latvia/ or lithuania/ or luxembourg/ or exp mexico/ or netherlands/ or new zealand/ or north america/ or exp norway/ or poland/ or exp portugal/ or scandinavia/ or sweden/ or slovakia/ or slovenia/ or south korea/ or exp spain/ or switzerland/ or exp united kingdom/ or "turkey (republic)"/ or exp united states/ or western europe/ (3872230)

90 european union/ (30020)

91 developed country/ (35321)

92 or/88-91 (3904207)

93 87 not 92 (1492561)

94 86 not 93 [non OECD countries removed] (1595)

95 exp juvenile/ not exp adult/ (2786964)

96 94 not 95 [Child studies removed] (1443)

97 (elder mistreat* or elder abuse* or elder neglect*).tw,kf. (2330)

98 elder abuse/ (1733)

99 97 or 98 (2801)

100 96 not 99 [Elder abuse studies removed] (1398)

101 exp united states/ (1419795)

102 (USA or "united states" or america*).ti,ab,in,kf. (11644462)

103 101 or 102 (12262076)

104 100 and 103 (585)

Ovid MEDLINE(R) ALL 1946 to August 24, 2022

Date searched: 25-08-2022

Records found: 316

1 emergency medical services/ or advanced trauma life support care/ or call centers/ or emergency medical dispatch/ or emergency medical service communication systems/ (48547)

2 exp airway management/ or exp emergency treatment/ or exp ambulatory care/ or exp critical care/ or exp perioperative care/ or exp preoperative care/ (463588)

3 exp emergency service, hospital/ or emergency services, psychiatric/ or hotlines/ or poison control centers/ or exp "transportation of patients"/ or triage/ or critical care nursing/ or emergency nursing/ (133395)

4 (emergenc* adj5 (care or service* or health* or ill* or treat* or medic* or unit* or centre* or centre* or department* or setting*)).tw,kf. (211268)

5 (acute* adj5 (care or service* or health* or ill* or treat* or medic* or unit* or centre* or centre* or department* or setting* or ward?)).tw,kf. (228051)

6 (trauma* adj5 (care or service* or ill* or unit* or centre* or centre* or department*)).tw,kf. (27944)

7 (ambula* adj5 (care or service* or unit* or centre* or centre* or department* or setting*)).tw,kf. (26953)

8 (critical* adj2 (care or ill*)).tw,kf. (97288)

9 (urgent adj3 (care or service* or medic*)).tw,kf. (5676)

10 "intensive care".tw,kf. (180297)

11 paramedic*.tw,kf. (9528)

12 or/1-11 [Acute Care or Ambulance services] (1096671)

13 agonistic behavior/ (1788)

14 exp bullying/ (6143)

15 problem behavior/ (3530)

16 exp harassment, non-sexual/ (6227)

17 sexual harassment/ (2129)

18 incivility/ (263)

19 Professional Misconduct/ (3450)

20 Hostility/ (5459)

21 bully*.tw,kf. (7551)

22 harass*.tw,kf. (4951)

23 intimidat*.tw,kf. (1564)

24 (lateral* adj2 violence).tw,kf. (107)

25 (horizontal* adj2 violence).tw,kf. (147)

26 (transgressive adj3 behavio?r*).tw,kf. (41)

27 (disruptive adj3 behavio?r*).tw,kf. (4505)

28 (unprofessional adj3 behavio?r*).tw,kf. (424)

29 (micro-aggress* or microaggress*).tw,kf. (572)

30 incivil*.tw,kf. (734)

31 uncivil*.tw,kf. (226)

32 rude*.tw,kf. (1234)

33 mistreat*.tw,kf. (2612)

34 (professional* adj3 misconduct).tw,kf. (308)

35 mobbing.tw,kf. (426)

36 hazing.tw,kf. (91)

37 (negative behavio* or negative act?).tw,kf. (1789)

38 (gaslight* or malic*).tw,kf. (8741)

39 (Hidden adj5 (aggressi* or abus* or violenc*)).tw,kf. (205)

40 or/13-39 [Unprofessional behaviours] (49573)

41 exp Health Personnel/ (589916)

42 exp Students, Health Occupations/ (83031)

43 exp education, graduate/ or "internship and residency"/ or teaching rounds/ (96785)

44 exp Interprofessional Relations/ (72096)

45 exp Patient Care Team/ (72269)

46 Workplace/ (28057)

47 (nurs* or midwif* or midwiv*).tw,kf. (530620)

48 paramedic?.tw,kf. (6351)

49 (doctor? or physician? or clinician? or surgeon? or consultant?).tw,kf. (1040260)

50 (student? adj2 (medic* or health* or clinic*)).tw,kf. (69323)

51 intern?.tw,kf. (11676)

52 resident?.tw,kf. (187085)

53 (Therapist? or Pharmacist? or Optometrist? or Nutritionist? or Dentist? or Physiotherapist?).tw,kf. (137588)

54 (Audiologist? or Anatomist? or Allergist? or An?esthetist? or An?esthesiologist? or Cardiologist? or Dieti#ian? or Endocrinologist? or Gastroenterologist? or GP? or Geriatrician? or Hospitalist? or Oncologist?).tw,kf. (243485)

55 (Ophthalmologist? or Otolaryngologist? or Pathologist? or P?ediatrician? or Physiatrist? or Psychiatrist? or Pulmonologist? or Radiographer? or Radiologist?).tw,kf. (176891)

56 medic?.tw,kf. (22425)

57 assistant?.tw,kf. (29950)

58 (cleaner? or ancillary or porter?).tw,kf. (25972)

59 (auxillary or auxillaries or administrator? or secretary or secretaries or receptionist? or technician?).tw,kf. (42828)

60 (employee? or worker? or Staff or personnel or practitioner? or professional? or workforce* or team*).tw,kf. /freq=2 (434686)

61 (workplace* or "work place*" or worksite* or "work site*" or "work setting*").tw,kf. (58882)

62 or/41-61 [Staff] (2786785)

63 40 and 62 [UB and Staff search 1] (13814)

64 physician-nurse relations/ or interprofessional relations/ (55150)

65 aggression/ or prejudice/ or Social Discrimination/ or ageism/ or gender equity/ or homophobia/ or exp racism/ or sexism/ or weight prejudice/ or xenophobia/ or Perceived Discrimination/ or Coercion/ (77044)

66 64 and 65 [Aggression or prejudice among staff MeSH] (767)

67 ((staff or employee* or work* or nurs* or doctor?) adj8 (WPV or violen*) adj5 among*).tw,kf. (610)

68 ((staff or employee* or work* or nurs* or doctor?) adj8 coerci* adj5 among*).tw,kf. (13)

69 ((staff or employee* or work* or nurs* or doctor?) adj8 Victim* adj5 among*).tw,kf. (123)

70 ((staff or employee* or work* or nurs* or doctor?) adj8 (humiliat* or hostil*)).tw,kf. (593)

71 ((staff or employee* or work* or nurs* or doctor?) adj3 undermin*).tw,kf. (362)

72 ((staff or employee* or work* or nurs* or doctor?) adj6 (discrimination or discriminatory or discriminated)).tw,kf. (2672)

73 "abusive supervision".tw,kf. (159)

74 (workplace adj3 (conflict* or aggressi* or abus*)).tw,kf. (598)

75 ((staff or employee* or work* or nurs* or doctor?) adj5 (gender adj2 (inequalit* or equalit*))).tw,kf. (231)

76 ((staff or employee* or work* or nurs* or doctor?) adj5 (racism or racist or (racial adj3 abus*))).tw,kf. (390)

77 ((staff or employee* or work* or nurs* or doctor?) adj5 (sexism or sexist)).tw,kf. (91)

78 ((staff or employee* or work* or nurs* or doctor?) adj8 ((disabilit* or disabled) adj5 (inequalit* or equalit*))).tw,kf. (18)

79 ((staff or employee* or work* or nurs* or doctor?) adj8 ableis*).tw,kf. (9)

80 ((staff or employee* or nurs* or doctor?) adj1 (aggressi* or abus*)).tw,kf. (242)

81 or/66-80 [UB and staff search 2] (6606)

82 63 or 81 [UB among Staff final search] (19495)

83 12 and 82 [UB among Staff in Acute Care] (1074)

84 afghanistan/ or africa/ or africa, northern/ or africa, central/ or africa, eastern/ or "africa south of the sahara"/ or africa, southern/ or africa, western/ or albania/ or algeria/ or andorra/ or angola/ or "antigua and barbuda"/ or argentina/ or armenia/ or azerbaijan/ or bahamas/ or bahrain/ or bangladesh/ or barbados/ or belize/ or benin/ or bhutan/ or bolivia/ or borneo/ or "bosnia and herzegovina"/ or botswana/ or brazil/ or brunei/ or bulgaria/ or burkina faso/ or burundi/ or cabo verde/ or cambodia/ or cameroon/ or central african republic/ or chad/ or exp china/ or comoros/ or congo/ or cote d'ivoire/ or croatia/ or cuba/ or "democratic republic of the congo"/ or cyprus/ or djibouti/ or dominica/ or dominican republic/ or ecuador/ or egypt/ or el salvador/ or equatorial guinea/ or eritrea/ or eswatini/ or ethiopia/ or fiji/ or gabon/ or gambia/ or "georgia (republic)"/ or ghana/ or grenada/ or guatemala/ or guinea/ or guinea-bissau/ or guyana/ or haiti/ or honduras/ or independent state of samoa/ or exp india/ or indian ocean islands/ or indochina/ or indonesia/ or iran/ or iraq/ or jamaica/ or jordan/ or kazakhstan/ or kenya/ or kosovo/ or kuwait/ or kyrgyzstan/ or laos/ or lebanon/ or liechtenstein/ or lesotho/ or liberia/ or libya/ or madagascar/ or malaysia/ or malawi/ or mali/ or malta/ or mauritania/ or mauritius/ or mekong valley/ or melanesia/ or micronesia/ or monaco/ or mongolia/ or montenegro/ or morocco/ or mozambique/ or myanmar/ or namibia/ or nepal/ or nicaragua/ or niger/ or nigeria/ or oman/ or pakistan/ or palau/ or exp panama/ or papua new guinea/ or paraguay/ or peru/ or philippines/ or qatar/ or "republic of belarus"/ or "republic of north macedonia"/ or romania/ or exp russia/ or rwanda/ or "saint kitts and nevis"/ or saint lucia/ or "saint vincent and the grenadines"/ or "sao tome and principe"/ or saudi arabia/ or serbia/ or sierra leone/ or senegal/ or seychelles/ or singapore/ or somalia/ or south africa/ or south sudan/ or sri lanka/ or sudan/ or suriname/ or syria/ or taiwan/ or tajikistan/ or tanzania/ or thailand/ or timor-leste/ or togo/ or tonga/ or "trinidad and tobago"/ or tunisia/ or turkmenistan/ or uganda/ or ukraine/ or united arab emirates/ or uruguay/ or uzbekistan/ or vanuatu/ or venezuela/ or vietnam/ or west indies/ or yemen/ or zambia/ or zimbabwe/ (1240847)

85 "Organisation for Economic Co-Operation and Development"/ (464)

86 australasia/ or exp australia/ or austria/ or baltic states/ or belgium/ or exp canada/ or chile/ or colombia/ or costa rica/ or czech republic/ or exp denmark/ or estonia/ or europe/ or finland/ or exp france/ or exp germany/ or greece/ or hungary/ or iceland/ or ireland/ or israel/ or exp italy/ or exp japan/ or korea/ or latvia/ or lithuania/ or luxembourg/ or mexico/ or netherlands/ or new zealand/ or north america/ or exp norway/ or poland/ or portugal/ or exp "republic of korea"/ or "scandinavian and nordic countries"/ or slovakia/ or slovenia/ or spain/ or sweden/ or switzerland/ or turkey/ or exp united kingdom/ or exp united states/ (3432805)

87 European Union/ (17334)

88 Developed Countries/ (21208)

89 or/85-88 (3448340)

90 84 not 89 [OECD search filter NICE 2021] (1152820)

91 83 not 90 [UB among Acute Care Staff with non-OECD countries removed] (977)

92 (exp Child/ or Adolescent/ or exp Infant/) not exp Adult/ (2073393)

93 91 not 92 [Child studies removed] (902)

94 (elder mistreat* or elder abuse* or elder neglect*).tw,kf. (1932)

95 93 not 94 [Elder abuse studies removed] (878)

96 exp united states/ (1440539)

97 (USA or "united states" or america*).ti,ab,in,kf. (5886737)

98 96 or 97 (6652358)

99 95 and 98 (316)

Google Scholar (Via Harzing’s Publish or Perish)

Date searched: 25-08-2022

Records downloaded: 14

Search 1. 14 records found

bullying | harassment | discrimination|unprofessional AND workplace|worker|staff AND model|framework|concept|idea|opinion|theory|view|perception|attitude|theories [Searched in Publish or Perish Title Field]

USA | america | united states [KEywords field]

Years limited to 2022-2022

Search 2. 0 records found

interventions|strategies|techniques|program|programs|programme|programmes AND unprofessional|bullying|harassment|discrimination AND nurse|doctor|paramedic|hospital|ambulance|staff|professional AND emergency|acute|trauma [searched in Publish or Perish Keywords field]

USA | america | united states [KEywords field]

Years limited to 2022-2022

Patient Safety Network <https://psnet.ahrq.gov/>

Date searched: 25-08-2022

Records found: 1

abuse bullying unprofessional harassment discrimination microaggression incivility intimidation humiliation hostility mobbing hazing gaslighting malicious

Limited to Ambulatory Care setting OR Limit to Hospital setting. Added in last 6 months

## References

1. Dada S, Dalkin S, Mukumbang FC, Gilmore B, Hunter R. Applying and reporting relevance, richness and rigour in realist evidence appraisals: Advancing key concepts in realist reviews. Res Synth Methods. 2023;1–11.

2. Haddaway NR, Grainger MJ, Gray CT. citationchaser: An R package and Shiny app for forward and backward citations chasing in academic searching. Zenodo, Feb. 2021;16.

3. Gillespie GL, Grubb PL, Brown K, Boesch MC, Ulrich DL. “Nurses eat their young”: A novel bullying educational program for student nurses. J Nurs Educ Pract. 2017;7(7):11.

4. General Medical Council. Building a supportive environment: a review to tackle undermining and bullying in medical education and training. General Medical Council. 2015.

5. Pisklakov S, Tilak V, Patel A, Xiong M. Bullying and Aggressive Behavior among Health Care Providers: Literature Review. Adv Anthropol. 2013;03(04):179–82.

6. British Medical Association. Bullying and harassment : how to address it and create a supportive and inclusive culture. 2018.

7. Felblinger DM. Bullying, incivility, and disruptive behaviors in the healthcare setting: identification, impact, and intervention. Front Health Serv Manage. 2009;25(4):13–23.

8. Jones A, Kelly D. Deafening silence? Time to reconsider whether organisations are silent or deaf when things go wrong. BMJ Qual Saf. 2014;23(9):709–13.

9. Westbrook J, Sunderland N, Atkinson V, Jones C, Braithwaite J. Endemic unprofessional behaviour in health care: the mandate for a change in approach. Med J Aust [Internet]. 2018;209(9):380–1. Available from: https://doi.org/10.5694/mja17.01261

10. Cooper K. Ending the silence. BMA [Internet]. 2018; Available from: https://www.bma.org.uk/news-and-opinion/ending-the-silence

11. Illing J, Carter M, Thompson NJ, Crampton PES, Morrow GM, Howse JH, et al. Evidence synthesis on the occurrence, causes, management of bullying and harassing behaviours to inform decision making in the NHS. 2013;44(February):54–168.

12. Mannion R, Davies H, Powell M, Blenkinsopp J, Millar R, McHale J, et al. Healthcare scandals and the failings of doctors: Do official inquiries hold the profession to account? J Health Organ Manag. 2019;33(2):221–40.

13. Walton MM. Hierarchies: The Berlin wall of patient safety. Qual Saf Heal Care. 2006;15(4):229–30.

14. Barzallo Salazar MJ, Minkoff H, Bayya J, Gillett B, Onoriode H, Weedon J, et al. Influence of surgeon behavior on trainee willingness to speak up: A randomized controlled trial. J Am Coll Surg [Internet]. 2014;219(5):1001–7. Available from: http://dx.doi.org/10.1016/j.jamcollsurg.2014.07.933

15. Quinlan E, Robertson S, Miller N, Robertson-Boersma D. Interventions to reduce bullying in health care organizations: A scoping review. Heal Serv Manag Res. 2014;27(1):33–44.

16. Rogers-Clark C, Pearce S, Cameron M. Management of disruptive behaviour within nursing work environments: a comprehensive systematic review of the evidence. JBI Libr Syst Rev. 2009;7(15):615–78.

17. Armstrong N. Management of Nursing Workplace Incivility in the Health Care Settings: A Systematic Review. Work Heal Saf. 2018;66(8):403–10.

18. Benjamin A. Names, hair, identity and micro aggressions. 2021;(1):1–5. Available from: https://www.bma.org.uk/news-and-opinion/names-hair-identity-and-micro-aggressions

19. Parizad N, Hassankhani H, Rahmani A, Mohammadi E, Lopez V, Cleary M. Nurses’ experiences of unprofessional behaviors in the emergency department: A qualitative study. Nurs Heal Sci. 2018;20(1):54–9.

20. Blackstock S, Salami B, Cummings GG. Organisational antecedents, policy and horizontal violence among nurses: An integrative review. J Nurs Manag. 2018;26(8):972–91.

21. Cruz D, Rodriguez Y, Mastropaolo C. Perceived microaggressions in health care: A measurement study. PLoS One. 2019;14(2):1–11.

22. Keller S, Yule S, Zagarese V, Parker SH. Predictors and triggers of incivility within healthcare teams: A systematic review of the literature. BMJ Open. 2020;10(6):1–15.

23. Kline R. Middlesex University London. 2021. Racism which impacts healthcare staff endangers patient care. Available from: https://mdxminds.com/2021/11/22/racism-which-impacts-healthcare-staff-endangers-patient-care/

24. Riskin A, Erez A, Foulk TA, Kugelman A, Gover A, Shoris I, et al. The impact of rudeness on medical team performance: A randomized trial. Pediatrics. 2015;136(3):487–95.

25. Kaiser JA. The relationship between leadership style and nurse-to-nurse incivility: turning the lens inward. J Nurs Manag. 2017;25(2):110–8.

26. Wild JRL, Ferguson HJM, McDermott FD, Hornby ST, Gokani VJ. Undermining and bullying in surgical training: A review and recommendations by the Association of Surgeons in Training. Int J Surg. 2015;23:S5–9.

27. Salin D. Ways of explaining workplace bullying: A review of enabling, motivating and precipitating structures and processes in the work environment. Hum Relations. 2003;56(10):1213–32.

28. Ross S, Jabbal J, Chauhan K, Maguire D, Randhawa M, Dahir S. Workforce race inequalities and inclusion in NHS providers. 2020;(July).

29. Ariza-Montes A, Muniz NM, Montero-Simó MJ, Araque-Padilla RA. Workplace bullying among healthcare workers. Int J Environ Res Public Health. 2013;10(8):3121–39.

30. British Medical Association. Workplace bullying and harassment of doctors A review of recent research [Internet]. British Medical Association. 2017. Available from: file:///Users/VWC/Downloads/Bullying and harassment research review v7 WEB.pdf

31. Naylor MJ, Boyes C, Killingback C. “You’ve broken the patient”: Physiotherapists’ lived experience of incivility within the healthcare team - An Interpretative Phenomenological Analysis. Physiotherapy [Internet]. 2022;117:89–96. Available from: https://doi.org/10.1016/j.physio.2022.09.001

32. Hickson GB, Pichert JW, Webb LE, Gabbe SG. A complementary approach to promoting professionalism: Identifying, measuring, and addressing unprofessional behaviors. Acad Med. 2007;82(11):1040–8.

33. Hawkins N, Jeong SYS, Smith T, Sim J. A conflicted tribe under pressure: A qualitative study of negative workplace behaviour in nursing. J Adv Nurs. 2022;17(September):17.

34. Hutchinson M, Jackson D, Wilkes L, Vickers MH. A new model of bullying in the nursing workplace organizational characteristics as critical antecedents. Adv Nurs Sci. 2008;31(2):60–71.

35. Kline R. A review into culture and bullying at University Hospitals of North Midlands NHS Trust. 2022;(March).

36. Rocker CF. Addressing nurse-to-nurse bullying to promote nurse retention. Online J Issues Nurs. 2008;13(3):1–10.

37. Wilson JL. An exploration of bullying behaviours in nursing: a review of the literature. Br J Nurs. 2016;25(6):303–6.

38. Almost J, Doran DM, Mcgillis Hall L, Spence Laschinger HK. Antecedents and consequences of intra-group conflict among nurses. J Nurs Manag. 2010;18(8):981–92.

39. Hemmings N, Buckingham H, Oung C, Palmer W. Attracting, supporting and retaining a diverse NHS workforce [Internet]. 2021. Available from: www.nuffieldtrust.org.uk/research

40. Owens J, Singh G, Cribb A. Austerity and Professionalism: Being a Good Healthcare Professional in Bad Conditions. Heal Care Anal [Internet]. 2019;27(3):157–70. Available from: https://search.ebscohost.com/login.aspx?direct=true&db=cin20&AN=137793764&site=ehost-live

41. Hughes A. Being bullied what an insight. Br J Perioper Nurs. 2003;13(4).

42. Royal College of Nursing. Bullying and harassment: good practice guidance for preventing and addressing bullying and harassment in health and social care organisations. Vol. 66. 2014.

43. Longo J, Hain D. Bullying: a hidden threat to patient safety. Nephrol Nurs J. 2014;41(2):193–9; quiz 200.

44. Manton AP. Bullying: A Pebble in the Pond. J Emerg Nurs [Internet]. 2017;43(5):389–90. Available from: https://ovidsp.ovid.com/ovidweb.cgi?T=JS&CSC=Y&NEWS=N&PAGE=fulltext&D=med14&AN=28822462

45. Osatuke K, Moore SC, Ward C, Dyrenforth SR, Belton L. Civility, Respect, Engagement in the Workforce (CREW). J Appl Behav Sci. 2009;45(3):384–410.

46. O’Connell KM, Garbark RL, Nader KC. Cognitive Rehearsal Training to Prevent Lateral Violence in a Military Medical Facility. J Perianesthesia Nurs. 2019;34(3):645-653.e1.

47. Nicotera AM, Mahon MM, Wright KB. Communication that builds teams: Assessing a nursing conflict intervention. Nurs Adm Q. 2014;38(3):248–60.

48. Parker KM, Harrington A, Smith CM, Sellers KF, Millenbach L. Creating a Nurse-Led Culture to Minimize Horizontal Violence in the Acute Care Setting: A Multi-Interventional Approach. J Nurses Prof Dev. 2016;32(2):56–63.

49. Hawkins N, Jeong SYS, Smith T, Sim J. Creating respectful workplaces for nurses in regional acute care settings: A quasi-experimental design. Nurs Open. 2022;(April 2022):78–89.

50. Academy of Medical Royal Colleges. Creating supportive environments: Tackling behaviours that undermine a culture of safety [Internet]. AoMRC Trainee Doctors’ Group. Academy of Medical Royal Colleges; 2016. Available from: http://www.aomrc.org.uk/wp-content/uploads/2016/09/Creating_supportive_environments_280916-2.pdf

51. Alspach G. Critical care nurses as coworkers: are our interactions nice or nasty? Crit Care Nurse. 2007;27(3):10–4.

52. Warrner J, Sommers K, Zappa M, Thornlow DK. Decreasing work place incivility. Nurs Manage. 2016;47(1):22–30.

53. Speck RM, Foster JJ, Mulhern VA, Burke S V., Sullivan PG, Fleisher LA. Development of a professionalism committee approach to address unprofessional medical staff behavior at an academic medical center. Jt Comm J Qual Patient Saf. 2014;40(4):161–7.

54. Villafranca A, Hamlin C, Enns S, Jacobsohn E. Disruptive behaviour in the perioperative setting: a contemporary review. Can J Anesth. 2017;64(2):128–40.

55. Babenko-Mould Y, Laschinger HKS. Effects of incivility in clinical practice settings on nursing student burnout. Int J Nurs Educ Scholarsh [Internet]. 2014;11(1):145–54. Available from: https://ovidsp.ovid.com/ovidweb.cgi?T=JS&CSC=Y&NEWS=N&PAGE=fulltext&D=med11&AN=25367690

56. Dimarino TJ. Eliminating Lateral Violence in the Ambulatory Setting: One Center’s Strategies. AORN J [Internet]. 2011;93(5):583–8. Available from: https://ovidsp.ovid.com/ovidweb.cgi?T=JS&CSC=Y&NEWS=N&PAGE=fulltext&D=med8&AN=21530706

57. Sillero AS, Buil N. Enhancing interprofessional collaboration in perioperative setting from the qualitative perspectives of physicians and nurses. Int J Environ Res Public Health [Internet]. 2021;18(20). Available from: https://www.mdpi.com/1660-4601/18/20/10775/pdf

58. Stagg SJ, Sheridan D, Jones RA, Speroni KG. Evaluation of a Workplace Bullying Cognitive Rehearsal Program in a Hospital Setting. J Contin Educ Nurs. 2011;42(9):395–403.

59. Edwards SL, O’Connell CF. Exploring bullying: Implications for nurse educators. Nurse Educ Pract [Internet]. 2007;7(1):26–35. Available from: https://www.sciencedirect.com/science/article/pii/S1471595306000485

60. McKenzie LN, Shaw L, Jordan JE, Alexander M, O’Brien M, Singer SJ, et al. Factors Influencing the Implementation of a Hospitalwide Intervention to Promote Professionalism and Build a Safety Culture: A Qualitative Study. Jt Comm J Qual Patient Saf. 2019;45(10):694–705.

61. Banerjee D, Nassikas NJ, Singh P, Andrea SB, Zhang AY, Aswad Y, et al. Feasibility of an Antiracism Curriculum in an Academic Pulmonary, Critical Care, and Sleep Medicine Division. Ats Sch. 2022;3(3):433–48.

62. Purpora C, Blegen MA. Horizontal Violence and the Quality and Safety of Patient Care: A Conceptual Model. Nurs Res Pract. 2012;2012(May 2012):1–5.

63. Zhang X, Xiong L. Impact of Nurse Horizontal Violence and Coping Strategies: A Review. Yangtze Med. 2019;03(04):289–300.

64. Baldwin CA, Hanrahan K, Edmonds SW, Krumm AM, Sy A, Jones A, et al. Implementation of Peer Messengers to Deliver Feedback: An Observational Study to Promote Professionalism in Nursing. Jt Comm J Qual Patient Saf. 2022;000(i):1–12.

65. Dixon-Woods M, Campbell A, Martin G, Willars J, Tarrant C, Aveling EL, et al. Improving Employee Voice about Transgressive or Disruptive Behavior: A Case Study. Acad Med. 2019;94(4):579–85.

66. Credland NJ, Whitfield C. Incidence and impact of incivility in paramedicine: A qualitative study. Emerg Med J [Internet]. 2022;39(1):52–6. Available from: https://ovidsp.ovid.com/ovidweb.cgi?T=JS&CSC=Y&NEWS=N&PAGE=fulltext&D=medl&AN=34039640

67. Nikstaitis T, Simko LC. Incivility among intensive care nurses: The effects of an educational intervention. Dimens Crit Care Nurs [Internet]. 2014;33(5):293–301. Available from: https://ovidsp.ovid.com/ovidweb.cgi?T=JS&CSC=Y&NEWS=N&PAGE=fulltext&D=med11&AN=25140748

68. Johnson SL, Haerling KA, Yuwen W, Huynh V, Le C. Incivility and Clinical Performance, Teamwork, and Emotions: A Randomized Controlled Trial. J Nurs Care Qual. 2020;35(1):70–6.

69. Lovejoy-Bluem A. Incivility and/or Human Kind(ness) in the NICU. Acad Neonatal Nurs. 2016;35(3):173–4.

70. Hutchinson M, Wilkes L, Jackson D, Vickers MH. Integrating individual, work group and organizational factors: Testing a multidimensional model of bullying in the nursing workplace. J Nurs Manag [Internet]. 2010;18(2):173–81. Available from: https://www.ncbi.nlm.nih.gov/pubmed/20465745

71. Gamble Blakey A, Smith-Han K, Anderson L, Collins E, Berryman E, Wilkinson TJ. Interventions addressing student bullying in the clinical workplace: A narrative review. BMC Med Educ. 2019;19(1):1–13.

72. Tuffour I. It is like ‘judging a book by its cover’: An exploration of the lived experiences of Black African mental health nurses in England. Nurs Inq. 2022;29(1).

73. Barrett A, Piatek C, Korber S, Padula C. Lessons learned from a lateral violence and team-building intervention. Nurs Adm Q. 2009;33(4):342–51.

74. Royal College of Surgeons of England. MANAGING DISRUPTIVE BEHAVIOURS IN SURGERY: A Guide to Good Practice [Internet]. 2021. Available from: www.rcseng.ac.uk/standardsandguidance

75. Efe SY, Ayaz S. Mobbing against nurses in the workplace in Turkey. Int Nurs Rev [Internet]. 2010;57(3):328–34. Available from: https://ovidsp.ovid.com/ovidweb.cgi?T=JS&CSC=Y&NEWS=N&PAGE=fulltext&D=med8&AN=20796062

76. Hawkins N, Jeong S, Smith T. New graduate registered nurses’ exposure to negative workplace behaviour in the acute care setting: An integrative review. Int J Nurs Stud [Internet]. 2019;93:41–54. Available from: https://doi.org/10.1016/j.ijnurstu.2018.09.020

77. Churchman JJ, Doherty C. Nurses’ views on challenging doctors’ practice in an acute hospital. Nurs Stand. 2010;24(40):42–7.

78. Stevens S. Nursing workforce retention: Challenging a bullying culture. Health Aff. 2002;21(5):189–93.

79. Bry A, Wigert H. Organizational climate and interpersonal interactions among registered nurses in a neonatal intensive care unit: A qualitative study. J Nurs Manag (John Wiley Sons, Inc) [Internet]. 2022;30(6):2031–8. Available from: https://search.ebscohost.com/login.aspx?direct=true&db=cin20&AN=159455054&site=ehost-live

80. Markwell A, Smith S, Michalski M, Conroy S, Bell A. Performance management versus bullying and harassment: An educator perspective. EMA - Emerg Med Australas [Internet]. 2015;27(5):468–72. Available from: https://search.ebscohost.com/login.aspx?direct=true&db=cin20&AN=109542016&site=ehost-live

81. Babla K, Lau S, Akindolie O, Radia T, Modi N, Kingdon C, et al. Racial microaggressions within respiratory and critical care medicine. Lancet Respir Med [Internet]. 2021;9(3):e27–8. Available from: http://dx.doi.org/10.1016/S2213-2600(21)00001-1

82. Embree JL, Bruner DA, White A. Raising the Level of Awareness of Nurse-to-Nurse Lateral Violence in a Critical Access Hospital. Nurs Res Pract. 2013;2013:1–7.

83. O’Keeffe DA, Brennan SR, Doherty EM. Resident Training for Successful Professional Interactions. J Surg Educ [Internet]. 2022;79(1):107–11. Available from: https://doi.org/10.1016/j.jsurg.2021.08.017

84. Stone L, Phillips C, Douglas KA. Sexual assault and harassment of doctors, by doctors: a qualitative study. Med Educ [Internet]. 2019;53(8):833–43. Available from: https://onlinelibrary.wiley.com/doi/abs/10.1111/medu.13912

85. Ceravolo DJ, Schwartz DG, Foltz-Ramos KM, Castner J. Strengthening communication to overcome lateral violence. J Nurs Manag. 2012;20(5):599–606.

86. Thorsness R, Sayers B. Systems Approach to Resolving Conduct Issues Among Staff Members. AORN J [Internet]. 1995;61(1):197–202. Available from: https://search.ebscohost.com/login.aspx?direct=true&db=cin20&AN=107404234&site=ehost-live

87. Phillips JM, Stalter AM, Winegardner S, Wiggs C, Jauch A. Systems thinking and incivility in nursing practice: An integrative review. Nurs Forum [Internet]. 2018;53(3):286–98. Available from: https://www.ncbi.nlm.nih.gov/pubmed/29359482

88. NHS Employers. Tackling bullying in ambulance trusts: a guide for action. 2016;

89. Griffin M. Teaching cognitive rehearsal as a shield for lateral violence: an intervention for newly licensed nurses. J Contin Educ Nurs. 2004;35(6):257–63.

90. Asi Karakaş S, Okanli AE. The Effect of Assertiveness Training on the Mobbing That Nurses Experience. Work Heal Saf. 2015;63(10):446–51.

91. Weaver KB. The effects of horizontal violence and bullying on new nurse retention. J Nurses Prof Dev. 2013;29(3):138–42.

92. Işık I, Gümüşkaya O, Şen S, Arslan Özkan H. The Elephant in the Room: Nurses’ Views of Communication Failure and Recommendations for Improvement in Perioperative Care. AORN J. 2020;111(1):e1–15.

93. Leiter MP, Laschinger HKS, Day A, Oore DG. The impact of civility interventions on employee social behavior, distress, and attitudes. J Appl Psychol. 2011;96(6):1258–74.

94. Tame S. The relationship between continuing professional education and horizontal violence in perioperative practice. J Perioper Pract. 2012;22(7):220–5.

95. Demarco RF, Roberts SJ, Chandler GE. The Use of a Writing Group to Enhance Voice and Connection Among Staff Nurses. 2005;21(3):85–90.

96. Blakey AG, Anderson L, Smith-Han K, Wilkinson T, Collins E, Berryman E. Time to stop making things worse: An imperative focus for healthcare student bullying research. N Z Med J. 2018;131(1479):81–5.

97. Bamberger E, Bamberger P. Unacceptable behaviours between healthcare workers: just the tip of the patient safety iceberg. BMJ Qual Saf [Internet]. 2022;31(9):638–41. Available from: https://www.ncbi.nlm.nih.gov/pubmed/35428683

98. Allen B. Understanding bullying in healthcare organisations. 2015;30(14):259.

99. Pavithra A, Sunderland N, Callen J, Westbrook J. Unprofessional behaviours experienced by hospital staff : qualitative analysis of narrative comments in a longitudinal survey across seven hospitals in Australia. BMC Health Serv Res [Internet]. 2022;1–15. Available from: https://doi.org/10.1186/s12913-022-07763-3

100. Anonymous. When bullying affects patient safety. AORN J. 2018;108(1):78–80.

101. Al-Rais A. Why we should avoid handover hostility. BMJ [Internet]. 2017;356:j1272. Available from: https://ovidsp.ovid.com/ovidweb.cgi?T=JS&CSC=Y&NEWS=N&PAGE=fulltext&D=med14&AN=28320693

102. Beale D, Leather P. Working with care – improving working relationships in health and social care : self-assessment tools for health [Internet]. Royal College of Nursing. Royal College of Nursing - RCN; 2005. Available from: https://www.rcn.org.uk/-/media/royal-college-of-nursing/documents/publications/2015/september/004972.pdf?la=en

103. Anderson K. Workplace aggression and violence: nurses and midwives say NO. Aust Nurs J. 2011;19(1):26–9.

104. Chadwick S, Travaglia J. Workplace bullying in the Australian health context: a systematic review. J Heal Organ Manag. 2017;31(3):286–301.

105. Carter M, Thompson N, Crampton P, Morrow G, Burford B, Gray C, et al. Workplace bullying in the UK NHS: A questionnaire and interview study on prevalence, impact and barriers to reporting. BMJ Open. 2013;3(6).

106. Stagg SJ, Sheridan DJ, Jones RA, Speroni KG. Workplace Bullying: The Effectiveness of a Workplace Program. Aust Nurs midwifery J. 2017;24(9):34–6.

107. Lewis D. Workplace Culture at Southwestern Ambulance NHS Foundation Trust. 2018.

108. Adams L, Bryan V. Workplace harassment: The leadership factor. Healthc Manag Forum [Internet]. 2021;34:81–6. Available from: https://journals.sagepub.com/home/HMF

109. Blackstock S, Cummings G, Glanfield F, Yonge O. A review: Developing an ecological model approach to co‐worker incivility experiences of new graduate nurses. J Adv Nurs. 2022;(April 2021):1–16.

110. Miller DT, Chen EH. Helping the learner to deal with microaggressions in the workplace: Individual, programmatic, and institutional-level responses. AEM Educ Train [Internet]. 2021;5(S1):S140–3. Available from: https://ovidsp.ovid.com/ovidweb.cgi?T=JS&CSC=Y&NEWS=N&PAGE=fulltext&D=pmnm5&AN=34616989

111. Chipps EM, McRury M. The development of an educational intervention to address workplace bullying: A pilot study. J Nurses Staff Dev. 2012;28(3):94–8.

112. Lasater K, Mood L, Buchwach D, Dieckmann NF. Reducing incivility in the workplace: Results of a three-part educational intervention. J Contin Educ Nurs. 2015;46(1):15–24.
