# Supplementary material for: Drivers of unprofessional behaviour between staff in acute care hospitals: a realist review
Source: BMC Health Serv Res. 2023 Nov 30;23:1326. doi: 10.1186/s12913-023-10291-3 (PMC10687856; doi:10.1186/s12913-023-10291-3)
Supplement: Supplementary file 4 — Additional file 4. Characteristics of included sources. [file 12913_2023_10291_MOESM4_ESM.docx]

# Additional File 4. Characteristics of included sources.

| **Document** | **Source type / study design** | **Country of conduct or focus** | **Sample** | **Healthcare type** | **Area of unprofessional behaviour** | **Key findings & relevancy** |
| --- | --- | --- | --- | --- | --- | --- |
| Step 1 – Initial theories | | | | | | |
| Ariza-Montes et al. (2013)(1) | Survey | EU member states | Sub-sample of 284 health professionals | General healthcare | Bullying | Draws on data from the 5^th^ European Working Conditions Survey to identify predictors of bullying in the healthcare workplace. Predictors included shift working, monotonous tasks, stress, and few promotion opportunities. |
| Armstrong (2018)(2) | Systematic review | n/a | Nurses, 10 studies | General healthcare | Incivility | A systematic review of strategies used to address incivility in the nursing workplace. It found that some strategies such as communication training and education may have some promise for managing incivility. But studies were low quality. |
| Barzallo Salazar et al. (2014)(3) | Simulation study / RCT design | USA | 55 trainees, encouraged (n=28) and discouraged (n=27) groups | Surgery | ‘Discouraging environment’ | This study is RCT that explores how surgeon behaviour affects trainee willingness to speak up during surgeries. Positive surgeon behaviour leads to increased trainee speaking up and improved patient safety. |
| Benjamin (2021)(4) | Opinion article | UK | n/a | General healthcare | Microaggressions | Opinion article summarising what microaggressions are and how they are experienced in the healthcare workplace. Offers also some accounts from those who have experienced them. |
| Blackstock, Salami and Cummings (2018)(5) | Integrative review | n/a | Nurses, 22 studies | General healthcare | Horizontal violence | An integrative review exploring the organisational antecedents of horizontal violence among nurses. Working conditions, organisational culture, and leadership roles were all found to be key themes. |
| British Medical Association (2017)(6) | Narrative review | UK | Doctors, unclear number of studies | General healthcare | Bullying and harassment | A review of research by the British Medical Association assessing prevalence of bullying and harassment affecting doctors, as well as antecedents and solutions. |
| British Medical Association (2018)(7) | Report | UK | n/a | General healthcare | Bullying and harassment | Report by the British Medical Association about how to address bullying and harassment in the healthcare workplace including suggestions such as intervening early. |
| Cooper (2018)(8) | Opinion piece | UK | n/a | General healthcare | Bullying | Highlights experience of bullying by different people in the NHS with quotes. Highlights some strategies people can take to address it, such as documenting incidences. |
| Cruz, Rodriguez and Mastropaolo (2019)(9) | Cross-sectional study | USA | 296 African American and Latino participants | General healthcare | Microaggressions | Study seeking to provide psychometric evidence for the Microaggressions in Health Care Scale. Provides significant background information regarding microaggressions. Microaggressions were found to correlate with mental health symptoms. |
| Felblinger (2009)(10) | Editorial paper | n/a | n/a | General healthcare | Bullying, incivility, and disruptive behaviours | An editorial that assesses how to identify bullying, incivility and disruptive behaviours, as well as how to mitigate their impact. Factors such as changing hierarchies, conflicting loyalties, and stress were stated to increase risk of such behaviour. |
| General Medical Council (2015)(11) | Report drawing on interviews | n/a | 12 sites | Surgery and obstetrics and gynaecology | Undermining and bullying | A report and review of bullying and undermining behaviours in medical education and training. Explores the groups that experience such behaviours more, factors contributing to them, and wider context for such behaviours. |
| Gillespie et al. (2017)(12) | Intervention | USA | Five academic campuses, nursing students | General healthcare | Bullying | Article describing the development process and utility of an educational intervention for use by nursing faculty with nursing students performed in a university context. |
| Illing et al. (2013)(13) | Evidence synthesis | n/a | 160 papers, 55 described in detail | General healthcare | Bullying and harassment | An evidence synthesis exploring occurrence, causes, and management of bullying and harassment behaviours in healthcare. |
| Jones and Kelly (2014)(14) | Editorial paper | UK | n/a | General healthcare | Organisational deafness | An editorial exploring organisational deafness and highlights how people often do speak up but that their concerns fall on deaf ears. Suggests some means to attempt to resolve this. |
| Kaiser (2017)(15) | Survey | USA | 237 staff nurses | General healthcare | Incivility | Exploration of leadership style and the relationship to nurse-to-nurse incivility. Transformational leadership was found to correlate with lower levels of incivility. However, leadership style was not a very strong factor affecting incivility incidence. |
| Keller et al. (2020)(16) | Systematic review | n/a | 53 included papers | General healthcare | Incivility | Systematic review identifying predictors of incivility within healthcare teams. It found conceptualisation of incivility was subjective and varied, and quality of studies low. Results were inconsistent regarding individual characteristics but situational and cultural predictors were identified. |
| Kline (2021)(17) | Opinion piece / online blog | UK | n/a | General healthcare | Racism | Explores the risks associated with not tackling racism in the NHS. These include depriving patients of the best talent, blame culture affecting patient safety, impact to staff health, loss of patient benefit attained by greater representation. |
| Mannion et al. (2019)(18) | Literature review and textual analysis | UK | Unclear number of documents, 7 in textual analysis | General healthcare | Misconduct | Examines how doctor misconduct can be understood using metaphors of bad apples, barrels, or orchards. |
| Parizad et al. (2018)(19) | Qualitative study | Iran | 15 registered nurses | Emergency department | Unprofessional behaviours | Explores Iranian nurse’s experiences of unprofessionalism in the emergency department. Explores contributors to these behaviours as well as prevalence. |
| Pislakov et al. (2013)(20) | Literature review | USA | Unclear number of sources | Healthcare in general | Bullying and aggressive behaviour | An informal literature review that explores what types of people engage in bullying, how it is defined, and its causes. |
| Quinlan et al. (2014)(21) | Scoping review | n/a | 8 sources | Healthcare in general | Bullying | A scoping review exploring interventions to reduce bullying in healthcare organisations. It revealed eight articles that included education, championing, and zero-tolerance policies. |
| Riskin et al. (2015)(22) | Simulation study RCT | USA | 24 neonatal intensive care unit teams | Neonatal intensive care unit | Rudeness | A simulation RCT with exposure to rudeness or control conditions. It found that rudeness had adverse consequences on information sharing and help-seeking behaviours with implications for patient safety. |
| Riskin et al. (2017)(23) | Simulation study RCT | USA | 39 neonatal intensive care unit teams | Neonatal intensive care unit | Rudeness | A simulation RCT with exposure to rudeness or control, as well as rudeness with and without a preventative or therapeutic intervention. It was found that cognitive bias modification was able to mitigate adverse effects of rudeness. |
| Rogers-Clark, Pearce and Cameron (2009)(24) | Systematic review | n/a | 24 papers | General healthcare | Disruptive behaviour | A systematic review of interventions to manage disruptive clinician behaviour in the nursing work environment. It found few sources evaluating effectiveness of interventions. |
| Ross et al. (2020)(25) | Report drawing on interviews | UK | 12 NHS staff and three case studies of NHS Providers | Acute care | Racism | A report investigating workforce race inequalities and inclusion in NHS providers. Assesses lived experience of a number of NHS staff as well as interventions used to address these race inequalities in NHS provider case studies. |
| Salin (2003)(26) | Review | n/a | Non-healthcare, bullying in general | Non-healthcare | Bullying | A review assessing ways of understanding workplace bullying in general and the factors that precipitate it. Such factors include antecedents including power imbalances, low perceived costs, and motivating structures such as reward systems. |
| Walton (2006)(27) | Editorial | n/a | n/a | Healthcare in general | Hierarchy and power | Explores the impact of hierarchy on the ability to speak up from a patient safety perspective. |
| Westbrook et al. (2018a)(28) | Editorial | Australia | n/a | Healthcare in general | Unprofessional behaviour | Highlights the impact of unprofessional behaviour in the Australian health care system, as well as the limited intervention evidence base while calling for greater culture change efforts. |
| Wild et al. (2015)(29) | Editorial | UK | n/a | Surgery | Undermining and bullying | Recommendations by the Association of Surgeons in Training to help create a positive learning environment free of undermining and bullying. |
| Step 2 – Exhaustive search | | | | | | |
| Academy of Medical Royal Colleges (2016)(30) | Report | UK | n/a | Trainees (general) | Undermining and bullying | Explores causes of UB based on findings of a one-day seminar and proposes strategies to address it. |
| Adams & Bryan (2021) (31) | Editorial | Canada | n/a | General healthcare | Harassment | Highlights the role of leadership in creating an environment permissive of UB in healthcare in a Canadian setting. |
| Al-Rias (2017)(32) | Editorial | UK | n/a | Emergency department | ‘Handover hostility’ | Describes a scenario of handover hostility in the English NHS and explores reasons for it. |
| Allen (2015)(33) | Narrative review | UK | n/a | General healthcare | Bullying | Narrative review of bullying with a focus on the English NHS. Presents causes of bullying and preventative measures one can take. |
| Almost et al. (2010)(34) | Testing theoretical model | Canada | 277 acute care nurses | Acute care | Conflict | Quantitatively testing a theoretical model of causes of intra-group conflict among nurses with a non-experimental design. Explores how factors like self-evaluation, complexity of nursing care, and interactional justice affect incidence of conflict. |
| Alspach (2007)(35) | Editorial | No specific country | n/a | General healthcare | Lateral hostility | Explores causes, impacts, and prevention of lateral hostility between critical care nurses. |
| Anderson (2011)(36) | Editorial | Australia | n/a | Emergency department | Workplace aggression & horizontal violence | Explores violence from patients but also horizontal violence, its causes, the harm it causes, and how legislation may reduce it. |
| Anonymous (2018)(37) | Case study | No specific country | n/a | Acute care | Bullying | Exploration of how a single event of bullying in the operating room led to an impact on patient safety. |
| Asi Karakaş and Okanli (2015)(39) | Intervention | Turkey | 30 nurses | Acute care | Mobbing | This study reported an evaluation of an assertiveness training intervention in 30 nurses who experienced a high level of mobbing. Results indicated a statistically significant fall in mobbing after the intervention and an increase in assertiveness. |
| Babenko-Mould and Laschinger (2014)(40) | Survey | Canada | 126 year 4 nursing students | Acute care | Incivility | Focuses on effect on incivility on nurse burnout and talks about strategies to mitigate this impact. It finds that incivility is strongly related to burnout. |
| Babla et al. (2021)(41) | Letter | UK | n/a | Critical care | Microaggressions | Focuses on racial microaggressions and what they are, how they manifest in the healthcare workplace, and how they should be addressed. |
| Baldwin et al (2022)(129) | Intervention | USA | Three academic medical centres | Acute care | Professionalism | Descriptive study analysing the types of reports received during the intervention to promote professionalism with nurses. |
| Bamberger and Bamberger (2022)^224^ | Editorial | n/a | n/a | General healthcare | Unacceptable behaviours | Editorial exploring the impact of unacceptable behaviours between healthcare workers on patient safety. |
| Banerjee et al. (2022)(131) | Intervention | USA | Division faculty members (n = 41) and pulmonary and critical care fellows (n = 12) | Acute care | Racism | Assessed the feasibility of a year-long antiracism educational study. As it was mostly a feasibility study, postintervention surveying indicated a 15% increase in self-directed learning on related topics. |
| Barrett et al. (2009)(42) | Intervention | USA | 59 pre-intervention and 45 post-intervention nurses | Critical care | Lateral violence | The study assessed a team-building intervention to reduce lateral violence, using mixed methods. The intervention was found to improve group cohesion. |
| Beale and Leather (2005)(43) | Report | UK | n/a | General healthcare | ‘Working relationships’ | Guide by the Royal College of Nursing to help improve collegiality and avoid unprofessional behaviours in the nursing workplace. Presents team and individual assessment tools to improve team and individual behaviours. Also explores what behaviour may or may not be bullying. |
| Blackstock et al. (2022)(44) | Review | n/a | 15 resources | General healthcare | Incivility | A review which develops an ecological model to understanding co-worker incivility experiences of new graduate nurses. |
| Blakey et al. (2018)(45) | Editorial | Australia | n/a | Trainees (general) | Bullying | Explores in in-depth manner the reasons why bullying interventions may and may not be effective, or even counterproductive, with trainees. |
| Bry and Wigert (2022)(132) | Qualitative study | Sweden | 13 neonatal nurses | Neonatal intensive care | Organisational climate and interpersonal interactions | Explores the organisational climate and type of interpersonal interactions experienced by registered nurses in the neonatal unit. As part of this it explores the impact of incivility. |
| Carter et al. (2013)(46) | Mixed methods survey + interview | UK | Seven NHS trusts in NE England, comprising 2950 NHS staff and 43 in qualitative telephone interview | General healthcare (acute, primary and mental) | Bullying | Survey with qualitative elements also which explores prevalence and impact of bullying in the NHS. Largely focuses on prevalence but also has rich description from qualitative findings of impacts of bullying. They find bullying is prevalent in the NHS with 20% of staff reporting being bullied. |
| Ceravolo et al. (2012)(47) | Intervention | USA | 4032 practicing nurses, 1160 students and faculty | Acute care | Lateral violence | This intervention used culture-change and communication enhancing workshops to decrease lateral violence in a five-hospital integrated health and care system. |
| Chadwick and Travaglia (2017)(48) | Systematic Review | Australia | 62 studies | General healthcare | Bullying | Explores what types of behaviour comprise bullying, the contributing factors, and factors that can help address it too. |
| Churchman and Doherty (2010)(50) | Qualitative interviews | UK | 12 nurses | Acute care | ‘Challenging doctors’ practice’ | Unpacks when nurses would be willing to challenge doctors’ practice without fear of reprisal or conflict and the interprofessional status of nurses vs. doctors. |
| Credland and Whitfield (2022)(54) | Qualitative study | UK | 14 interviews | Paramedics | Incivility | A qualitative study of the experience of paramedics with incivility in the UK. It highlights the impact on clinical decision making and wellbeing. |
| Demarco, Roberts, and Chandler (2005)(56) | Intervention | USA | 5 graduate nursing students | Acute care | Group cohesion | Pilot study investigating a writing group’s ability to build group cohesion. The group writing drew on the ”Amherst Writers and Artists” method which did not focus on UB but nonetheless was intended to help cope with it. |
| Dimarino (2011)(57) | Intervention | USA | Unclear | Ambulatory setting | Lateral violence | Reporting of one ambulatory surgery centre’s approach to reducing lateral violence through education about lateral violence, and zero tolerance policies. Did not test effectiveness. |
| Dixon-Woods et al. (2019)^139^ | Intervention | USA | 67 employees (20 senior, 47 frontline) | Acute care | Disruptive behaviour | This study sought to improve employee’s ability to speak up about transgressive and disruptive behaviour at a John Hopkins Medicine hospital. |
| Edwards and O’Connell (2007)(59) | Narrative review | UK | n/a | Nurse education | Bullying | Delves deeply into bullying and its aetiology and presents recommendations for practice to help tackle it. |
| Efe and Ayaz (2010)(60) | Mixed methods survey + focus groups | Turkey | 206 nurses in one hospital and four focus groups with 16 total participants | Acute care | Mobbing | Primarily investigated the prevalence of mobbing but also presents rich qualitative data regarding the causes of mobbing and what might be done about it. Suggests assertiveness training and need to solve communication issues. |
| Embree, Bruner and White (2013)(61) | Intervention | USA | 143 nurses | Acute care | Lateral violence | Investigates effectiveness of a cognitive rehearsal education intervention on nurse to nurse lateral violence. |
| Gamble Blakey et al. (2019)(62) | Narrative review | No specific country | 38 articles | General healthcare | Bullying | Explores interventions to help students with bullying and explores catalysts for bullying, how policies may affect bullying, how targeting of specific groups affects interventions, framing to improve effectiveness, and skills of facilitators. |
| Griffin (2004)(63) | Intervention | USA | 26 newly licensed nurses | Acute care | Lateral violence | Reporting an intervention assessing use of cognitive rehearsal techniques to reduce lateral violence between newly licensed nurses. |
| Hawkins, Jeong and Smith (2019)(65) | Integrative review | No specific country | Sixteen papers included | Acute care | Negative workplace behaviour | Delves into the conceptual differences in terms dealing with negative workplace behaviour, precipitating factors, and interventions to reduce such behaviour. |
| Hawkins et al. (2022b)(133) | Intervention | Australia | 230 nurses from 12 units in four hospitals | Acute care | Negative workplace behaviour | Examined experiences of negative workplace behaviour and ways of coping with nursing staff before and after educational workshops. They did not find statistically significant results. |
| Hawkins et al. (2022a)(134) | Qualitative study | Australia | 13 nurses | Acute care | Negative workplace behaviour | A qualitative study exploring negative workplace behaviour with nurses and why it occurs. It finds that while some individuals can be more inclined than others to do so, it is facilitated by organisational influences. |
| Hemmings et al. (2021)(66) | Report | UK | n/a | General healthcare | Non-inclusive culture | Report by NHS Employers and the Nuffield trust focused on means of attracting and keeping a diverse NHS workforce. Explores a number of case studies in rich detail as well as interventions used to improve diversity and why they may have failed. |
| Hickson et al. (2007((67) | Intervention | USA | Unknown | Acute care | Unprofessional behaviours | Outlines and reviews the Vanderbilt approach to identifying, measuring, and addressing UB using four graduated interventions. |
| Hughes (2003)(68) | Editorial | UK | n/a | Acute care | Bullying | Looks at strategies at both an individual and organisational level to reduce bullying and its impact in healthcare. |
| Hutchinson et al. (2008)(69) | Outlining theoretical model | No specific country | n/a | General healthcare | Bullying | Investigates a novel model of bullying in the nursing workplace which includes organisational antecedents and consequences of such behaviour. Goes into detail about the organisational climate that enables bullying. |
| Hutchinson et al. (2010)(70) | Testing theoretical model | Australia | 370 nurses | General healthcare | Bullying | Confirms the strengths of the relationships outlined in the model above – as such, explores several causes of bullying and how these may work. |
| Işik et al. (2020)(71) | Qualitative interviews | Turkey | Two hospitals with 14 interviews with perioperative nurses | Acute care | Communication failure | Qualitatively investigates reasons for communication failures in perioperative environment, and as part of this looks at intra-team violence and other causes of communicative failures that might lead to negative behaviour. |
| Johnson et al. (2020)(74) | Simulation | USA | 58 students | General healthcare (students) | Incivility | An RCT simulation investigating the impact of an occurrence of incivility on clinical performance, teamwork and emotions. It found that 66% of the experimental group made a major error in their cardiopulmonary resuscitation performance. |
| Leiter et al. (2011)(81) | Intervention | Canada | 1,173 workers across 41 units | Acute care | Civility | Assesses the effect of 6 months of the CREW intervention and found that greater group x time interactions were found in the intervention group for civility, supervisor incivility, respect, cynicism, job satisfaction, and management trust, and absences. |
| Lewis (2018)(82) | Report (mixed methods) | UK | 110+ employees interviewed, 1488+ surveyed (1100 full responses) | Emergency department | Unprofessional behaviours | Report on grievances about staff behaviour at an NHS Ambulance Trust. Explores unreasonable management behaviours and their causes, incivility and disrespect, rudeness, and threatening behaviour. Delves deeply into the qualitative data. |
| Longo and Hain (2014)(83) | Editorial | No specific country | n/a | Nephrology | Bullying | With a focus on dialysis centres, talks about healthy work environments that can counteract inappropriate work behaviours, including collaboration, effective decisionmaking, authentic leadership, etc. |
| Lovejoy-Bluem (2016)(84) | Editorial | No specific country | n/a | Neonatal | Incivility | Discusses incivility in the neonatal ICU, and spends most of its words on discussing strategies to reduce incivility. Also walks through an example of how small incivility can lead to large clinical impact. |
| Manton (2017)(85) | Editorial | No specific country | n/a | Emergency | Bullying | An editorial exploring bullying, its effect on the work environment, and strategies to reduce it. |
| Markwell et al. (2015)(86) | Editorial | Australia | n/a | Students (general healthcare) | Performance management vs. bullying and harassment | Breaks down performance management and how in some cases it may be considered bullying while in other cases it might not be. Presents briefly some strategies to tackle this issue. Also presents a couple ‘vignettes’ as illustrative examples. |
| McKenzie et al. (2019)(87) | Intervention | Australia | 21 healthcare staff pre-to-post | Acute care | Unprofessional behaviour | Investigated factors affecting implementation of a multistrategy intervention using education, reporting systems with graduated intervention processes, safety champions, and action plans, to tackle unprofessional behaviour. |
| Miller and Chen (2021)(89) | Editorial | No specific country | n/a | Students (general healthcare) | Microaggressions | Tackles strategies to address microaggressions at a programmatic and institutional level, as well as informally as an individual. |
| Naylor, Boyes and Killingback (2022)(136) | Qualitative study | UK | 6 physiotherapists | Acute care | Incivility | Investigates the impact of incivility on physiotherapists working in the acute hospital setting using Interpretative Phenomenological Analysis. |
| NHS Employers (2016)(92) | Report | UK | n/a | Paramedics | Bullying | Explores many real-world strategies implemented by NHS ambulance trusts and how these strategies have been received. |
| Nikstatis and Simko (2014)(93) | Intervention | USA | 21 nurses | Acute care | Incivility | A quantitative pilot study using a 1-group pre and post intervention test design to assess a 60 minute educational programme. |
| O’Connell, Garbark and Nader (2019)(94) | Intervention | USA | 76 participants | Military | Lateral violence | A quantitative exploration of nurses’ perceptions of lateral violence within a military setting and the impact of an education, cognitive rehearsal, and role play intervention. |
| O’Keefe, Brennan and Doherty (2022)(95) | Description of an intervention | Ireland | 203 course participants | General healthcare | Conflict | Describes an intervention, an instructional course, designed to improve professionalism, and how it is implemented. Course content focuses on conflict management. |
| Osatuke et al. (2009)(96) | Intervention | USA | 647 post-intervention CREW participants and 680 comparison (total 34 workgroups) | Acute care | Civility | Preliminary evaluation of a nationwide Veterans Health Administration interventions called CREW across 23 sites. |
| Owens, Singh and Cribb (2019)(97) | Editorial | UK | n/a | General healthcare | Professionalism | Explores the impact of austerity on professional working environments in healthcare in the UK context. Suggests mechanisms for how austerity signals to workers and organisations the degree to which professionalism is valued. |
| Pavithra et al. (2022)(98) | Qualitative study | Australia | 1636 survey respondents (various healthcare staff) | Acute care | Unprofessional behaviour | Explores qualitative responses to two open-ended survey questions delivered to seven hospitals in Australia. It found that a perceived lack of organisational action erodes confidence in hospital leaders and ability to address and mitigate UB effectively. |
| Parker et al. (2016)(99) | Intervention | USA | Unclear / organisation-wide | Acute care | Horizontal violence | Explores how nurses at an acute care hospital were able to implement multiple interventions to reduce horizontal violence prevalence in the organisation. These included 13 total organisational, leadership, and individual level strategies. |
| Phillips et al. (2018)(100) | Integrative review | No specific country | 38 studies | General healthcare | Incivility | Explores quite deeply the different antecedents for incivility in healthcare. It also delves into preventative strategies and leadership behaviours, education, and systems thinking. |
| Purpora and Blegen (2012)(101) | Theoretical model | No specific country | n/a | General healthcare | Horizontal violence | Describes a theoretical model of how horizontal violence comes to impact the quality and safety of patient care. Model includes oppression and explores the concept in relation to oppression, as well as how this leads to internalisation and affects communication etc. |
| Rocker (2008)(102) | Narrative review | Canada | n/a | General healthcare | Bullying | A narrative review which explores strategies to prevent nurse to nurse bullying, mitigate its effects, legal responses in Canada, and its origins. |
| Royal College of Nursing (2014)(103) | Report | UK | n/a | General healthcare | Bullying and harassment | Guidance materials from the RCN oriented towards organisations for preventing bullying and harassment in healthcare. Provides information about the law, the impact of bullying, prevention and reactive strategies and sample policies and recommendations for investigating incidents. |
| Royal College of Surgeons of England (2021)(104) | Report | UK | n/a | Acute care | Disruptive behaviour | Guide from the RCSE for surgeons delving deeply into how to tackle disruptive behaviour, as well as what causes it. |
| Sillero and Buil (2021)(109) | Qualitative interviews | Spain | 16 healthcare professionals | Acute care | Interprofessional collaboration | Examines the interactions between doctors and nurses and how they can be kept civil. Hence it sheds light on how interprofessional collaboration as a cause can lead to incivility. |
| Speck et al. (2014)(111) | Intervention | USA | Three hospitals | Acute care | Unprofessional behaviour | Assessment of a professionalism committee approach to tackling unprofessional behaviour across three large teaching hospitals. |
| Stagg et al. (2017)(112) | Intervention | USA | 10 nurses | Acute care | Bullying | Assessed the effectiveness of a 2-hour cognitive rehearsal programme, 6 months after completion. |
| Stagg et al. (2011)(113) | Intervention | USA | 20 nurses | Acute care | Bullying | Evaluated a workplace bullying cognitive rehearsal programme. |
| Stevens (2002)(114) | Intervention | USA | Unclear | Acute care | Bullying | Informally explores the impact of a multi-strategy anti-bullying intervention in a large teaching hospital, delivered mostly through workshops for education, policies, supervisor training, and more. |
| Stone, Philips and Douglas (2019)(115) | Qualitative interviews | Australia | Six female doctors who were in training when abused | General healthcare | Sexual harassment and assault | Discusses in depth the assaults that happened to several female doctors in the workplace by other doctors and how they tried to address what happened. |
| Tame (2012)(116) | Qualitative interviews | UK | 23 perioperative nurses at one NHS Trust | Acute care | Horizontal violence | Qualitative study investigating experience of horizontal violence at an NHS Trust and the causes for it, the personal impacts, and touches upon strategies to resolve it. |
| Taylor and Taylor (2018)(117) | Editorial | No specific country | n/a | General healthcare | Horizontal violence | A discussion paper based on a prior review and experience of the authors which argues that horizontal violence is a quality improvement concern. As part of this it explores strategies such as codes of conduct and how these should be implemented across different contexts. |
| Thorsness and Sayors (1995)(118) | Intervention | USA | Approximately 100 surgical staff | Acute care | Conduct issues | Evaluation of a programme adopting a systems approach to cultivating a positive work environment for perioperative staff members |
| Tuffour (2022)(120) | Qualitative interviews | UK | Five nurses from sub-Saharan Africa | Mental health | Discrimination | Goes into depth on the experience of nurses from Africa working in the UK NHS Context. As part of this it explores their experience of discrimination and marginalisation and the ‘snowy peak’ syndrome. Paper makes recommendations for how to decrease discrimination in the NHS. |
| Villafranca et al. (2017)(122) | Narrative review | No specific country | n/a | Acute care | Disruptive behaviour | Explores the antecedents of disruptive behaviour at intrapersonal, organisational, and interpersonal levels. It also discusses the prevention and management options to tackle it. |
| Warrner et al. (2016)(123) | Intervention | USA | 60-bed orthopaedic inpatient unit incl. management | Acute care | Incivility | Evaluates an intervention comprising awareness education, cognitive rehearsal, and which included management. |
| Weaver (2013)(124) | Editorial | No specific country | n/a | General healthcare (graduates) | Horizontal violence and bullying | Tackles the chain of violence in nursing from one generation to another and seeks to understand how to reduce it from individual, school, and organisational levels. |
| Wild et al. (2015)(29) | Editorial | UK | n/a | Acute healthcare (trainees) | Undermining and bullying | Editorial which looks at the definitions of undermining and bullying, implications of such behaviours towards trainees, and how such behaviours can be tackled. Towards the end it presents specific recommendations. |
| Wilson (2016)(127) | Narrative review | No specific country | n/a | General healthcare | Bullying | Narrative review that explores the root causes, types of perpetrators, behaviours of bullying, and interventions to combat it. |
| Zhang and Xiong (2019)(128) | Review | No specific country | 44 articles | General healthcare | Horizontal violence | Review focused on the impact of nursing and coping strategies that help tackle the effects of bullying as well as reduce it. These include educational interventions, leadership styles, and enlightenment. |
