# Supplementary material for: Drivers of unprofessional behaviour between staff in acute care hospitals: a realist review
Source: BMC Health Serv Res. 2023 Nov 30;23:1326. doi: 10.1186/s12913-023-10291-3 (PMC10687856; doi:10.1186/s12913-023-10291-3)
Supplement: Supplementary file 5 — Additional file 5. Stakeholder feedback summary. [file 12913_2023_10291_MOESM5_ESM.docx]

Additional File 5. Stakeholder Feedback summary

Societal context

- Stakeholders highlighted ongoing societal contexts throughout our review, including #BLM, #MeToo and the bullying and harassment scandals occurring at the highest levels of UK government. These can normalise bullying and harassment and fail to set a good example for healthcare organisations implementing initiatives to address UB. While identified literature for our review did not reference these societal shifts, we have sought to place our review within this broader societal and historical context.

Staff from minoritized backgrounds

- We ran a specific stakeholder spotlight session that helped us hone our language when discussing staff with protected characteristics throughout this review. This session highlighted that different people prefer different terms: there was no single consensus. Some people found the use of the term BME (black, minority ethnic) problematic, whereas others found the term ‘minoritised groups’ problematic. As a result, we are as specific as possible when we are referring to particular groups throughout this review.

Contributors feedback

- We presented an early understanding of contributors to our stakeholders in January and May 2022. We received feedback that some of our understanding was too focused on the individual. This shaped our direction for the refined analysis in Step 2 of the project going forward, in which we focused on aspects that were within an organisation’s control to change. For example, in our initial theory of ‘causes’ of UB, we had many factors considered as individual-level, including professional or personal backgrounds, job demands and ability to cope. However, in our refined understanding, we now acknowledge that many of these factors are a function of the organisational environment rather than resulting from individual differences.
- The stakeholder group also helped shape our language, moving from an understanding of ‘causes of UB’ – which was too deterministic – to ‘contributors to UB’.
- Our spotlight session highlighted that trust in management is often particularly low among people from minoritised backgrounds. Often, there is a passing of the torch when it comes to accountability and often management do not want to acknowledge racism or sexism within their organisations. This lowers trust in management and helped refine CMOCs that refer to trust in management.

Example stakeholder meeting notes including team input with respect to contributors

| **Unprofessional Behaviours Stakeholder Meeting 1 18^th^ January 2022 (After Step 1 theories formulated)**  **Capturing, incorporating and actioning stakeholder feedback**  **Working Document** | | | |
| --- | --- | --- | --- |
| Areas discussed | Suggestions for how to resolve (JA) | Other thoughts (Team members) | Actions taken (where they have been taken) - Highlighted items are those to be revisited |
| Causes of unprofessional behaviours | | | |
| Culture - culture as it operates across the various levels? | Added but Needs discussion | Yes indeed but not sure this is something we can nail down now – we have the apples and orchards idea already – so again something to continually keep under review and will be part of C / BIG C – JM  Or is it that we identify and name different types of culture, the way they normalise certain behaviours and their implications e.g. permissive cultures, perfectionist cultures...- RA  Will be interesting to see how this develops; that workplace and societal culture is multi-faceted and inter-linked in reality is often poorly represented in the literature - AJ | Culture has been added to diagram but will be refined using step 2 literature and beyond- JA  Happy to help think/write about culture and sub-cultures across organisations (RM) |
| What is acceptable/tolerance spans team and organisational levels | This has been added in both sections. | n/a | This has been duplicated across both levels now- JA |
| Belief in meritocracy/superiority by leaders | Needs discussion | Yes discussion will help us all – could bring these into next big meeting perhaps? - JM  Agreed- point for expanding- RA  Interesting point – it may be of interest that in quality & safety literature managers perceptions of staff is generally out of kilter with reality e.g., managers believe/perceive staff to be more positive about psychological safety than they are - AJ | JA to corroborate (or not) with data from step 2- RA |
| Team factors – quality of line management, peer support, trust | Added trust and line management to diagram. Peer support included in individual factor as perception of social support. | See my comment on team factors in the recent document shared (030222). I have done a realist review on teamwork and there might be useful mechanisms to borrow from it- RA | JA to look into RA’s realist review on teamwork- JA |
| Personality – views, attitudes, and perceptions | Added to diagram | We need to be careful with the personality aspect- RA | Need to explore further along with general ‘traits’ discussion – perhaps one for a spotlight session- JA |
| Leadership  – lack of insight by leaders into structural inequalities in their own organisations (e.g. a ‘post-racist’ narrative)  - the significant role played by leadership in ‘setting the tone’ of an organisation shouldn’t be under-estimated | Needs discussion | Yes discussion will help us all – could bring these into next big meeting perhaps? - JM  This could be fun but think these need collapsing first because quite repetitive and maybe we need to create causal links with culture- RA | JA to refine CMOcs with input from MP, RA and JM- RA |
| Pressure from patient – job demands? | Yes, was intended to be included as an aspect of ‘job demands’ | Already included | Included as ‘sub-aspect’ of job demands – these to be broken down further in step 2/refinement- JA |
| Media coverage and its influence | Added to ‘public pressure’ | Already included | Included as part of public pressure- JA |
| Different professions interacting | This was already included as ‘collaboration across departments’ | Already included | Already included- JA |
