# Supplementary material for: Drivers of unprofessional behaviour between staff in acute care hospitals: a realist review
Source: BMC Health Serv Res. 2023 Nov 30;23:1326. doi: 10.1186/s12913-023-10291-3 (PMC10687856; doi:10.1186/s12913-023-10291-3)
Supplement: Supplementary file 6 — Additional file 6. Contributors, their programme theories, and illustrative quotes. [file 12913_2023_10291_MOESM6_ESM.docx]

# Additional File 6. Contributors, their programme theories, and illustrative quotes.

| **Contributor category** | **Sub-contributors within category** | **Programme theories** | **Example of illustrative data for programme theories** |
| --- | --- | --- | --- |
| Workplace disempowerment | - Organisational hierarchies and working in a disadvantaged group - Physical environment - Perceived unfairness | 1. If staff work in a disempowered position, such as at the bottom of a hierarchy (C), then this can increase the likelihood of experiencing, exhibiting or being impacted by UB (O) because it can make staff an easier target (M12).  2. If staff work in a disadvantaged group (C) then this can lead to displacement of aggression to others (O1) and a feeling of being undervalued (O2) due to internalisation of oppression (M4).  3. If staff work in a disempowered position where there does not seem to be a level playing field (C1) or work in a physically uncomfortable environment (C2), then this can cause people to externalise these frustrations, increasing proclivity to engage in UB (O2) because staff feel like they are being treated unfairly (M3) experience frustration (M5) and have a reduced ability to cope (M18/O1). | 1. “*The presence or absence of these variables could influence bullying ratios, i.e., when bullies weigh the potential costs and personal benefits of their actions based on the particular characteristics of their victims. In this sense, certain groups are considered more vulnerable than others (e.g., women or junior employees).”* (1). 2. “… *unprofessional behaviours appear to be tolerated and internalised as normalised behaviour, creating a self- perpetuating cycle of negative behaviours, negative sub- cultures, and self-isolation among victims*” (2) 3. “*In the model, horizontal violence represents the harmful*   *behaviour oppressed nurses are at risk for engaging in to relieve mounting frustration from working in hierarchical hospitals where they have great responsibility but little power*.” (3). |
| Enablement of harmful cultures and workplace processes | - Organisational change - Demanding work environments and lack of resources - Culture of pressure - Lack of role clarity - Complicit and permissive leadership - Negative role modelling and prior learned behaviour - Authoritarian culture - Lack of organisational accountability - Organisational deafness - Cliques | 4. If staff experience a period of organisational uncertainty, such as organisational change (C) or they experience a lack of job resources (C2), then this can lead to conflict and UB (O) because staff perceive their job is at risk and an increase in competitive attitudes ensues (M8).  5. If high job demands are experienced regularly in the absence of adequate resources (C1) then escalation of conflicts are more likely (O2), because there is both a high-pressure environment (M5) and reduced ability to communicate effectively (M14/O1).  6. If staff experience a lack of resources, or high job demands that increase pressure (C) then this may lead to an inability to cope with the impact of UB, (M18/O1), because communication with co-workers can be inhibited (M14) meaning it is difficult to build relationships with colleagues (M16) which can reduce feeling of social support (M17).  7. If staff are disadvantaged by organisational processes outside of their control, such as a lack of role clarity or high job demands (C) then this may increase levels of curtness in communication (O2) because they begin to feel pressured and their tasks become rushed (M5), reducing ability to communicate effectively (M14/O1).  8. If a workplace has a prevalence of UB and leaders/ managers are not seen to address it by being complicit or laissez-faire (C1), then this can cause staff to engage in UB (O) and reduce trust in leadership (O2), because UB is perceived as accepted and normalised (M13).  9. If a workplace has a high prevalence of UB and leaders/ managers are not seen to address it (C) or role model it themselves (C2) then there is an increasing likelihood of others engaging in UB (O) and a loss of trust in leadership (O2) because the impression can be given that engaging in incivility and other UBs is the norm (M13) which reduces perceived risk for instigators (M12), and ability to speak up for victims (M21).  10. If UB is prevalent in a workplace and managers are not seen to address it (i.e. lack accountability) (C1) or even negatively role model such behaviours themselves (C2) then this can reduce psychological safety (M21/O1) and reduce trust in management (O2) because staff sense that the organisation is deaf (i.e. that they do not care about UB and do not act upon reporting of UB) (M10) which creates an intimidating culture (M20). | 1. “*In competitive environments, organizational restructure or periods of rapid change may create opportunities for individuals to engage in the misuse of legitimate authority for furthering self-interest or career opportunities*.” (4). 2. “… *some behavioural interactions associated with bullying are because of excessive job demands. What is well-defined is that ‘friction or anger’ between colleagues is widely reported as high across SWAST and extremely high by 111 staff*” (5) 3. “*The stress caused by the accelerated pace and pressure to complete the activity can decrease communication quality, as explained by the following participants: Surgeon 3: ‘I cannot work on the patient the way I want, and it affects my work’. Anaesthesiologist 2: ‘Of course, sometimes you feel understaffed’. Surgeon 1: ‘The operating room is like this . . . everything goes fast, practically every-thing must be done immediately, without waiting, with tension’*” (6). 4. “*Stress and pressures of the ED can cause inappropriate or unprofessional responses that could trigger behaviours affecting colleagues’ relationships and task responsibilities*.” (7). 5. “*Organisations in which managers do not act on bullying complaints and where co-staff do not act when they witness bullying can create a social climate in which bullying is tolerated and even seen as the norm (Hutchinson et al 2008). As a result, bullying can become the accepted ‘way we do things around here’, with some staff adopting the attitude ‘It was done to me, so I’ll do it to others’*.” (8). 6. “*Staff raised numerous examples of manager behaviour that they deemed unreasonable. Issues such as being treated differently compared to colleagues such as “some staff get pulled up, others don’t” was a frequent occurrence while Serious Investigations (SI) were believed by some staff to be seemingly deployed inappropriately*” (5). 7. “*Many at the sharp end expressed frustration that although the organization invited staff and faculty to speak about concerns, it appeared to lack an authentic capacity for listening or a full commitment to address concerns: ‘[There are] pockets of historically weak leadership where we learn that there have been ongoing issues for years that people [managers] have been either unwilling or uncomfortable addressing. So after a while you just stop talking about it’*” (9). |
| Inhibited social cohesion and support | - Lack of social support (e.g. due to time pressures) - Shift or agency working - Reduced ability to communicate effectively | 11. If staff work in shifts (C1) and/or lack social support (C2), then this can reduce the ability to cope when experiencing UB or workplace stressors (M18/O1) and reduce self-confidence (M19/O2) which can worsen the impact of UB on health and wellbeing (O3) because these can reduce ability to build social connections (M16) and lessen feeling that one is socially supported (M17).  12. If staff work in a high-pressure environment or in a culture of intimidation (C) then this can lead to reduced ability to build social connections (M16/O1), a reduced ability to determine social norms (M15/O2), and a reduced sense of social support (M17/O3) because there is a reduced ability to communicate effectively (M14). | 1. “*Some staff cannot build relationships with their line manager because of rotas and might have very infrequent encounters with them*.” (5). 2. “*Firstly, many participants relayed being subject to one-off experiences, such as being spoken to rudely in meetings or being shouted at for not having completed a task. When context was provided, most people attributed this to managers being ‘stressed’ or ‘pressured’ due to excessive workloads generated by Covid-19 or winter crises*.” (10). |
| Reduced ability to speak up | - Workplace disempowerment - Harmful culture | 13. If staff work in a disempowered position such as at the bottom of an organisational or professional hierarchy (C1) or within a harmful organisational culture (C2) or work in an environment with exposure to negative cliques (C2) then this can inhibit willingness to speak up (M21/O1) and reduce ability to communicate (M14/O2) because staff experience a sense of intimidation and reduced psychological safety (M20). | 1. “*Steep hierarchies can give rise to feelings of intimidation which can impair communication, especially on difficult matters, such as raising concerns about patient safety. It was acknowledged that most behaviours that made people feel undermined were unintentional. Marked hierarchies may magnify the impact of relatively slight behaviours or comments if those affected are less able to voice how it made them feel*.” (11). |
| Lack of manager awareness and urgency | - Workplace disempowerment - Harmful culture | 14. If a reduced sense of psychological safety leads people to not speak up (C) then strategies to address UB are not able to be implemented (M23/O1), reducing trust in leadership (O2) because managers are not aware that UB is taking place (M22). | 1. “*Furthermore, ‘bad news' is rarely passed upwards by front-line managers who feel it is better that senior managers do not ‘hear bad news’. This often means senior managers/executives are unaware of what is happening at the front line. Subsequently this can result in ‘why bother' attitudes from staff – a form of confirmation bias – because some staff perceive action plans will be largely ineffective as senior managers are too detached from employees’ everyday lives*.” (5). |

## References

1. Ariza-Montes A, Muniz NM, Montero-Simó MJ, Araque-Padilla RA. Workplace bullying among healthcare workers. Int J Environ Res Public Health. 2013;10(8):3121–39.

2. Pavithra A, Sunderland N, Callen J, Westbrook J. Unprofessional behaviours experienced by hospital staff : qualitative analysis of narrative comments in a longitudinal survey across seven hospitals in Australia. BMC Health Serv Res [Internet]. 2022;1–15. Available from: https://doi.org/10.1186/s12913-022-07763-3

3. Purpora C, Blegen MA. Horizontal Violence and the Quality and Safety of Patient Care: A Conceptual Model. Nurs Res Pract. 2012;2012(May 2012):1–5.

4. Hutchinson M, Jackson D, Wilkes L, Vickers MH. A new model of bullying in the nursing workplace organizational characteristics as critical antecedents. Adv Nurs Sci. 2008;31(2):60–71.

5. Lewis D. Workplace Culture at Southwestern Ambulance NHS Foundation Trust. 2018.

6. Sillero AS, Buil N. Enhancing interprofessional collaboration in perioperative setting from the qualitative perspectives of physicians and nurses. Int J Environ Res Public Health [Internet]. 2021;18(20). Available from: https://www.mdpi.com/1660-4601/18/20/10775/pdf

7. Parizad N, Hassankhani H, Rahmani A, Mohammadi E, Lopez V, Cleary M. Nurses’ experiences of unprofessional behaviors in the emergency department: A qualitative study. Nurs Heal Sci. 2018;20(1):54–9.

8. Allen B. Understanding bullying in healthcare organisations. 2015;30(14):259.

9. Dixon-Woods M, Campbell A, Martin G, Willars J, Tarrant C, Aveling EL, et al. Improving Employee Voice about Transgressive or Disruptive Behavior: A Case Study. Acad Med. 2019;94(4):579–85.

10. Kline R. Middlesex University London. 2021. Racism which impacts healthcare staff endangers patient care. Available from: https://mdxminds.com/2021/11/22/racism-which-impacts-healthcare-staff-endangers-patient-care/

11. Academy of Medical Royal Colleges. Creating supportive environments: Tackling behaviours that undermine a culture of safety [Internet]. AoMRC Trainee Doctors’ Group. Academy of Medical Royal Colleges; 2016. Available from: http://www.aomrc.org.uk/wp-content/uploads/2016/09/Creating_supportive_environments_280916-2.pdf
